# Ligand engineering enables an alkaline iron-chromium redox flow battery

**DOI:** 10.1038/s41467-026-77810-8

**Published:** 2026-09-30

**Authors:** Wendong Yang, Pei Liu, Linfeng Wang, Hua Jiang, Shuangbin Zhang, Bin Luo, Xue Long, Yilin Zeng, Xuan Cai, Yifan Zhang, Zuoxuan Gan, Huaiyu Ke, Jinhua Guo, Jiangjiang Duan

**Affiliations:** 1 Wuhan National Laboratory for Optoelectronics, School of Optical and Electronic Information, Huazhong University of Science and Technology, Wuhan, China; 2 State Key Laboratory of Intelligent Manufacturing Equipment and Technology, School of Mechanical Science and Engineering, Huazhong University of Science and Technology, Wuhan, China; 3 Australian Institute for Bioengineering and Nanotechnology (AIBN) & School of Chemical Engineering, The University of Queensland, Brisbane, QLD Australia

**Keywords:** Batteries, Batteries, Energy, Batteries

## Abstract

Iron-chromium redox flow batteries are promising for long-duration energy storage owing to their safety, low cost, and environmental compatibility, but are limited by sluggish chromium(II/III) kinetics and parasitic hydrogen evolution in chromium-based negolytes. Here we re-engineer iron-chromium redox chemistry through chelation-driven molecular design using the strong-field ligand 2,2-Bis(hydroxymethyl)−2,2’,2”-nitrilotriethanol. The ligand reshapes the electronic configurations of iron and chromium centres and tunes their redox potentials, enabling an alkaline iron-chromium redox flow battery with rapid kinetics and suppressed hydrogen evolution. It stabilizes an octahedral hexacoordinate chromium chelate and unlocks an unconventional chromium(III/IV) couple at +0.34 V versus the standard hydrogen electrode, circumventing the Jahn-Teller effects associated with chromium(II/III). Paired with an iron-chelate negolyte based on the same ligand, the cell operates at 1.15 V within the aqueous stability window and remains stable over 500 cycles, achieving 99.0% coulombic efficiency and 75.8% energy efficiency at 100 mA cm^−2^ (80% of state-of-charge). Together with a projected raw-material cost of $17.02 kWh^−1^ and theoretical capacity of 68.07 Ah L^−1^ based on posolyte volume, this work establishes a scalable, cost-effective strategy for grid-scale energy storage.

## Abstract (web summary)

Iron-chromium redox flow batteries are promising for long-duration energy storage but suffer from sluggish chromium(II/III) chemistry. Here, authors use a strong-field ligand to unlock a chromium(III/IV) posolyte and establish a 1.15 V alkaline iron chromium redox flow battery.

## Introduction

Developing intrinsically safe, cost-effective and scalable energy storage systems is essential for enabling the widespread deployment of intermittent renewable energy sources such as solar and wind power^1,2^. Among emerging technologies, aqueous redox flow batteries (RFBs) are particularly attractive owing to their intrinsic safety, decoupled scaling of energy and power, and long operational lifetimes^3,4^. The vanadium redox flow battery (VRFB), as a mature and commercialized RFB technology, has demonstrated long-term durability and scalability in practical installations^5,6^. However, grid-scale implementation of VRFBs remains constrained by the high material cost and limited global supply of vanadium, driving the search for alternative redox chemistries based on earth-abundant elements with improved economic feasibility^7–9^.

**Fig. 1:**
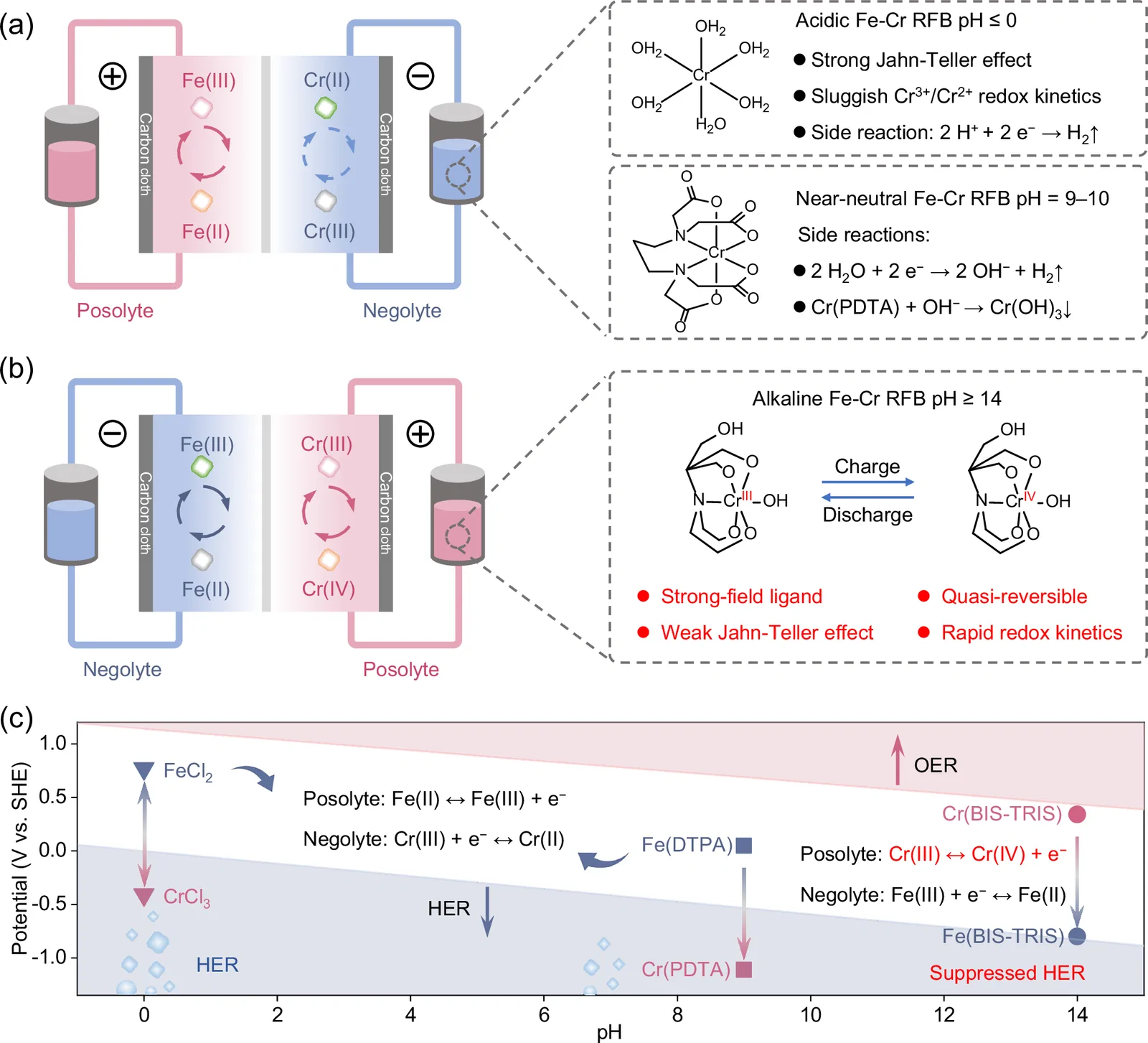
Comparison between conventional iron-chromium (Fe-Cr) chemistries and an alkaline Fe-Cr system. **a** Major limitations of acidic and near-neutral Fe-Cr redox flow batteries (RFBs) utilizing the Cr(II/III) redox pair. **b** Key features of the alkaline Fe-Cr RFB employing the Cr(III/IV) redox pair. **c** Redox potentials of various redox-active species in acidic, near-neutral and alkaline Fe-Cr RFB systems relative to the standard hydrogen electrode (SHE) and the electrochemical stability window of water. The blue and pink shaded regions indicate the thermodynamic regions associated with the hydrogen evolution reaction (HER) and oxygen evolution reaction (OER), respectively. The ligands DTPA, PDTA and BIS-TRIS denote diethylenetriaminepentaacetic acid, 1,3-propylenediaminetetraacetic acid, and 2,2-bis(hydroxymethyl)−2,2′,2′′-nitrilotriethanol, respectively. Source data are provided as a Source Data file.

The iron-chromium (Fe-Cr) RFB has been extensively investigated for half a century because of the low cost and abundance of iron and chromium^10^. Conventional Fe-Cr RFBs operate in strongly acidic environments (pH ≤ 0), employing the Fe(II/III) (+0.77 V versus the standard hydrogen electrode (SHE)) and Cr(II/III) (−0.41 V versus SHE) redox couples^11–13^. Nevertheless, the Cr(II/III) redox reaction has long been recognized as the dominant performance bottleneck because of its inherently sluggish kinetics and the severe competing hydrogen evolution reaction (HER), which collectively limit energy efficiency and power output (Fig. 1a, c)^14,15^. Specifically, the slow kinetics primarily arise from strong Jahn-Teller effects associated with high-spin Cr(II) species, which exhibit a *d*^4^ electronic configuration with uneven occupation of the *e*_g_ orbitals. This phenomenon leads to symmetry-lowering structural changes and splitting of electronic energy levels, thereby hindering electron transfer and further slowing the Cr(II/III) redox process^16^.

Notably, chelation engineering offers a promising strategy for overcoming these limitations of conventional acidic Fe-Cr RFBs. By modifying the coordination environment of metal ions, chelation directly influences the electrochemical properties of metal ions and the thermodynamics of redox reactions, leading to significant improvements in battery performance^17–22^. For instance, the strong-field ligand 1,3-propylenediaminetetraacetic acid (PDTA) stabilizes Cr in a low-spin state via metal-ligand back-bonding, effectively mitigating Jahn-Teller effects and accelerating redox kinetics^15^. Pairing the Cr(PDTA) negolyte with Fe-based complex posolytes enabled the development of near-neutral Fe-Cr RFBs (pH = 9–10)^15,23^. However, the Cr(II/III)(PDTA) redox couple exhibits a low potential (−1.10 V vs. SHE) that falls below the electrochemical stability window of water, making HER unavoidable even in near-neutral conditions (Fig. 1a, c)^24–26^. The resulting HER further elevates the local pH beyond the stable window of Cr(PDTA) at pH = 9–10, leading to irreversible decomposition of chelates and formation of insoluble Cr hydroxide precipitates^26,27^. Consequently, HER remains a persistent bottleneck in both acidic and near-neutral Fe-Cr RFB systems.

Moving from near-neutral or mildly alkaline conditions to strongly alkaline conditions provides a thermodynamically favorable pathway to suppress HER. In addition, alkaline electrolytes are substantially less corrosive, enabling the use of cost-effective materials and improving system durability. However, developing stable metal-active species under strongly alkaline conditions remains challenging, as most metal ions and their complexes tend to form insoluble hydroxide precipitates. Encouragingly, amino-polyhydroxy ligands can stabilize metal ions even under strongly alkaline conditions, owing to their multidentate coordination and the high local electron density of alkoxide donors^28–30^. These features strengthen metal-ligand interactions and suppress hydroxide-induced dissociation, making amino-polyhydroxy ligands promising candidates for constructing stable alkaline RFB systems.

Herein, we develop an alkaline Fe-Cr RFB with reversed posolyte-negolyte roles, enabled by the strong-field amino-polyhydroxy ligand 2,2-Bis(hydroxymethyl)−2,2’,2”-nitrilotriethanol (BIS-TRIS) (Fig. 1b). BIS-TRIS stabilizes an octahedral hexacoordinate Cr(BIS-TRIS) chelate and unlocks an unconventional Cr(III/IV) redox couple. Cr(III/IV)(BIS-TRIS) effectively circumvents the Jahn-Teller effects inherent to Cr(II/III) chemistry, thereby enabling rapid redox kinetics (Supplementary Fig. 1). Benefiting from the utilization of high-valence Cr, the Cr(III/IV)(BIS-TRIS) chelate delivers a high redox potential of +0.34 V vs. SHE, making it a promising posolyte active species. When paired with a Fe(BIS-TRIS) negolyte (−0.81 V vs. SHE), the alkaline Fe-Cr RFB delivers an open-circuit voltage of 1.15 V and sustains stable cycling over 500 cycles with a Coulombic efficiency of 99.0% and an energy efficiency of 75.8% at 100 mA cm^−^^2^ (80% of state-of-charge (SOC)). Furthermore, HER is effectively mitigated in this system due to the intrinsic suppression of HER in alkaline media and the alignment of both redox couples within water’s electrochemical stability window (Fig. 1c). Coupled with the low projected raw material cost ($17.02 kWh^−1^) and high theoretical capacity based on the posolyte volume (68.07 Ah L^−1^), this work provides a scalable and cost-effective pathway to advance Fe-Cr RFBs for grid-scale, long-duration energy storage.

## Results

### Rational design of the alkaline Cr chelate

**Fig. 2:**
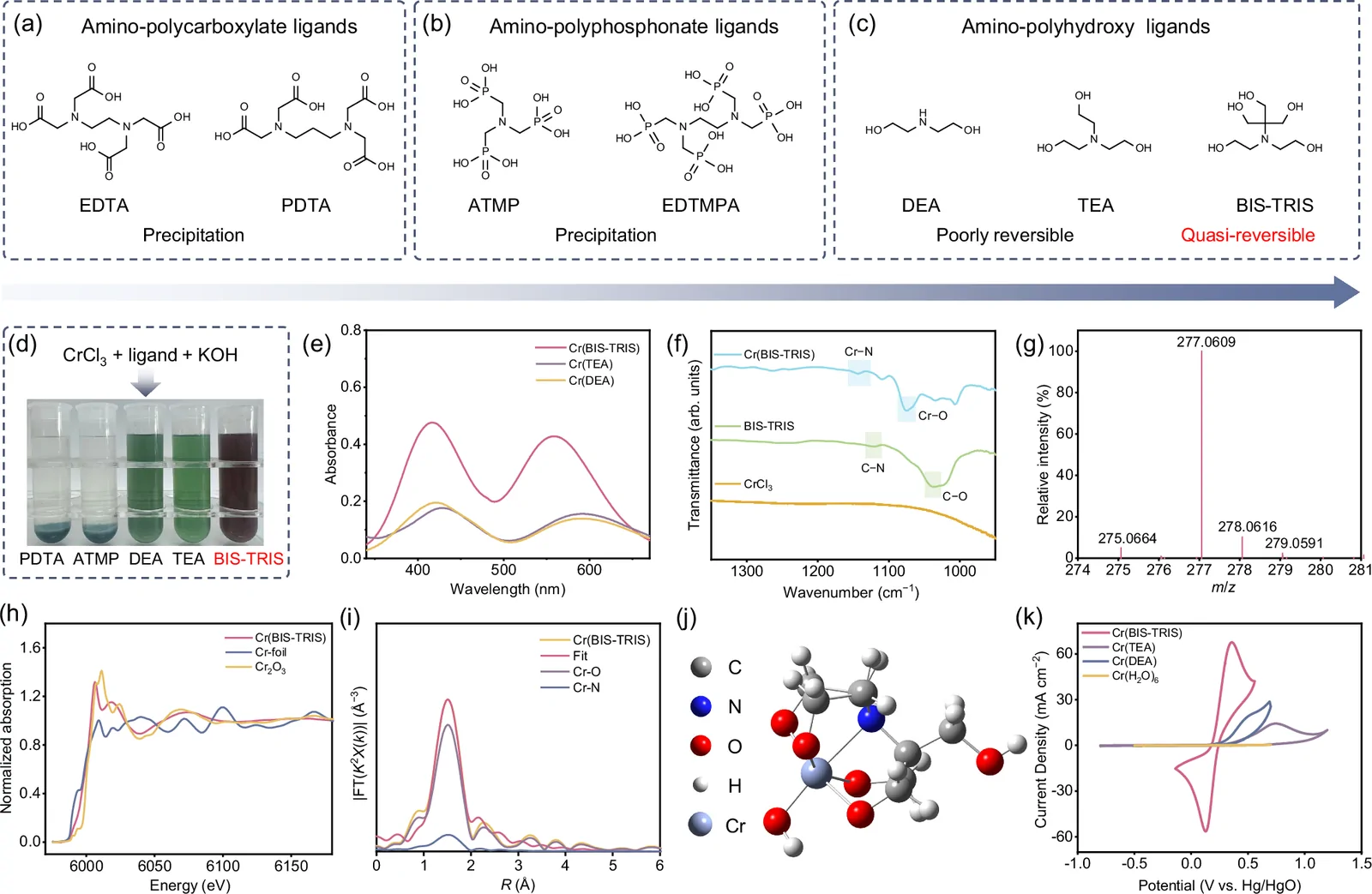
Rational design and structural characterization of the Cr(BIS-TRIS) chelate. **a**–**c** Molecular structures of (**a**) ethylenediaminetetraacetic acid (EDTA) and 1,3-propylenediaminetetraacetic acid (PDTA), (**b**) nitrilotrimethylene triphosphonic acid (ATMP) and ethylenediamine tetra(methylenephosphonic acid) (EDTMPA), and (**c**) diethanolamine (DEA), triethanolamine (TEA), and 2,2-bis(hydroxymethyl)−2,2′,2′′-nitrilotriethanol (BIS-TRIS). **d** Optical images of CrCl_3_ solutions containing different ligands under alkaline conditions (pH = 14). **e** Ultraviolet-visible (UV-Vis) absorption spectra of chromium complexes formed with different amino-polyhydroxy ligands. **f** Fourier transform infrared (FTIR) spectra of Cr(BIS-TRIS), BIS-TRIS and CrCl_3_. **g** Electrospray ionization high-resolution mass spectrometry (ESI-HRMS) spectrum of the Cr(BIS-TRIS) solution. The ion observed at *m/z* 277.0609 is assigned to [Cr(BIS-TRIS)(OH) + 3H]^+^ (C_8_H_19_CrNO_6_^+^, *z* = +1; calculated *m/z*, 277.0612). The Cr(BIS-TRIS) chelate comprises one Cr^3+^ centre, one BIS-TRIS^4−^ ligand and one additional hydroxide ligand (OH^−^). **h** X-ray absorption spectroscopy (XAS) spectra at the Cr K-edge of Cr(BIS-TRIS), Cr foil, and Cr_2_O_3_. **i** Extended X-ray absorption fine structure (EXAFS) spectrum of Cr(BIS-TRIS) and the corresponding fit in *R* space. **j** Structural model of the Cr(BIS-TRIS) chelate. **k** Cyclic voltammetry (CV) curves of 0.5 M Cr(DEA), Cr(TEA), Cr(BIS-TRIS) and CrCl_3_ solutions recorded at 50 mV s^−1^. The potentials were not *iR* corrected. All electrochemical tests were performed at 25 ± 1 °C. Source data are provided as a Source Data file.

The electrochemical performance of chelated electrolytes is primarily governed by their coordination stability and redox reversibility. For an effective alkaline Fe-Cr RFB, the Cr chelate must (i) maintain robust coordination stability in alkaline media without metal-ligand dissociation and (ii) exhibit reversible and kinetically facile redox behavior. Guided by these design principles, we systematically screened a series of multidentate ligands to identify an optimal coordinating framework capable of stabilizing Cr species under highly basic conditions. Because metal centers are generally stabilized by amine-based multidentate backbones, we further classified the candidate ligands according to the nature of their auxiliary donor groups, namely carboxylate, phosphonate, and hydroxyl groups. This classification yields three representative ligand families: amino-polycarboxylate, amino-polyphosphonate, and amino-polyhydroxy ligands. Ten multidentate ligands from each class were evaluated comparatively (Suppl. Figs. 2–4), and representative examples are presented in Fig. 2a–c.

We evaluated the ability of different classes of ligands to stabilize Cr ions under strongly alkaline conditions (pH = 14). Among the three ligand classes, amino-polycarboxylate and amino-polyphosphonate ligands exhibited poor coordination stability, leading to the formation of Cr hydroxide precipitates in alkaline media. This instability arises because carboxylate and phosphonate oxygen donors undergo charge delocalization or dispersion, which reduces the local electron density at individual oxygen sites. Consequently, hydroxide ions can competitively displace the donor atoms, leading to metal-ligand bond cleavage, ligand dissociation, and eventual precipitation of Cr hydroxides (Fig. 2d, Suppl. Figs. 2, 3, 5, 6). Therefore, although these ligands can form stable chelates with metal ions under near-neutral conditions, they are unsuitable for alkaline RFB applications. In contrast, amino-polyhydroxy ligands (e.g., diethanolamine (DEA), triethanolamine (TEA), and BIS-TRIS) displayed greater coordination stability under identical alkaline conditions (Fig. 2d, Suppl. Fig. 4). In strongly basic media, the hydroxyl groups of these ligands are deprotonated to form alkoxide species (–O^−^), in which the negative charge is localized on a single oxygen atom (Suppl. Fig. 7)^19,20,31^. This charge localization increases the local electron density and strengthens the σ-donor capability of the coordinating oxygen, thereby reinforcing metal-ligand bonding and maintaining the structural integrity of the Cr chelates in strongly alkaline solution.

Notably, only the Cr(BIS-TRIS) solution exhibited a deep-violet color, in contrast to the light-green solutions of the other Cr-amino-polyhydroxy ligand complexes, and displayed a distinct ultraviolet-visible (UV-Vis) absorption profile (Fig. 2e). This color difference suggests that Cr(BIS-TRIS) may have a coordination environment different from those of the other complexes. Raman and Fourier-transform infrared (FTIR) spectra revealed that all three ligands coordinate to the Cr center via both nitrogen and oxygen donor atoms (Fig. 2f, Suppl. Fig. 8). However, unlike other amino-polyhydroxy ligands, BIS-TRIS offers more potential donor sites (five hydroxyl groups and one amine), allowing the formation of stable octahedral hexacoordinate chelates with various metal ions (e.g., Fe^3+^, Mn^3+^, Ni^2+^, and Co^2+^) in alkaline environments^32–34^. By analogy, we hypothesized that Cr(BIS-TRIS) adopts a similar octahedral hexacoordinate configuration.

To validate this hypothesis, we employed electrospray ionization high-resolution mass spectrometry (ESI-HRMS) and X-ray absorption spectroscopy (XAS) analyses. ESI-HRMS detected an ion at *m/z* = 277.0609, assigned to [Cr(BIS-TRIS)(OH) + 3H]^+^ (C_8_H_19_CrNO_6_^+^, *z* = +1; calculated *m/z*, 277.0612) (Fig. 2g, Suppl. Fig. 9a–c). The assigned Cr(BIS-TRIS) chelate comprises one Cr^3+^ centre, one BIS-TRIS^4−^ ligand and one additional hydroxide ligand (OH^−^). The observed fragment-ion peaks further support this assignment. Comparative analysis of Cr(DEA) and Cr(TEA) complexes was also performed under identical conditions (Suppl. Fig. 9d–i). Further structural insight into Cr(BIS-TRIS) was obtained from XAS analysis (Fig. 2h, Suppl. Fig. 10). The local coordination environment was analyzed by least-squares fitting of Fourier-transformed extended X-ray absorption fine structure (EXAFS) spectra (Fig. 2i), with fitting parameters summarized in Suppl. Table 1. The EXAFS fit indicates that the Cr(BIS-TRIS) chelate has five Cr-O bonds of 1.95 Å and one Cr-N bond of 1.99 Å. These results support the assignment of an octahedral hexacoordinate structure to Cr(BIS-TRIS) (Fig. 2j). This robust coordination framework enhances orbital overlap and electron delocalization between the metal center and the ligand, thereby facilitating facile electron transfer^20,29,35^. Consistent with this structural advantage, Cr(BIS-TRIS) displays distinct electrochemical characteristics compared with the conventional Cr(II/III) system and other Cr-amino-polyhydroxy ligand complexes (Fig. 2k, Supplementary Fig. 11). Its cyclic voltammogram (CV) exhibits a quasi-reversible redox curve and a higher redox potential of +0.34 V vs. SHE, suggesting that the redox process of Cr(BIS-TRIS) involves higher-valence chromium species rather than the traditional Cr(II/III) transition.

### Electrochemical mechanism of the Cr(BIS-TRIS) posolyte

**Fig. 3:**
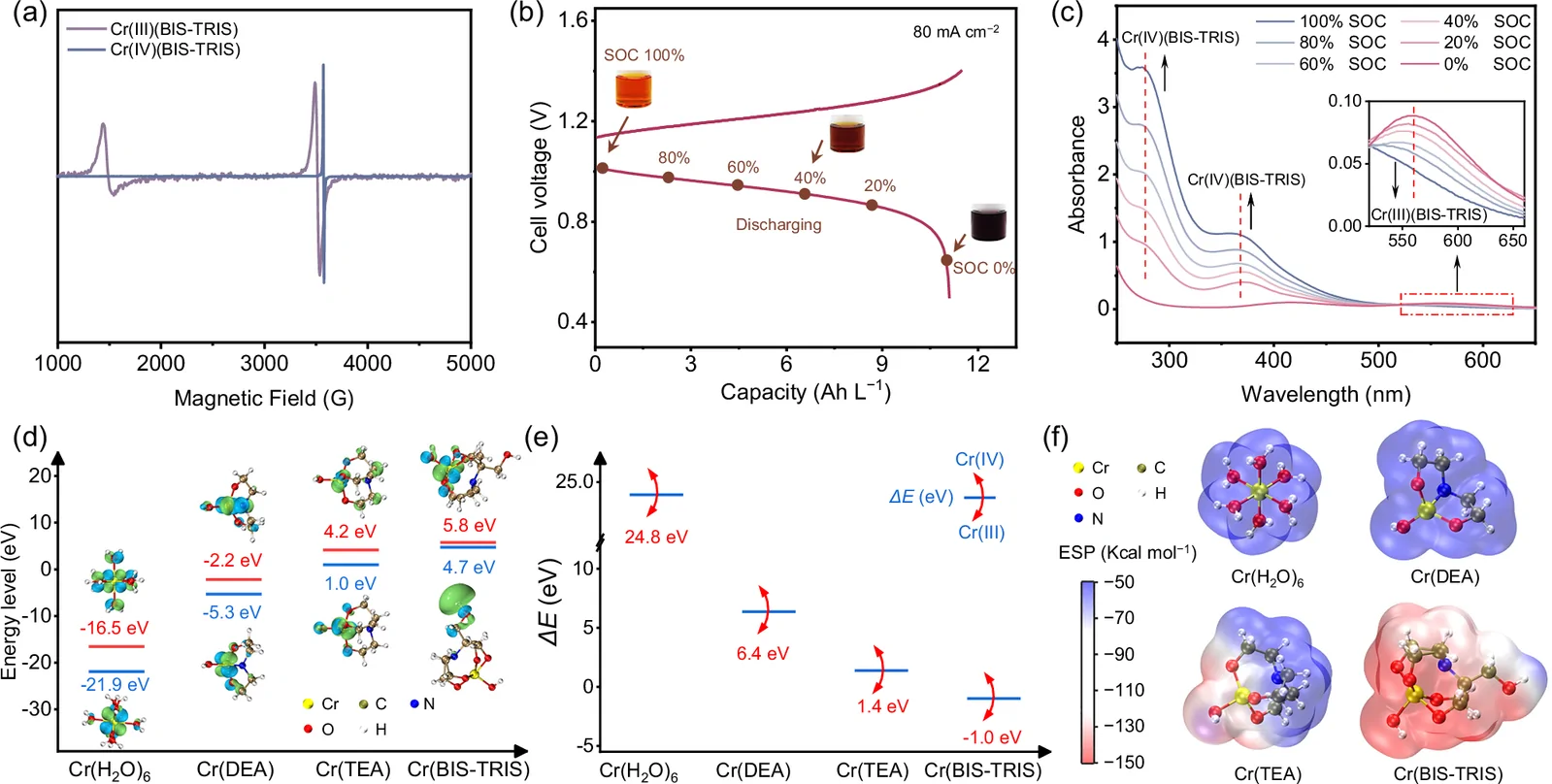
Electrochemical mechanism and theoretical calculation of the Cr(BIS-TRIS) posolyte. **a** Electron paramagnetic resonance (EPR) spectra of charged-state Cr(IV)(BIS-TRIS) and discharged-state Cr(III)(BIS-TRIS). **b** A typical voltage profile obtained using a posolyte-capacity-limiting cell containing 10 mL of 0.5 M Cr(BIS-TRIS) posolyte and 30 mL of 0.5 M Fe(BIS-TRIS) negolyte, together with the corresponding solution photographs at different states of charge (SOC; 0%, 40%, and 100%). **c** Ultraviolet-visible (UV-Vis) spectra of Cr(BIS-TRIS) electrolytes at different SOC. The EPR samples were collected from the Cr(BIS-TRIS) posolyte at 0% and 100% SOC, whereas the UV-Vis samples were collected at 0%, 20%, 40%, 60%, 80%, and 100% SOC. All samples were collected during the fifth charge-discharge cycle at 80 mA cm^−2^ and 25 ± 1 °C. **d** Calculated highest occupied molecular orbital (HOMO) and lowest unoccupied molecular orbital (LUMO) energy levels of different chromium complexes. **e** Calculated energy changes associated with the Cr(III/IV) redox reactions of the different chromium complexes. **f** Calculated electrostatic potential (ESP) maps of the different chromium complexes. All electrochemical tests were performed at 25 ± 1 °C. Source data are provided as a Source Data file.

We investigated the redox behavior of Cr(BIS-TRIS) and discovered that it undergoes a high-valence Cr(III)/Cr(IV) transition, in sharp contrast to the conventional Cr(II)/Cr(III) chemistry. This unconventional redox pathway results in a higher redox potential, making Cr(BIS-TRIS) a candidate posolyte for alkaline RFBs. Electron paramagnetic resonance (EPR) and X-ray photoelectron spectroscopy (XPS) analyses support the assignment of the valence states of Cr(BIS-TRIS). For the Cr(III) state, which has a 3*d*^3^ electron configuration, relatively broad signals were observed at *g* = 1.99 and *g* = 4.90 in the EPR spectra. After charging, a sharp signal at *g* = 1.97 appeared, consistent with the transition to the Cr(IV) state with a low-spin 3*d*^2^ configuration^36–38^ (Fig. 3a). XPS analysis further supports this valence transition. The positive shift of the Cr 2*p* binding energies in the charged electrolyte indicates a more electron-deficient Cr center, consistent with the formation of the higher oxidation state^35^. In the discharged state, the Cr 2*p* spectrum shows dominant peaks at binding energies of 576.1 eV and 585.3 eV, which are assigned to Cr(III)(BIS-TRIS). After charging, additional peaks emerge at higher binding energies of 577.5 eV and 587.2 eV, characteristic of Cr(IV)(BIS-TRIS)^39–41^ (Supplementary Fig. 12). Subsequently, we confirmed its single-electron transfer process using a posolyte-capacity-limiting cell containing 10 mL of 0.5 M Cr(BIS-TRIS) posolyte and 30 mL of 0.5 M Fe(BIS-TRIS) negolyte (Supplementary Fig. 13). The Cr(BIS-TRIS) posolyte (0.5 M, 10 mL) delivered a capacity of 11.5 Ah L^−1^ (Fig. 3b). As the state of charge (SOC) decreased, the posolyte exhibited a distinct color transition from light red to dark purple, a phenomenon governed by the strong-field ligand and different from that observed in acidic Fe-Cr systems^42^. Moreover, UV-Vis spectroscopy further verified the quasi-reversible Cr(III/IV) interconversion. As the SOC increased, the absorption band at 560 nm (Cr(III)(BIS-TRIS)) gradually decreased, while the bands at 274 nm and 365 nm (Cr(IV)(BIS-TRIS)) intensified (Fig. 3c). These spectral evolutions support the quasi-reversible Cr(III/IV) redox transformation within the full-cell configuration.

To provide molecular-level insights into the electrochemical behavior of Cr(BIS-TRIS), we performed density functional theory (DFT) calculations (Supplementary Fig. 14). The optimized computational models are provided separately for Cr(III)(BIS-TRIS) (Supplementary Data 1), Cr(IV)(BIS-TRIS) (Supplementary Data 2), Cr(III)(TEA) (Supplementary Data 3), Cr(IV)(TEA) (Supplementary Data 4), Cr(III)(H_2_O)_6_ (Supplementary Data 5), Cr(IV)(H_2_O)_6_ (Supplementary Data 6), Cr(III)(DEA) (Supplementary Data 7), and Cr(IV)(DEA) (Supplementary Data 8). A narrower gap between the highest occupied molecular orbital (HOMO) and lowest unoccupied molecular orbital (LUMO), together with a higher HOMO energy, may indicate a greater propensity for electron donation and oxidation^43–45^. Figure 3d presents the frontier molecular orbital diagrams and corresponding energy levels of the Cr complexes. Within the computational model, Cr(BIS-TRIS) exhibits the narrowest HOMO-LUMO gap and the highest HOMO energy among the examined complexes, suggesting comparatively favorable electron-donating characteristics. We further calculated the electronic energy differences between the Cr(III) and Cr(IV) states of these complexes. As shown in Fig. 3e and Supplementary Table 2, Cr(BIS-TRIS) exhibits the smallest computed electronic energy difference among the examined complexes, which is qualitatively consistent with its experimentally observed redox behavior. Electrostatic potential (ESP) mapping of the molecular van der Waals surfaces shows that Cr(BIS-TRIS) has a lower surface potential than the other modelled Cr complexes (Fig. 3f). This feature is associated with the high local electron density of the alkoxide groups within the amino-polyhydroxy ligand framework. With four monoanionic oxygen donors, BIS-TRIS exhibits strong electron-donating ability, which is expected to strengthen coordination to the positively charged Cr centre and thereby contribute to the observed chelation stability and redox reversibility^46–48^.

### Alkaline Fe-Cr RFB study

**Fig. 4:**
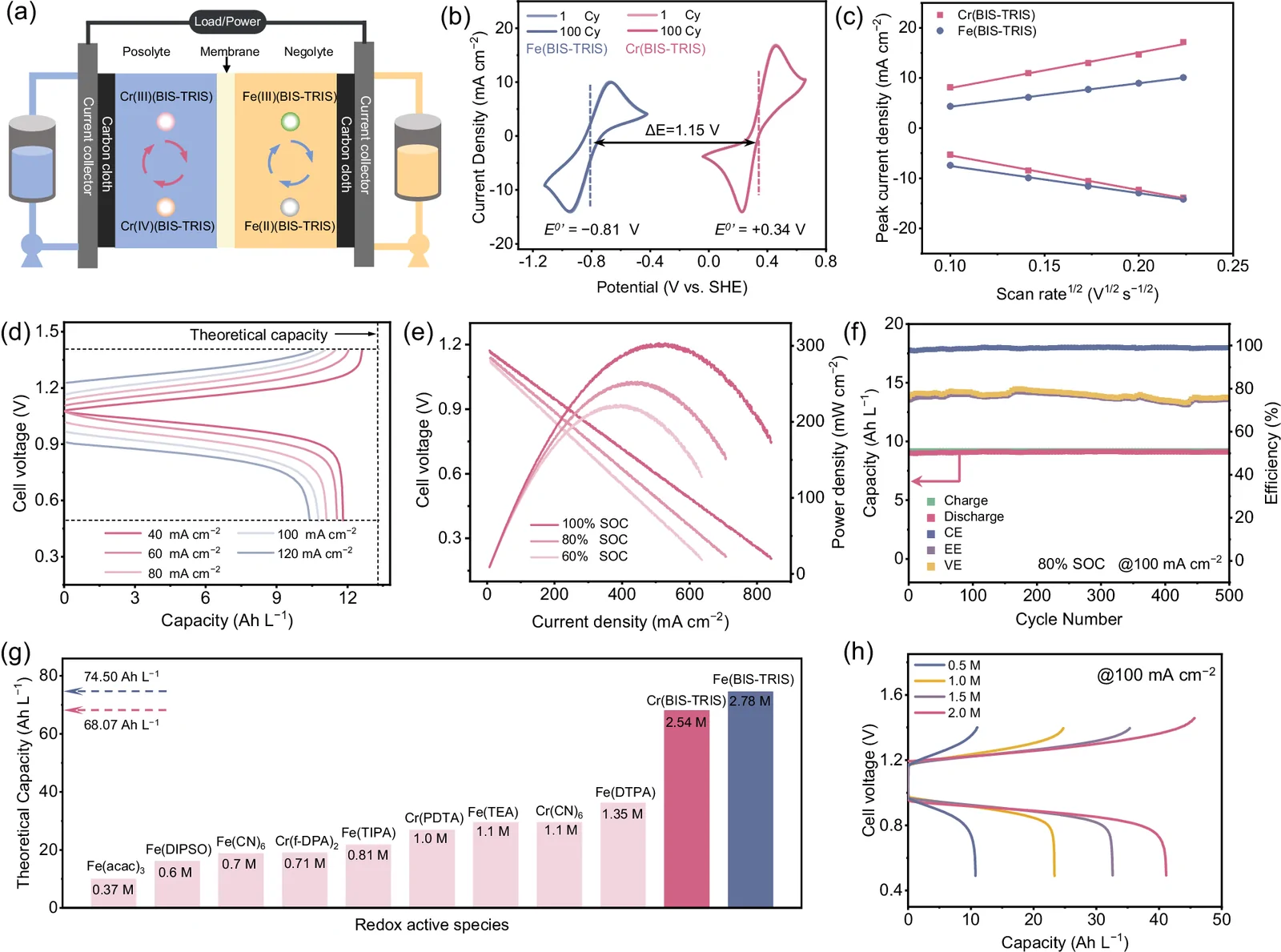
Electrochemical performance and scalability of the alkaline Fe-Cr redox flow battery (RFB). **a** Schematic diagram of the designed alkaline Fe-Cr RFB. **b** Cyclic voltammetry (CV) curves of 0.1 M Cr(BIS-TRIS) posolyte and 0.1 M Fe(BIS-TRIS) negolyte at 50 mV s^−1^. The potentials were not *iR* corrected. **c** Peak current plotted against the square root of the scan rate for the Cr(BIS-TRIS) and Fe(BIS-TRIS) electrolytes. **d** Galvanostatic charge-discharge curves of the alkaline Fe-Cr RFB recorded at 40, 60, 80, 100, and 120 mA cm^−2^, with five consecutive cycles performed at each current density. **e** The polarization curves and power density curves of the alkaline Fe-Cr RFB at 60%, 80%, and 100% states of charge (SOC). **f** Cycling performance of the alkaline Fe-Cr RFB with 50% excess negolyte at 80% SOC. **g** Solubility and theoretical capacity of various Fe/Cr complexes studied in RFBs. The capacities are expressed per litre of the electrolyte containing the corresponding redox-active species, rather than per total electrolyte volume. The source of the literature data shown in this figure can be found in Supplementary Table 6. **h** Galvanostatic charge/discharge curves of the alkaline Fe-Cr RFB using different concentrations of electrolytes at 100 mA cm^−2^. All electrochemical tests were performed at 25 ± 1 °C. Source data are provided as a Source Data file.

Building on the strong-field chelating ligand BIS-TRIS, we developed an alkaline Fe-Cr RFB with reversed posolyte-negolyte roles, comprising a Cr(III/IV)(BIS-TRIS) posolyte and a Fe(III/II)(BIS-TRIS) negolyte (Fig. 4a). CV analysis revealed distinct redox potentials of +0.34 V vs. SHE for Cr(BIS-TRIS) and −0.81 V vs. SHE for Fe(BIS-TRIS) in alkaline media (Fig. 4b), yielding a theoretical cell voltage of 1.15 V. Fe(BIS-TRIS) has been previously investigated as a negolyte in alkaline all-iron RFBs^49–51^ and exhibits an octahedral hexacoordinate structure (Supplementary Fig. 15)^34^, similar to that of Cr(BIS-TRIS). Owing to the strong ligand coordination and robust hexacoordinate geometry, both Cr(BIS-TRIS) and Fe(BIS-TRIS) chelates exhibit electrochemical reversibility and stability under highly alkaline conditions, as evidenced by long-term CV measurements (Fig. 4b).

The electrochemical performance of Cr(BIS-TRIS) was systematically evaluated to assess its viability as an RFB posolyte. Compared with the conventional Cr(II/III) couple, the Cr(III/IV)(BIS-TRIS) redox pair displays faster kinetics. The CV profile of Cr(III/IV)(BIS-TRIS) displays quasi-reversible and symmetric redox peaks at +0.227 V and +0.458 V vs. SHE, whereas the Cr(II/III) transition in CrCl_3_ exhibits poor reversibility and weak peak currents (Supplementary Fig. 16). Electrochemical impedance spectroscopy (EIS) revealed a sevenfold lower charge-transfer resistance (*R*_*ct*_) for Cr(III/IV)(BIS-TRIS) compared with Cr(II/III) in CrCl_3_, indicating faster electron-transfer kinetics (Supplementary Fig. 17, Supplementary Table 3). From scan rate-dependent CVs, its diffusion coefficients (*D*_*O*_) and standard rate constants (*k*_*O*_) were determined (Fig. 4c, Supplementary Figs. 18, 19). Cr(BIS-TRIS) exhibits *D*_*O*_ of 6.93 × 10^−6^ cm^2^ s^−1^ and *k*_*O*_ of 6.85×10^−3^ cm s^−1^, representing a 1000-fold enhancement over uncatalyzed Cr(II/III) in CrCl_3_ (Supplementary Tables 4,5)^24^. These results highlight its rapid kinetics, positioning Cr(BIS-TRIS) as a candidate posolyte for Fe-Cr RFBs. For comparison, Fe(BIS-TRIS) delivers *D*_*O*_ of 3.03 × 10^−6^ cm^2^ s^−1^ and *k*_*O*_ of 2.79 × 10^−3^ cm s^−1^, confirming its suitability as an efficient negolyte.

The full-cell performance of the alkaline Fe-Cr RFB was subsequently evaluated. Galvanostatic charge-discharge cycling at current densities from 40 to 120 mA cm^−2^ showed that the Coulombic efficiency (CE) increased from 93.6% to 98.5%, while the energy efficiency (EE) decreased from 85.1% to 69.6% (Fig. 4d, Supplementary Fig. 20). The decline in EE primarily arises from concentration and Ohmic polarization at higher current densities, whereas the CE trend will be discussed later. The polarization curve at 60% SOC yielded an area-specific resistance (ASR) of 1.46 Ω cm^2^ (Supplementary Fig. 21a), while the high-frequency intercept of the Nyquist plot indicated a total Ohmic resistance of 1.24 Ω cm^2^ (Supplementary Fig. 21b), corresponding to approximately 85% of the polarization ASR. This large Ohmic contribution indicates that additional polarization associated with charge-transfer kinetics and electrolyte mass transport was relatively small under the tested conditions^52,53^. Further validation showed that the alkaline Fe-Cr RFB achieved peak power densities of 248.8 mW cm^−2^ at 80% SOC and 303.6 mW cm^−2^ at 100% SOC (Fig. 4e). To evaluate the cycling stability of the Cr(BIS-TRIS) posolyte, a cell containing 10 mL of 0.5 M Cr(BIS-TRIS) posolyte and 15 mL of 0.5 M Fe(BIS-TRIS) negolyte, corresponding to a 50% volumetric excess of negolyte, was cycled at 100 mA cm^−2^ and 80% SOC. The results show that the alkaline Fe-Cr RFB operates stably over 500 cycles, with no observable capacity decay, while maintaining a coulombic efficiency of 99.0% and an energy efficiency of 75.8% (Fig. 4f). CV and UV-Vis measurements of the posolyte after cycling provide additional evidence for the stability of the Cr(BIS-TRIS) coordination structure (Supplementary Fig. 22). Furthermore, owing to the use of the same ligand in both electrolytes, the negolyte and posolyte of the alkaline Fe-Cr RFB can be prepared as a mixed solution, as is commonly practiced in acidic Fe-Cr systems. The resulting mixed-electrolyte system exhibits competitive performance, delivering a CE of 99.0%, an EE of 72.9%, and stable cycling over 200 cycles (Supplementary Fig. 23).

Battery capacity, largely dictated by the solubility of active species, represents another critical factor for RFB commercialization. The solubilities of Cr(BIS-TRIS) and Fe(BIS-TRIS) were quantified via UV-Vis analysis (Supplementary Figs. 24, 25). As shown in Fig. 4g, the Cr(BIS-TRIS) and Fe(BIS-TRIS) chelates exhibit solubilities of 2.54 M and 2.78 M, respectively, in mixed-cation electrolytes containing 5 M KOH and 5 M NaOH. These concentrations correspond to theoretical capacities of 68.07 Ah L^−1^ based on the Cr(BIS-TRIS) posolyte volume and 74.50 Ah L^−1^ based on the Fe(BIS-TRIS) negolyte volume. To evaluate scalability, full cells employing electrolyte concentrations from 0.5 to 2.0 mol L^−1^ were tested (Fig. 4h). Increasing the concentration reduced CE from 97.5% to 90.1% (the reduction of CE will be discussed later) and EE from 73.7% to 64.3% (Supplementary Fig. 26), primarily due to higher viscosity and diffusion limitations. Nevertheless, the discharge capacity increased from 11.50 to 41.15 Ah L^−1^, increasing the volumetric capacity of the system. Finally, the economic feasibility of the alkaline Fe-Cr RFB was assessed. Benefiting from the low cost of both BIS-TRIS and the earth-abundant Fe and Cr salts, the alkaline Fe-Cr RFB achieves a projected raw material cost of only $17.02 kWh^−1^ (Supplementary Table 7), suggesting potential cost competitiveness relative to commercial VRFBs^54^.

### Capacity recoverability of the alkaline Fe-Cr RFB

**Fig. 5:**
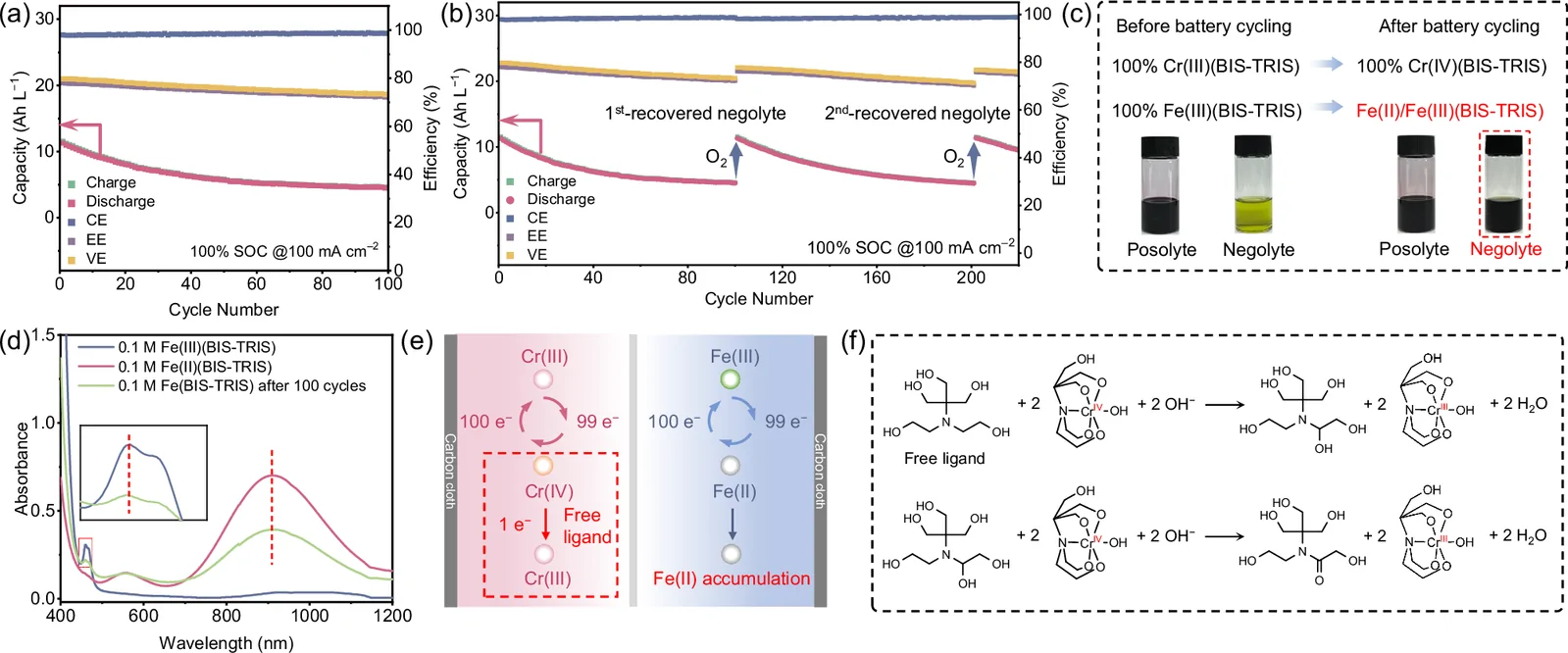
Capacity-recovery mechanism of the alkaline Fe-Cr redox flow battery (RFB). **a** Cycling performance of the 0.5 M alkaline Fe-Cr RFB at 100% state of charge (SOC) and 100 mA cm^−2^. **b** Capacity recovery of the alkaline Fe-Cr RFB through exposure of the circulating negolyte to ambient air for 6 h after completion of the 100th and 200th discharge steps (0% SOC). The capacity-balanced cell was cycled to an upper limit of 100% SOC at 100 mA cm^−2^. **c** Optical images and corresponding electrolyte states before battery cycling and after 100 cycles. The electrolyte samples after cycling were collected at the final discharge state (0% SOC). **d** Ultraviolet-visible (UV-Vis) spectra of Fe(II)(BIS-TRIS), Fe(III)(BIS-TRIS), and the negolyte collected after completion of the 100th discharge step (0% SOC) at 100 mA cm^−2^. **e** Proposed capacity-attenuation mechanism and (**f**) possible oxidation reactions of free BIS-TRIS. All electrochemical tests were performed at 25 ± 1 °C. Source data are provided as a Source Data file.

To evaluate capacity recovery and investigate potential degradation pathways, we employed a capacity-balanced cell configuration with equal electrolyte volumes and cycled the cell to 100% SOC. Under these conditions, gradual capacity decay was observed, with an average CE of 98.3% (Fig. 5a). Notably, when the retained capacity decreased to approximately 40% after the 100th and 200th cycles, it was fully restored by exposing the circulating negolyte to ambient air for 6 h (Fig. 5b). This recovery confirms that the observed capacity loss results from a charge imbalance between the negolyte and posolyte, rather than from irreversible decomposition of the redox-active chelates.

To elucidate the origin of the reversible capacity fade, we analyzed the post-cycling electrolytes. We found that the Fe(III)(BIS-TRIS) negolyte gradually changed from light green to dark black after cycling, resembling the reduced Fe(II)(BIS-TRIS) state (Fig. 5c, Supplementary Fig. 27). After 6 h of exposure to air, the solution returned to its light-green color, confirming oxidative regeneration by atmospheric oxygen. This observation suggests significant accumulation of Fe(II)(BIS-TRIS) during cycling, which is further verified by UV-Vis analysis (Fig. 5d). Quantitative analysis revealed an Fe(II)(BIS-TRIS) concentration of 0.296 M in the post-cycling negolyte, corresponding to the conversion of 59.2% of Fe(III)(BIS-TRIS) to Fe(II)(BIS-TRIS), consistent with the observed 62.5% capacity loss (Supplementary Fig. 28). In contrast, the UV-Vis spectra of both Cr(BIS-TRIS) and Fe(BIS-TRIS) electrolytes before and after cycling (post-air exposure) remained unchanged (Supplementary Figs. 22, 29), confirming the absence of metal-chelate decomposition and intrinsic durability of the chelates.

Further analysis revealed that the CE is highly sensitive to the concentration of free ligand in the posolyte. As the ligand-to-metal ratio increased from 1.1:1 to 1.2:1 and 1.3:1, the CE gradually decreased (Supplementary Fig. 30), indicating that uncoordinated BIS-TRIS molecules likely participate in parasitic side reactions with oxidized Cr species. Therefore, we attribute the reversible capacity fading to a mild side reaction between Cr(IV)(BIS-TRIS) and free BIS-TRIS ligands (Fig. 5e). During charging, a small portion of Cr(IV)(BIS-TRIS) is reduced to Cr(III)(BIS-TRIS) by free ligands, driving the accumulation of Fe(II)(BIS-TRIS) in the negolyte and leading to a gradual charge imbalance between the two electrolytes (Supplementary Fig. 31). The proposed oxidation pathways of BIS-TRIS are illustrated in Fig. 5f, with the corresponding products confirmed by ^1^H nuclear magnetic resonance (NMR) spectroscopy and ESI-HRMS (Supplementary Figs. 32, 33). This side reaction becomes slightly more pronounced at lower current densities or higher electrolyte concentrations due to extended charge-discharge durations (Supplementary Figs. 20, 26). Nevertheless, because this process induces only a reversible charge imbalance without any irreversible chelate decomposition, the capacity can be fully recovered through simple oxidative rebalancing.

## Discussion

In summary, we developed a hexacoordinate Cr(BIS-TRIS) posolyte that leverages the rarely explored Cr(III/IV) redox transition and demonstrated a posolyte-negolyte reversed alkaline Fe-Cr RFB. The Cr(BIS-TRIS) chelate exhibits a redox potential of +0.34 V vs. SHE, an electron-transfer rate constant of 6.85 × 10^−3^ cm s^−1^, and a solubility of 2.54 M, corresponding to a theoretical capacity of 68.07 Ah L^−1^ based on the posolyte volume. When paired with a Fe(BIS-TRIS) negolyte (−0.81 V vs. SHE), the full cell delivers an open-circuit voltage of 1.15 V, achieving a CE of 99.0% and an EE of 75.8% at 80% SOC and 100 mA cm^−2^. Benefiting from the earth-abundant Fe and Cr resources, the projected raw material cost of the electrolyte is as low as $17.02 kWh^−1^, supporting its potential cost competitiveness. Although the system experiences gradual capacity fading during extended cycling, we demonstrate that this capacity loss can be fully recovered through a simple ambient-air exposure treatment of the negolyte. Mechanistic studies reveal that the reversible capacity fade originates from a side reaction between Cr(IV)(BIS-TRIS) and uncoordinated free BIS-TRIS ligands. Therefore, to eliminate the need for periodic capacity regeneration of the alkaline Fe-Cr RFB, future efforts should focus on designing oxidation-resistant ligand frameworks to ensure long-term stability.

## Methods

### Chemicals

Candidate ligands are as follows. (1) Amino-polycarboxylic ligands: Nitrilotriacetic acid (NTA, 97%), 1,3-propylenediaminetetraacetic acid (PDTA, 98%), Diethylenetriaminepentaacetic acid (DTPA, 99%), Ethylenediaminetetraacetic acid (EDTA, 99.5%), trans-1,2-Diaminocyclohexane-N,N,N’,N’-tetraacetic acid (CyDTA, 99%), N-(2-hydroxyethyl)ethylenediamine-N,N’,N’-triacetic acid (HEDTA, 98%), Triethylenetetraminehexaacetic acid (TTHA, 98%), N,N’-1,2-ethanediylbis-1-Aspartic acid (EDDS, 98%), Iminodiacetic acid (IDA, 98%), Methyliminodiacetic acid (MIDA, 98%). (2) Amino-polyphosphonate ligands: Aminomethylphosphonic acid (AMPA, 99%), Iminodi(methylenephosphonic acid) (IDMP, 98%), Nitrilotrimethylene triphosphonic acid (ATMP, 50% in water), Ethylenediamine tetra(methylenephosphonic acid) (EDTMPA, 95%), Diethylenetriamine penta(methylenephosphonic acid) (DTPMPA, 50% in water), Hexamethylenediamine tetra(methylenephosphonic acid) (HDTMPA, 97%), Bis(hexamethylene)triamine penta(methylenephosphonic acid) (BHMTPMP, 50%), Glyphosine (97%), Glycinamide-N,N’-bis(methylenephosphonic acid) (GABMP, 95%), (Aminomethylene)bisphosphonic acid (AMP, 95%). (3) Amino-polyhydroxy ligands: 2-Amino-1,3-propanediol (98%), 3-Amino-1,2-propanediol (97%), Tris(Hydroxymethyl)Aminomethane (Tris, 99.8%), 3-[bis(2-hydroxyethyl)amino]-2-hydroxypropanesulphonic acid (DIPSO, 98%), Triisopropanolamine (TIPA, 95%), N,N-Bis(2-Hydroxyethyl)Isopropanolamine (mTEA, 93%), N-[tris(hydroxymethyl)methyl]-3-amino-2-hydroxypropanesulfonic acid (TAPSO, 99%), Diethanolamine (DEA, 99%), Triethanolamine (TEA, 99%), 2,2-Bis(hydroxymethyl)−2,2’,2”-nitrilotriethanol (BIS-TRIS, 98%).

Iron chloride hexahydrate (FeCl_3_·6H_2_O, 99%), chromium chloride hexahydrate (CrCl_3_·6H_2_O, 98%), D_2_O (99%), NTA, PDTA, DTPA, EDTA, HEDTA, TTHA, IDA, MIDA, ATMP, EDTMPA, DTPMPA, Glyphosine, 2-Amino-1,3-propanediol, 3-Amino-1,2-propanediol, Tris, DIPSO, TIPA, TAPSO, DEA, TEA, and BIS-TRIS were obtained from Aladdin Chemistry Co., Ltd. (China). Sodium hydroxide (NaOH, 98%), potassium hydroxide (KOH, 95%), anhydrous ethanol (99.5%), CyDTA, EDDS, AMPA, IDMP, BHMTPMP, GABMP, and mTEA were obtained from Macklin Biochemical Technology Co., Ltd. (China). AMP was obtained from Bide Pharmatech Co., Ltd. (China). Hydrochloric acid (37 wt%) and aqueous ammonia (25 wt%) were obtained from Sinopharm Chemical Reagent Co., Ltd. Nitrogen gas (99.9%) was obtained from Wuhan Xiangyun Industry and Trade Co., Ltd. All reagents were used without further purification.

### Electrolyte synthesis

To prepare 40 mL of 0.5 M Fe(BIS-TRIS) negolyte, 5.40 g of FeCl_3_·6H_2_O was dissolved in 20 mL of deionized water (DIW) and stirred for 30 min. Subsequently, 5.12 g of BIS-TRIS was added, and the mixture was stirred for another 30 min. KOH (3.93 g) and NaOH (2.80 g) were then added, followed by stirring for 24 h. The solution was diluted to a final volume of 40 mL with DIW. The resulting negolyte had a nominal composition of 0.50 M Fe, 0.6 M total BIS-TRIS, 1.75 M KOH, and 1.75 M NaOH, corresponding to a BIS-TRIS-to-Fe molar ratio of 1.2:1. To prepare 40 mL of 0.5 M Cr(BIS-TRIS) posolyte, 5.33 g of CrCl_3_·6H_2_O was dissolved in 20 mL of DIW and stirred for 30 min. Subsequently, 4.60 g of BIS-TRIS was added, and the mixture was stirred for another 30 min. KOH (3.37 g) and NaOH (2.40 g) were then added, followed by stirring for 24 h. The solution was diluted to a final volume of 40 mL with DIW. The resulting posolyte contained nominal concentrations of 0.50 M Cr, 0.55 M total BIS-TRIS, 1.50 M KOH, and 1.50 M NaOH, corresponding to a BIS-TRIS-to-Cr molar ratio of 1.1:1. Other chromium-complex screening solutions were prepared using the same procedure, with the corresponding ligand substituted for BIS-TRIS. To prepare 40 mL of the mixed electrolyte, 2.13 g of CrCl_3_·6H_2_O and 3.24 g of FeCl_3_·6H_2_O were dissolved in 20 mL of DIW and stirred for 30 min. Subsequently, 4.70 g of BIS-TRIS was added, and the mixture was stirred for another 30 min. KOH (6.73 g) was then added, followed by stirring for 24 h. The solution was diluted to a final volume of 40 mL with DIW. The resulting mixed electrolyte contained nominal concentrations of 0.20 M Cr, 0.30 M Fe, 0.55 M total BIS-TRIS, and 3.00 M KOH, corresponding to a BIS-TRIS-to-total-metal molar ratio of 1.1:1.

For the concentration-dependent flow-cell tests shown in Fig. 4h and Supplementary Fig. 26, the higher-concentration electrolytes were prepared following the same sequential dissolution procedure as that used for the 0.5 M electrolytes. Briefly, the required amount of FeCl_3_·6H_2_O or CrCl_3_·6H_2_O was dissolved in deionized water and stirred for 30 min. BIS-TRIS was then added, and the mixture was stirred for another 30 min. KOH and NaOH were subsequently added at equal molar concentrations, followed by stirring for 24 h. The solution was then adjusted to the desired final volume with deionized water. The 1.0, 1.5, and 2.0 M Fe(BIS-TRIS) negolytes contained total BIS-TRIS concentrations of 1.2, 1.8, and 2.4 M, respectively, corresponding to a BIS-TRIS-to-Fe molar ratio of 1.2:1. Their respective KOH and NaOH concentrations were 3.0 and 3.0 M, 4.25 and 4.25 M, and 5.5 and 5.5 M. The 1.0, 1.5, and 2.0 M Cr(BIS-TRIS) posolytes contained total BIS-TRIS concentrations of 1.1, 1.65, and 2.2 M, respectively, corresponding to a BIS-TRIS-to-Cr molar ratio of 1.1:1. Their respective KOH and NaOH concentrations were 2.75 and 2.75 M, 4.0 and 4.0 M, and 5.25 and 5.25 M. For each concentration-dependent flow-cell test, 10 mL of Cr(BIS-TRIS) posolyte and 10 mL of Fe(BIS-TRIS) negolyte with the same nominal metal concentration were used.

To obtain saturated solutions, FeCl_3_·6H_2_O or CrCl_3_·6H_2_O and BIS-TRIS at a molar ratio of 1: 1.1 were added to a solution containing 5 M KOH and 5 M NaOH. The combination of KOH and NaOH was adopted to enhance the solubility of the metal chelates via a diverse ion effect. The metal concentration was gradually increased with a gradient of 0.1 M (1.5 M, 1.6 M, …) until visible hydroxide precipitation was observed. The resulting turbid suspension was then stirred at 25.0 ± 1.0 °C for 24 h and subsequently filtered to obtain a clear saturated solution. For UV-Vis measurements, standard solutions of Fe(BIS-TRIS) and Cr(BIS-TRIS) with known concentrations were prepared to construct calibration curves based on the Beer-Lambert law. The maximum solubility of the metal chelates in the alkaline electrolyte was then determined from the absorbance-concentration relationship.

### Electrochemical measurements

All electrochemical measurements were performed at 25 ± 1 °C without using a climatic or environmental chamber. Three-electrode measurements were conducted under ambient atmosphere. CV measurements were conducted using an EC-Lab potentiostat (BioLogic, VMP-300). A three-electrode configuration was employed with a glassy carbon working electrode (4 mm diameter) and a platinum flake counter electrode. For Cr(BIS-TRIS) and Fe(BIS-TRIS) in alkaline media, an Hg/HgO reference electrode was used. For CrCl_3_ in acidic media, an Ag/AgCl reference electrode was employed. The supporting electrolytes were 5 M KOH for the complex solutions (0.1 M) and 1 M HCl for the CrCl_3_ solution (0.1 M). Three-electrode electrochemical impedance spectroscopy (EIS) measurements were performed using a glassy carbon working electrode with an active area of 0.1256 cm^2^. Before each measurement, the electrode system was allowed to equilibrate at open circuit for 30 min. For full-cell EIS measurements, the cell was charged to 60% SOC at 80 mA cm^−2^ and then allowed to rest at open circuit for 30 min to reach a quasi-stationary state. The electrolytes were circulated at 60 mL min^−1^ during the full-cell EIS measurements. Both three-electrode and full-cell EIS measurements were conducted in potentiostatic mode at the respective open-circuit potential or voltage using a single-sine voltage perturbation with an amplitude of 10 mV. The frequency was swept logarithmically from 1 MHz to 0.01 Hz with 6 data points per decade. The high-frequency real-axis intercept was determined by linear interpolation between the two adjacent impedance data points bracketing *Z”* = 0, where *Z’* and *Z”* represent the real and imaginary components of the impedance, respectively. The two points were (*Z’*, *Z”*) = (0.07703, −0.00194) and (0.07797, 0.000629), yielding an interpolated intercept of 0.07774 Ω. Based on an electrode area of 16 cm^2^, the corresponding area-specific resistance was 1.24 Ω cm^2^. All measurements were performed at 25 ± 1 °C.

### Kinetic parameter calculations

The peak current density can be expressed as^47^:

1 $${I}_{p}=0.227{Fck}\exp \left[\frac{\alpha F}{RT}({E}^{0{\hbox{'}}}-{E}_{p})\right]$$

where *I*_*p*_ is the peak current density (A·cm^−2^), *F* is Faraday’s constant (96,485 C mol^−1^), *c* is the concentration of Cr/Fe ions in the bulk (0.0001 mol·mL^−1^), *k* is the standard rate constant of the reaction (cm·s^−1^), *α* is the transfer coefficient (0.5), *R* is the gas constant (8.314 J K^−1^ mol^−1^), T is the temperature (K). *E*_*p*_ is the peak potential (V), and *E*^*0’*^ is the standard potential (V). In this case, *E*^*0’*^ is calculated as follows:

2 $${E}^{0{{{\hbox{'}}}}}=\frac{{\sum }_{i=1}^{m}\frac{{E}_{p,i}^{A}+{E}_{p,i}^{C}}{2}}{m}$$

### Diffusion-coefficient calculations

The peak current can also be described by the Randles-Ševčík equation:

3 $${I}_{p}=0.4463{nFAC}\sqrt{\frac{{nF}\nu D}{{RT}}}$$

where *I*_*p*_ is the peak current (A), *n* is the number of electrons, *F* is Faraday’s constant (96,485 C mol^−1^), *A* is the electrode area (0.1256 cm^2^ for the glassy carbon electrode), *C* is the bulk concentration of the electrolyte (0.0001 mol mL^−1^), *v* is the scan rate (V s^−1^), *D* is the diffusion coefficient (cm^2^ s^−1^), *R* is the gas constant (8.314 J K^−1^ mol^−1^), and *T* is the temperature (K). The Randles-Ševčík equation is often abbreviated by assuming a temperature of 298 K (25 °C). Thus the equation could be simplified as follows^29^.

4 $${I}_{p}=2.687\times {10}^{5}{n}^{\frac{3}{2}}{{ACD}}^{\frac{1}{2}}{\nu }^{\frac{1}{2}}$$

### Physical characterization

Unless otherwise stated, ex situ electrolyte sampling and sample preparation were conducted at 25 ± 1 °C under ambient atmosphere. At the designated SOC, cell operation was paused, and aliquots were withdrawn directly from the corresponding electrolyte reservoirs and transferred in sealed sample vials for characterization. NMR spectra were recorded using an AscendTM 600 MHz spectrometer. Dried samples were dissolved in D_2_O before ^1^H NMR analysis, and the spectra were processed using MestReNova 14.1. The post-cycling Cr(BIS-TRIS) posolyte used for ^1^H NMR analyses was collected at 100% SOC after 200 charge-discharge cycles at 100 mA cm^−2^ and 25 ± 1 °C. EDS (Hitachi SU8010 field-emission scanning electron microscope) was used to analyse the precipitates obtained from the Cr(PDTA) electrolyte. UV-Vis absorption spectra were recorded using a Shimadzu UV-3600 spectrophotometer. The electrolyte samples were diluted with 1 M KOH before measurement. Samples were collected from the Cr(BIS-TRIS) posolyte at 0%, 20%, 40%, 60%, 80% and 100% SOC during the fifth charge-discharge cycle at 80 mA cm^−2^ and 25 ± 1 °C. For post-cycling CV and UV-Vis measurements, the Cr(BIS-TRIS) posolyte with a ligand-to-metal molar ratio of 1.1:1 was collected at the final discharge state (0% SOC) after 500 charge-discharge cycles at 100 mA cm^−2^ and 25 ± 1 °C. Fourier-transform infrared spectra were recorded using a Nicolet iS50R spectrometer. Raman spectra were recorded using a LabRAM HR800 spectrometer with a 532 nm excitation laser. XRD patterns were collected using an Empyrean diffractometer with Cu Kα radiation over a 2θ range of 10–90° at a scan rate of 5° min^−1^. ESI-HRMS (Thermo Scientific Q Exactive) was used to determine the molecular compositions of the chromium chelates. The spectra were acquired in positive-ion mode. The post-cycling Cr(BIS-TRIS) posolyte was collected at 100% SOC after 200 charge-discharge cycles at 100 mA cm^−2^ and 25 ± 1 °C. Before measurement, the sample was diluted tenfold with 0.1% aqueous ammonia. EPR (EMXmicro-6/1/P/L) spectroscopy was used to examine the oxidation state of the chromium chelates. Samples were collected from the Cr(BIS-TRIS) posolyte at 0% and 100% SOC during the fifth charge-discharge cycle at 80 mA cm^−2^ and 25 ± 1 °C. XPS (AXIS-ULTRA DLD-600W) was used to analyse the oxidation states of the chromium chelates. Samples were collected from the 0.5 M Cr(BIS-TRIS) posolyte after discharging to 0% SOC or charging to 100% SOC at 80 mA cm^−2^ and 25 ± 1 °C. Ten volumes of ethanol were added to each electrolyte sample to precipitate the chromium complexes, and the resulting precipitates were used for XPS analysis. Cr K-edge XAS spectra were collected in transmission mode using a commercial laboratory-based XAS spectrometer (Table XAFS-500A, Specreation Instruments Co., Ltd.). An X-ray tube operated at 20 kV and 15 mA was used as the X-ray source. Si(511) spherically bent crystal analyzers with a radius of curvature of 500 mm, arranged in a 250 mm Rowland-circle geometry, were used to provide a monochromated X-ray beam.

### Theoretical computation methods

DFT calculations were conducted using the Gaussian 16 software^55^. The Becke three-parameter hybrid exchange-correlation functional (B3LYP) with the 6-311 G basis set was employed for geometry optimization and electronic-structure calculations^56,57^. Further refinement of single point energies for all stationary points was carried out with this method. We used Grimme’s D3 dispersion correction to address van der Waals interactions. For geometry optimization, the parameters were set to achieve forces below 10^−5^ hartree/bohr and energy changes less than 10^−6^ hartree. Frontier molecular orbital (FMO) analysis was carried out using the Multiwfn^58^ software package and visualized the results using visual molecular dynamics (VMD)^59^. A limitation of this approach is that the simplified molecular models do not fully capture the concentrated alkaline electrolyte environment, including dynamic solvation, ion pairing, coordination equilibria and electrode-interface effects; therefore, the calculations are intended to provide qualitative molecular-level trends rather than quantitative predictions under operating conditions. The optimized Cartesian coordinates are provided separately for Cr(III)(BIS-TRIS) (Supplementary Data 1), Cr(IV)(BIS-TRIS) (Supplementary Data 2), Cr(III)(TEA) (Supplementary Data 3), Cr(IV)(TEA) (Supplementary Data 4), Cr(III)(H_2_O)_6_ (Supplementary Data 5), Cr(IV)(H_2_O)_6_ (Supplementary Data 6), Cr(III)(DEA) (Supplementary Data 7), and Cr(IV)(DEA) (Supplementary Data 8).

### Calculation of raw material cost of the electrolyte

The price of raw materials was obtained from Guidechem.com (https://china.guidechem.com/) and listed in Supplementary Table 7. The projected raw-material cost of the electrolyte included the positive and negative redox-active materials and supporting electrolytes, reflecting the material requirements of a large-scale system. The electrolyte cost was normalized to the theoretical energy density to facilitate comparison with previously reported systems and was calculated as follows^47^.

5 $${C}_{e}=\frac{{\sum }_{x}{c}_{x}{M}_{x}{P}_{x}}{{ncFV}}$$

where *C*_e_ is the projected raw-material cost of the electrolyte ($ kWh^−1^); *c*_x_ is the concentration of component *x* (mol L^−1^); *M*_x_ is its molar mass (g mol^−1^); *P*_x_ is its raw-material price ($ kg^−1^); *n* is the number of electrons transferred per molecule; *c* is the concentration of the capacity-limiting active species (mol L^−1^); *F* is Faraday’s constant (26.8 Ah mol^−1^); and *V* is the cell voltage (V). Using these parameters, the projected raw-material cost of the 0.5 M Fe(BIS-TRIS)/Cr(BIS-TRIS) redox flow battery is $17.02 kWh^−1^.

### Flow battery performance

Cell tests were conducted using a homemade flow cell equipped with two EDP-08 carbon cloth electrodes (Jingzhou Hot-Material Co., Ltd.; thickness, 1 mm; carbon content, 99%; geometric area, 16 cm^2^), graphite flow-field plates serving as current collectors (Beijing Jinglong Special Carbon Technology Co., Ltd.; thickness, 5 mm), and a dense N-212 perfluorosulfonic acid ion-exchange membrane (ThinkerGroup; thickness, 50 μm; lateral dimensions, 4.0 cm × 4.0 cm). Before use, the membrane was soaked in 1.5 M KOH at 80 °C for 2 h. The carbon cloth electrodes were used as received without further pretreatment. The electrolytes were continuously circulated through the cell at 60 mL min^−1^ using a two-channel peristaltic pump (Zibo Newking Electromechanical Equipment, China). No cells were tested under static conditions.

Three cell configurations were used. For the capacity-balanced configuration, 10 mL of 0.5 M Cr(BIS-TRIS) posolyte and 10 mL of 0.5 M Fe(BIS-TRIS) negolyte were used. For the configuration with 50% excess negolyte, used for the long-term cycling experiment in Fig. 4f, 10 mL of 0.5 M Cr(BIS-TRIS) posolyte and 15 mL of 0.5 M Fe(BIS-TRIS) negolyte were used. For the posolyte-capacity-limiting configuration used in Fig. 3b and Supplementary Fig. 13, 10 mL of 0.5 M Cr(BIS-TRIS) posolyte and 30 mL of 0.5 M Fe(BIS-TRIS) negolyte were used. Unless otherwise specified, full-cell experiments were conducted using the capacity-balanced configuration.

All electrochemical measurements were performed using a Neware battery testing system (CT-4008T) at 25.0 ± 1.0 °C. Before initial cycling, the negolyte was purged with 99.9% nitrogen for 10 min to remove dissolved oxygen. Flow-cell cycling was conducted galvanostatically between cell-voltage limits of 0.5 and 1.4 V. Unless otherwise specified for SOC-controlled measurements, each charging step was terminated when the cell voltage reached 1.4 V, and each discharging step was terminated when the voltage reached 0.5 V. For SOC calibration, the cell was initially cycled at 80 mA cm^−2^ under the same voltage limits. After the capacity had stabilized, the discharge capacity obtained in the fifth cycle, *Q*_ref_, was used as the reference capacity. During charging, the SOC was calculated as SOC = *Q*_ch_*/Q*_ref_ × 100%, where *Q*_ch_ is the cumulative charge capacity measured from the fully discharged state. During discharging, the SOC was calculated as SOC = (*Q*_ref_ – *Q*_dis_)*/Q*_ref_ × 100%, where *Q*_dis_ is the cumulative discharge capacity measured from the fully charged state.

For the capacity-recovery experiment shown in Fig. 5b, recovery treatments were conducted after the 100th and 200th cycles. After completion of the corresponding discharge step, cycling was paused and no current was applied. The negolyte reservoir was opened to ambient laboratory air while the electrolyte continued to circulate through the assembled flow cell at 60 mL min^−1^. The treatment was maintained for 6 h at atmospheric pressure and 25.0 ± 1.0 °C. This treatment oxidized the accumulated Fe(II)(BIS-TRIS) species to Fe(III)(BIS-TRIS). After air exposure, the negolyte was purged with 99.9% nitrogen for 10 min to remove residual dissolved oxygen. Cycling was then resumed at 100 mA cm^−2^ using the same capacity-balanced configuration, electrolyte flow rate, SOC limit and discharge-voltage limit.

## Supplementary information

Supplementary Information

Description of Additional Supplementary Files

Supplementary Data 1-8

Transparent Peer Review file

## Source data

Source Data

## Data Availability

The data generated in this study are provided in the Supplementary Information and Source Data file. Supplementary Data 1–8, containing the optimized Cartesian coordinates of the DFT models, are provided with this paper. Source data are provided with this paper.
