# Supplementary material for: Ligand engineering enables an alkaline iron-chromium redox flow battery
Source: Nat Commun. 2026 Sep 30;17:11240. doi: 10.1038/s41467-026-77810-8 (PMC13720190; doi:10.1038/s41467-026-77810-8)
Supplement: Supplementary file 1 [file 41467_2026_77810_MOESM1_ESM.pdf]

Supplementary Information

Ligand engineering enables an alkaline iron-chromium redox flow battery

Wendong Yang [1], Pei Liu [1,2,], Linfeng Wang [1], Hua Jiang [1], Shuangbin Zhang [3], Bin Luo [3], Xue Long [1], Yilin Zeng [1], Xuan Cai [1], Yifan Zhang [1], Zuoxuan Gan [1], Huaiyu Ke [1], Jinhua Guo [1,*], and Jiangjiang Duan [1,*]*

[1] Wuhan National Laboratory for Optoelectronics, School of Optical and Electronic Information, Huazhong University of Science and Technology, Wuhan, 430074, China

[2] State Key Laboratory of Intelligent Manufacturing Equipment and Technology, School of Mechanical Science and Engineering, Huazhong University of Science and Technology, Wuhan 430074, China

[3] Australian Institute for Bioengineering and Nanotechnology (AIBN) & School of Chemical Engineering, The University of Queensland, Brisbane, QLD, 4072, Australia

* Correspondence: peiliu@hust.edu.cn; jinhua_joey@126.com; jiangjduan@hust.edu.cn

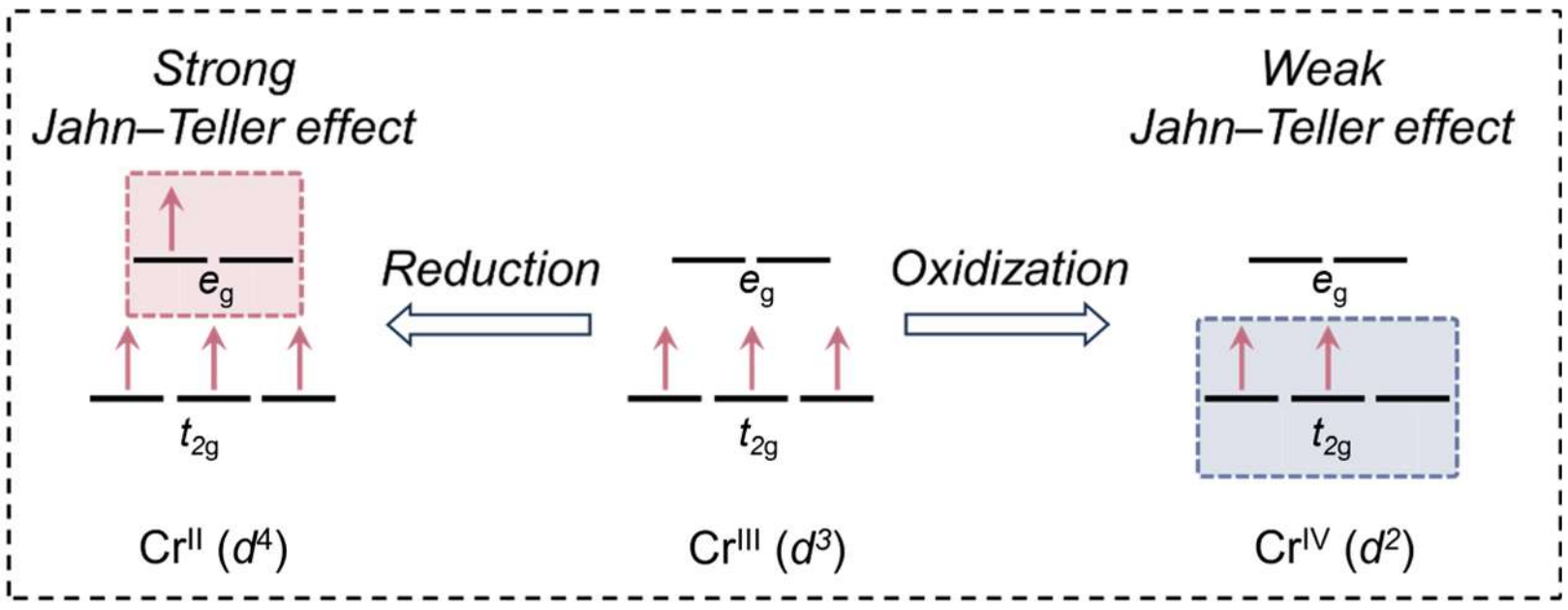

Supplementary Fig. 1 | Jahn-Teller effects of Cr species in different oxidation states.

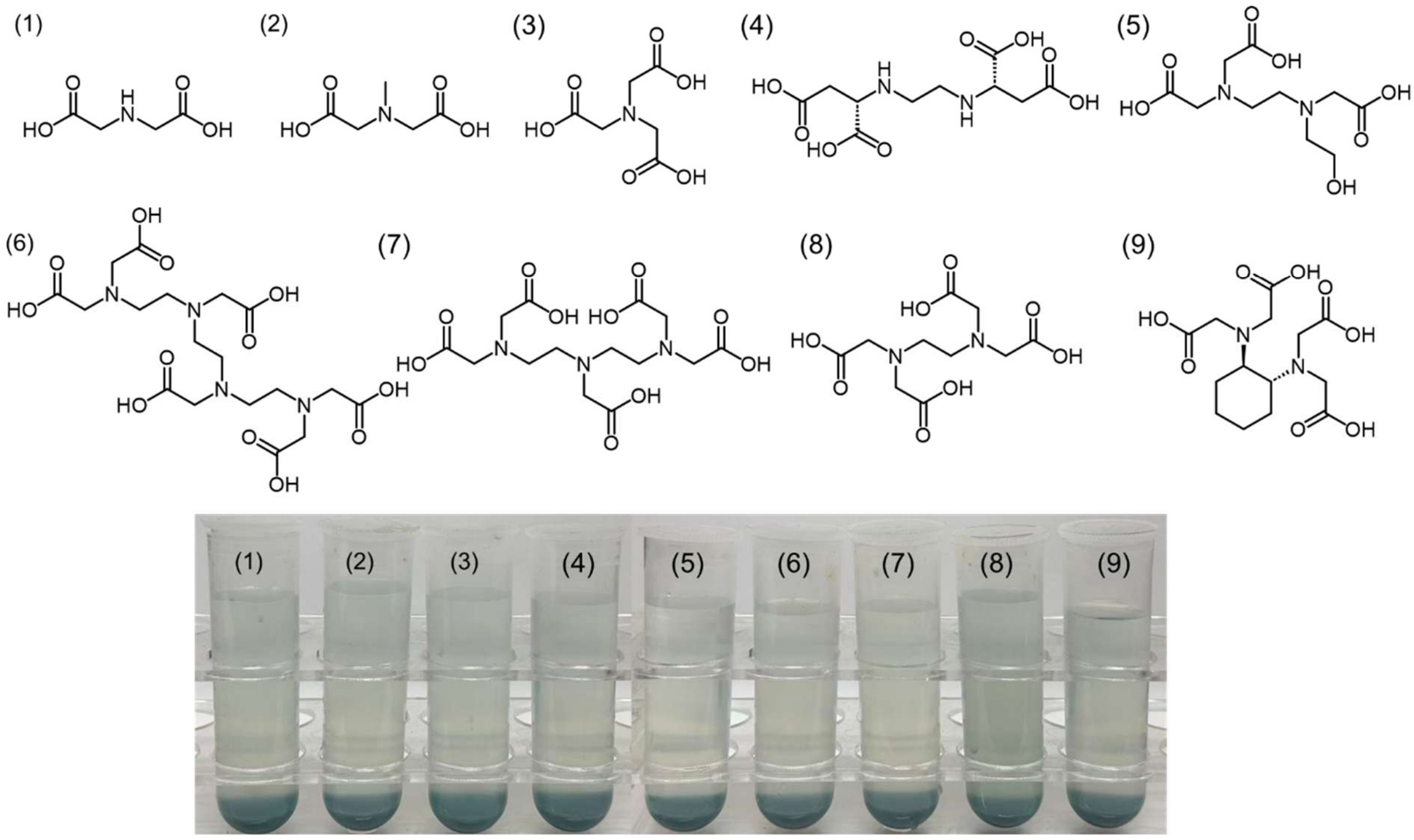

Supplementary Fig. 2 | Cr-amino-polycarboxylic ligand complexes. Molecular structures of amino-polycarboxylic ligands and optical photographs of Cr-amino-polycarboxylic ligand complex solutions in alkaline environments (pH = 14).

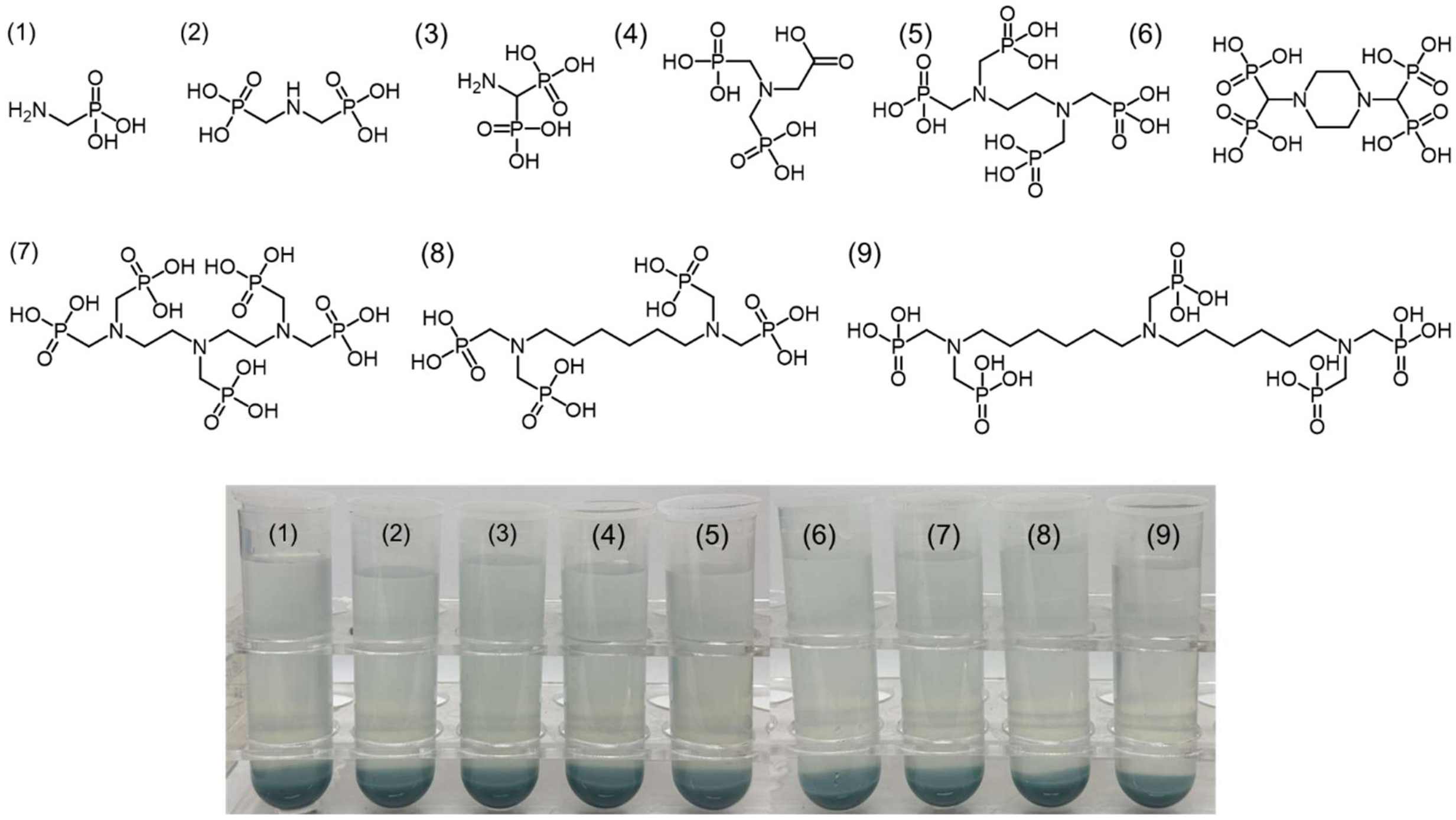

Supplementary Fig. 3 | Cr-amino-polyphosphonate ligand complexes. Molecular structures of amino-polyphosphonate ligands and optical photographs of Cr-amino-polyphosphonate ligand complex solutions in alkaline environments (pH = 14).

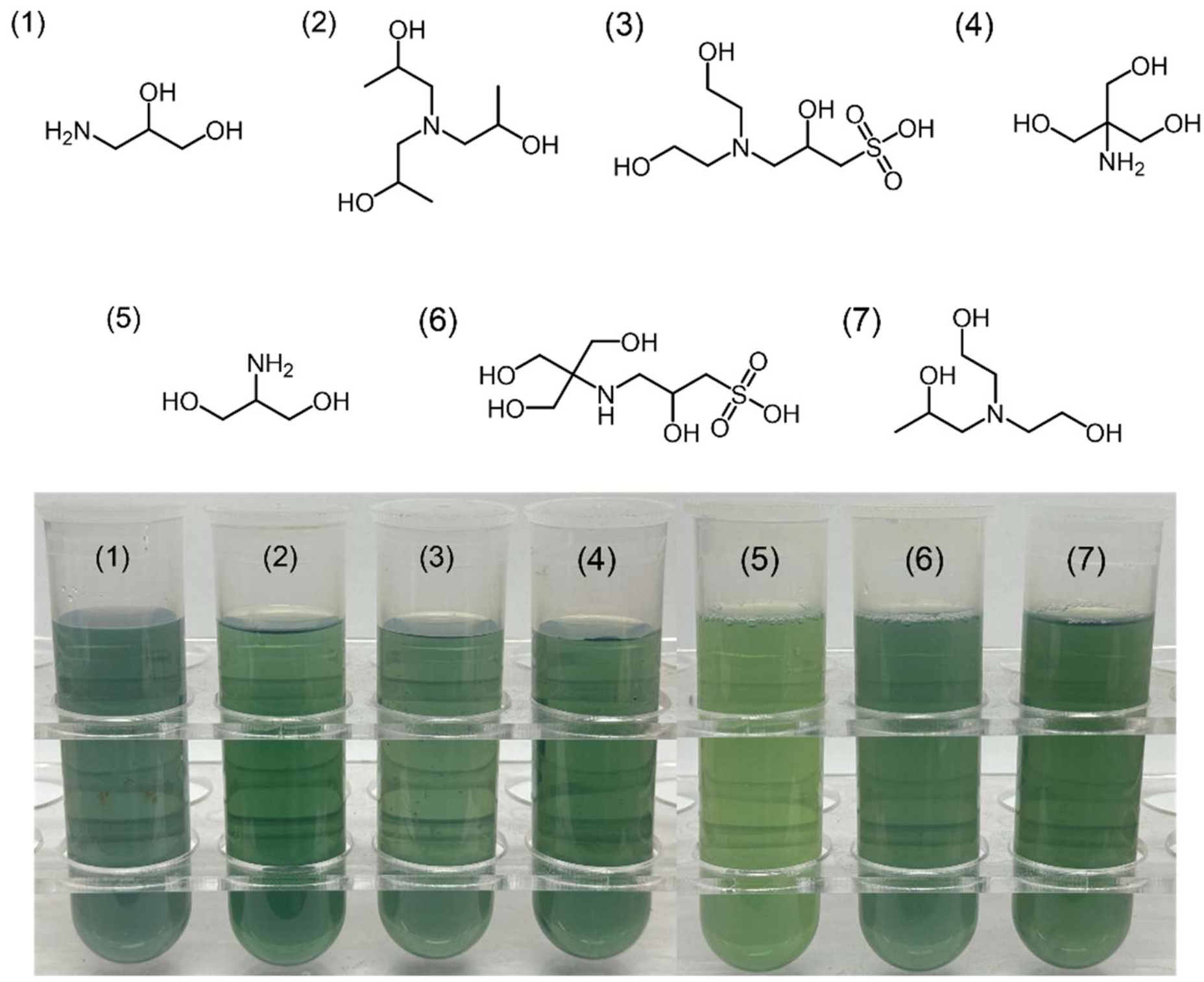

Supplementary Fig. 4 | Cr-amino-polyhydroxy ligand complexes. Molecular structures of amino-polyhydroxy ligands and optical photographs of Cr-amino-polyhydroxy ligand complex solutions in alkaline environments (pH = 14).

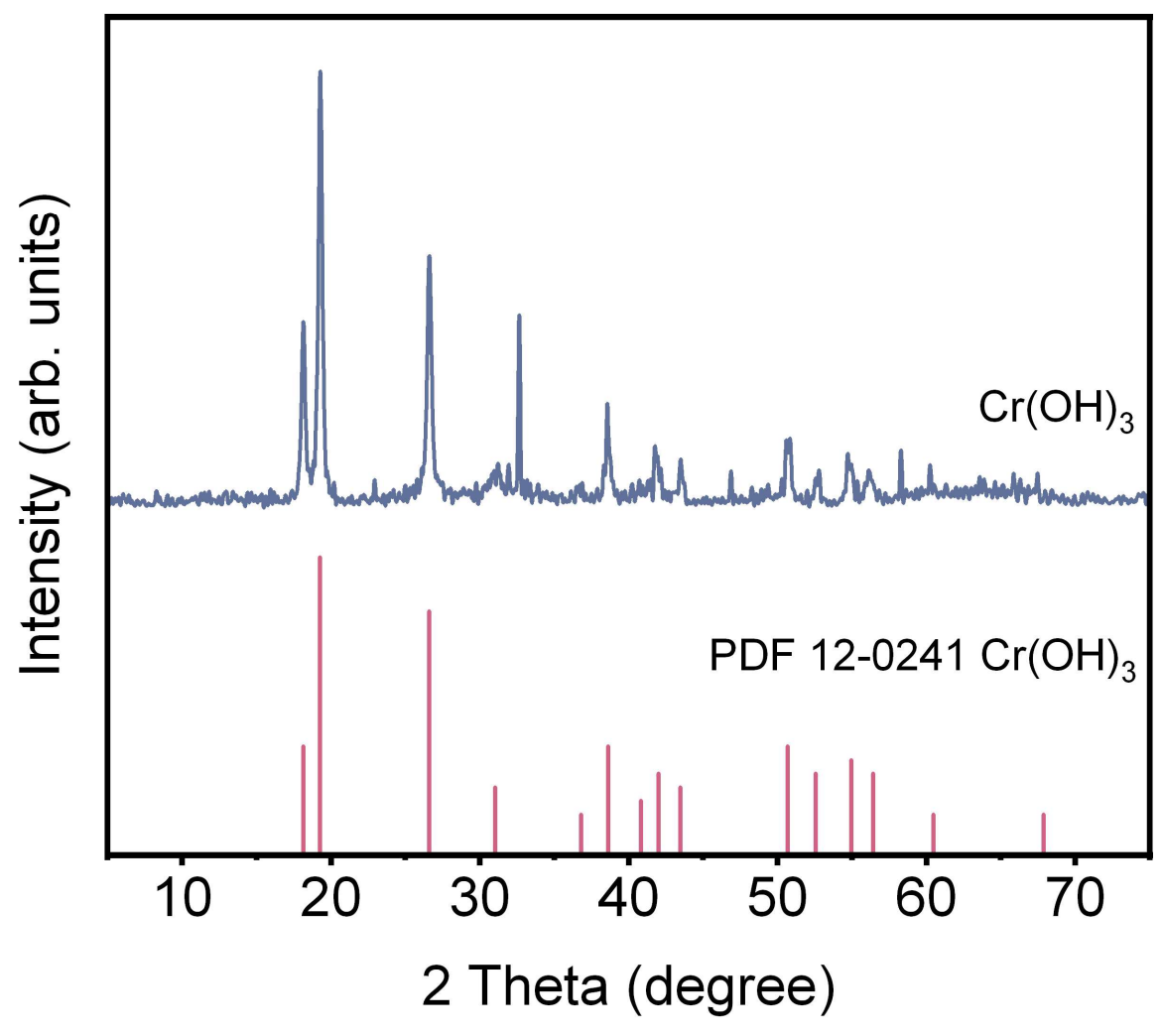

Supplementary Fig. 5 | Phase characterization of precipitates from Cr(PDTA) solution. X-ray diffraction (XRD) patterns of the precipitates resulting from Cr(PDTA) solutions in alkaline environments. Source data are provided as a Source Data file.

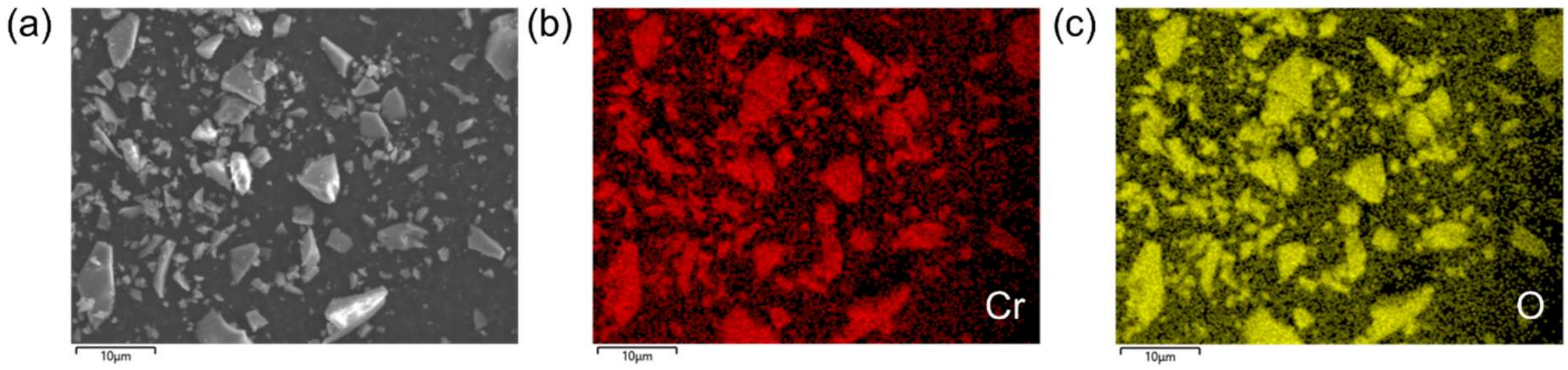

Supplementary Fig. 6 | Morphological and elemental characterization of precipitates from Cr(PDTA) solution. (a) Scanning electron microscopy (SEM) image of the precipitates obtained from Cr(PDTA) solutions under alkaline conditions. (b,c) Corresponding energy-dispersive X-ray spectroscopy (EDS) elemental mappings showing the spatial distributions of Cr (b) and O (c).

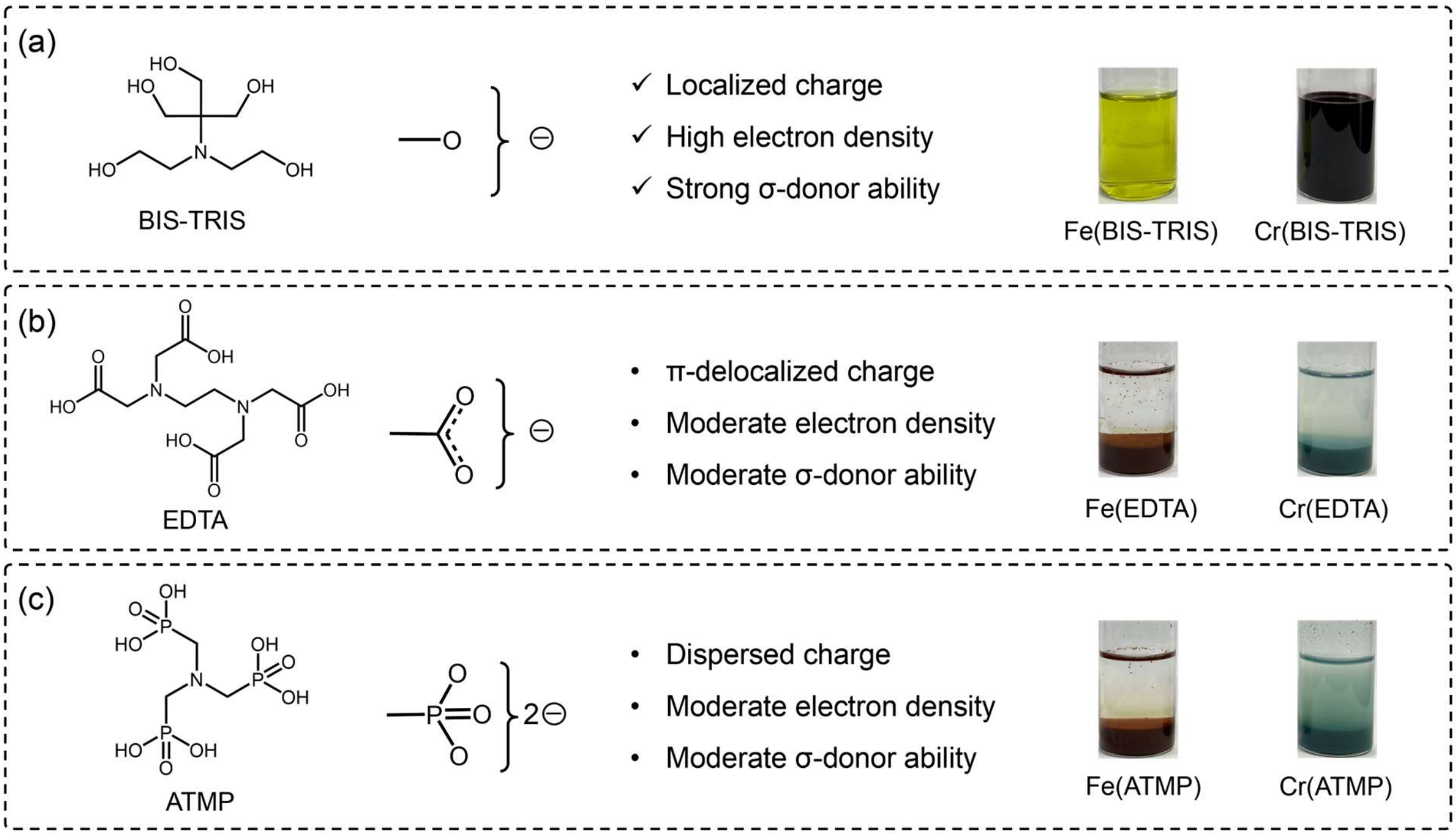

Supplementary Fig. 7 | Comparison of ligand electronic characteristics and electrolyte stability in alkaline systems. Electronic structure of coordinating atoms in (a) an amino-polyhydroxy ligand (BIS-TRIS), (b) an amino-polycarboxylate ligand (EDTA), and (c) an amino-polyphosphonate ligand (ATMP), together with optical images of their corresponding metal chelates in alkaline media (pH = 14).

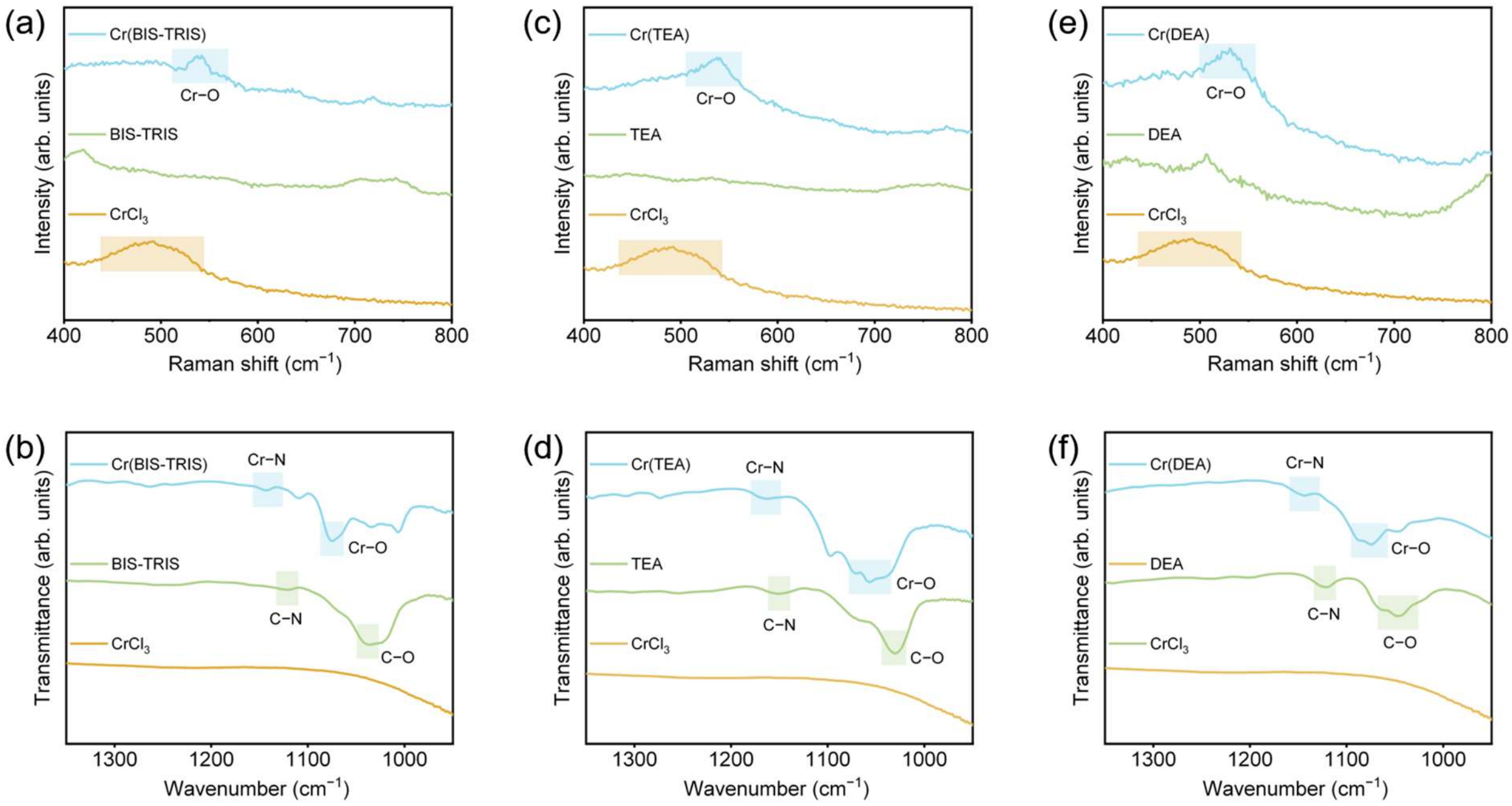

Supplementary Fig. 8 | Vibrational characterization of Cr-amino-polyhydroxy ligand complexes. (a) Raman and (b) Fourier-transform infrared spectroscopy (FTIR) spectra of Cr(BIS-TRIS), BIS-TRIS, and $CrCl_3$ solutions. (c) Raman and (d) FTIR spectra of Cr(TEA), TEA, and $CrCl_3$ solutions. (e) Raman and (f) FTIR spectra of Cr(DEA), DEA, and $CrCl_3$ solutions. Source data are provided as a Source Data file.

Complementary Raman and FTIR analyses also evidenced coordination, with the appearance of a characteristic Cr-O vibration at 542 cm^{-1} in the Raman spectrum[1] and the emergence of new FTIR peaks at 1074 and 1143 cm^{-1} corresponding to Cr-O and Cr-N bonds[2-5]. Similarly, the molecular structures of Cr(DEA) and Cr(TEA) complexes were characterized using the same analytical methods.

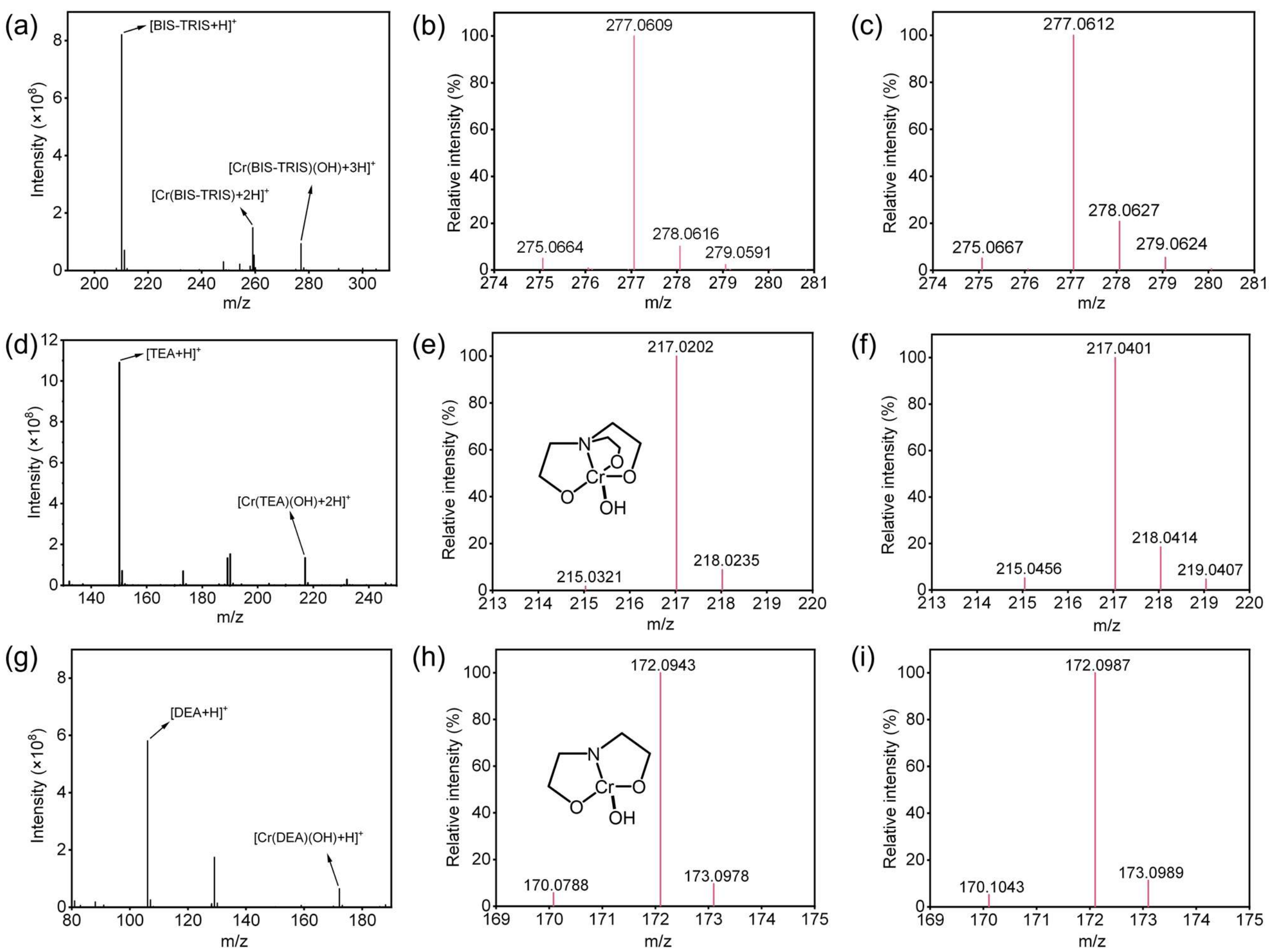

Supplementary Fig. 9 | Electrospray ionization high-resolution mass spectrometry (ESI-HRMS) characterization of Cr-amino-polyhydroxy ligand complexes. (a,b) Experimental ESI-HRMS spectra and (c) simulated spectrum of [Cr(BIS-TRIS)(OH)+3H]$^+$ ($C_8H_{19}CrNO_6^+$, z = +1; observed m/z = 277.0609; calculated m/z = 277.0612). The observed fragment-ion peaks further support this assignment. (d,e) Experimental and (f) simulated ESI-HRMS spectra of [Cr(TEA)(OH)+2H]$^+$ ($C_6H_{15}CrNO_4^+$, z = +1; observed m/z = 217.0202; calculated m/z = 217.0401). (g,h) Experimental and (i) simulated ESI-HRMS spectra of [Cr(DEA)(OH)+H]$^+$ ($C_4H_{11}CrNO_3^+$, z = +1; observed m/z = 172.0943; calculated m/z = 172.0987). Source data are provided as a Source Data file.

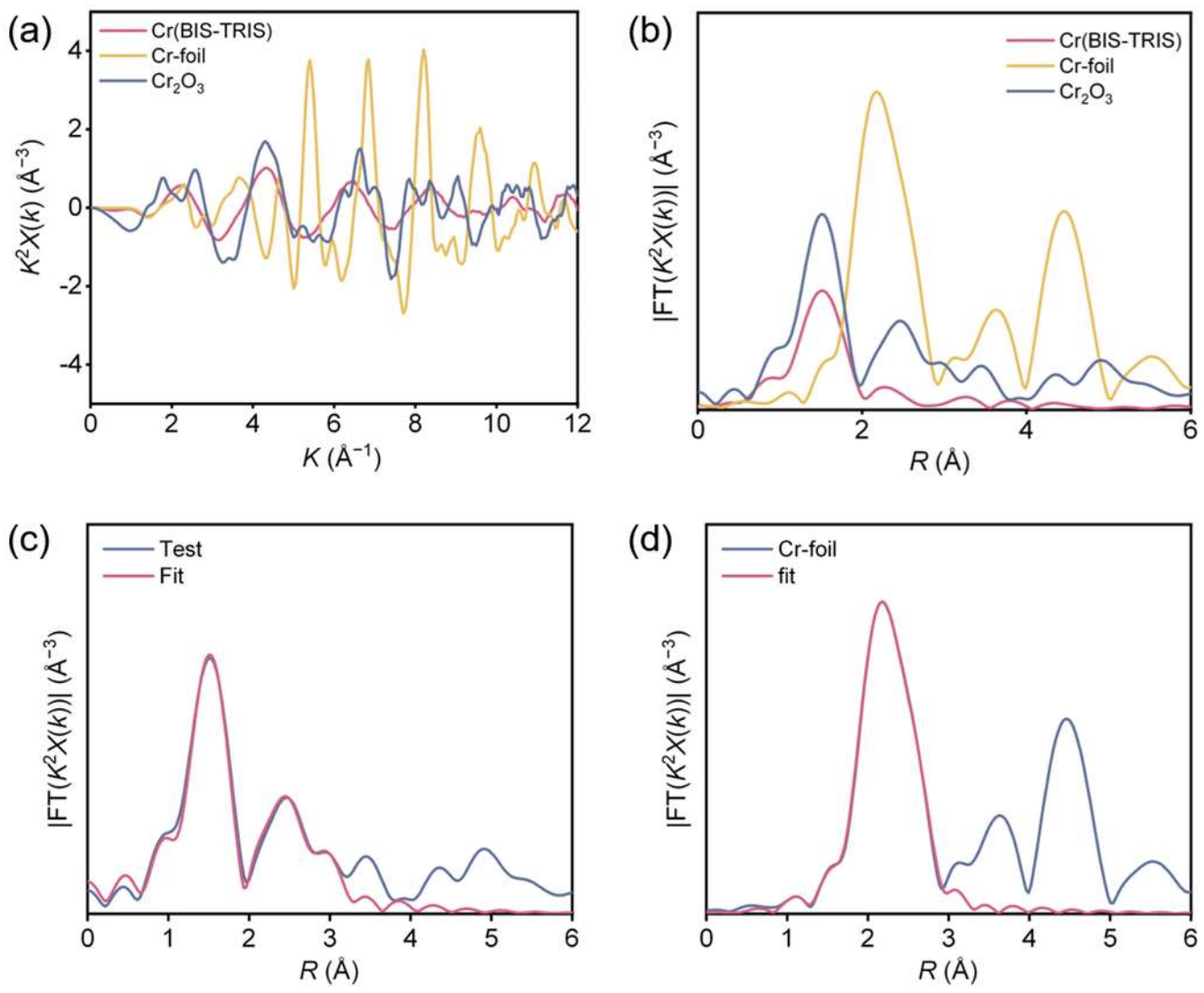

Supplementary Fig. 10 | Cr K-edge extended X-ray absorption fine structure (EXAFS) characterization of Cr(BIS-TRIS) and reference samples. (a) Fourier transforms of the Cr K-edge EXAFS of Cr(BIS-TRIS) and reference samples Cr foil and Cr_2O_3 in K-space. (b) Fourier-transformed experimental EXAFS spectra of Cr(BIS-TRIS), Cr foil, and Cr_2O_3 in R-space. (c, d) Fourier-transformed experimental and fitting EXAFS spectra of (c) Cr_2O_3 and (d) Cr foil in R-space. Source data are provided as a Source Data file.

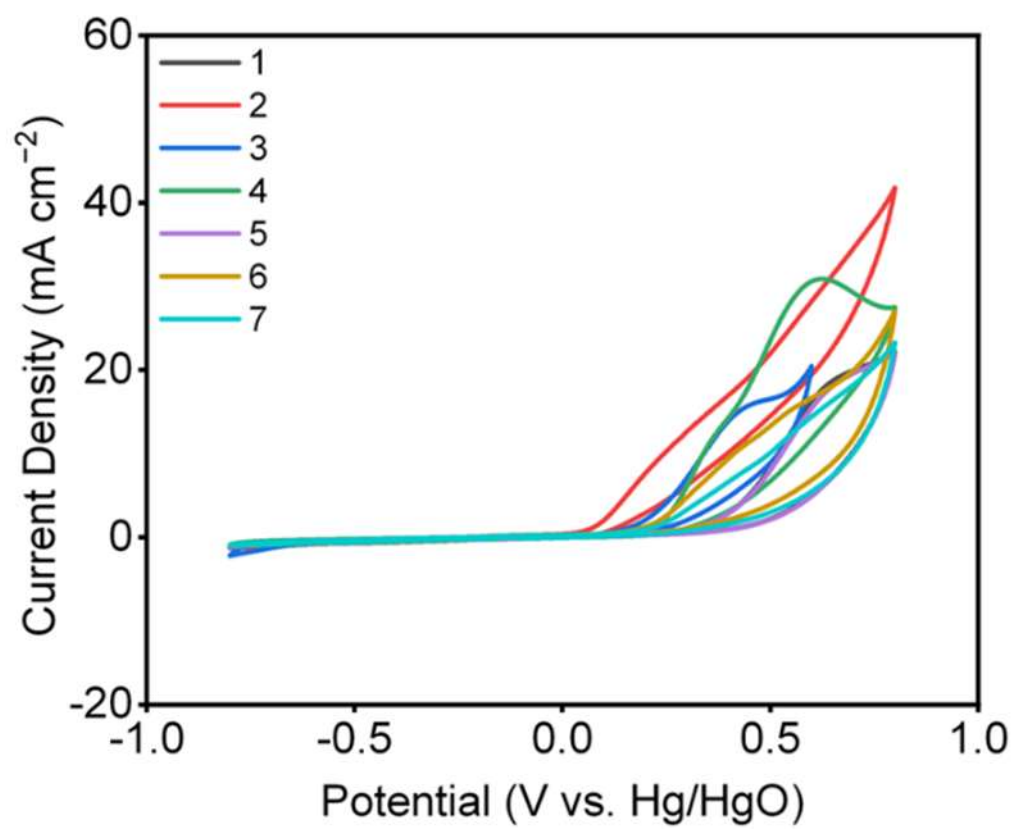

Supplementary Fig. 11 | Electrochemical screening of Cr-amino-polyhydroxy ligand complexes. Cyclic voltammetry (CV) curves of Cr-amino-polyhydroxy ligand complex solutions at 50 mV s^{-1}. Samples 1–7 correspond to the seven ligands shown in **Supplementary Fig. 4**. The measurements were performed using freshly prepared, uncycled electrolyte samples at 25 ± 1 °C without iR correction. Source data are provided as a Source Data file.

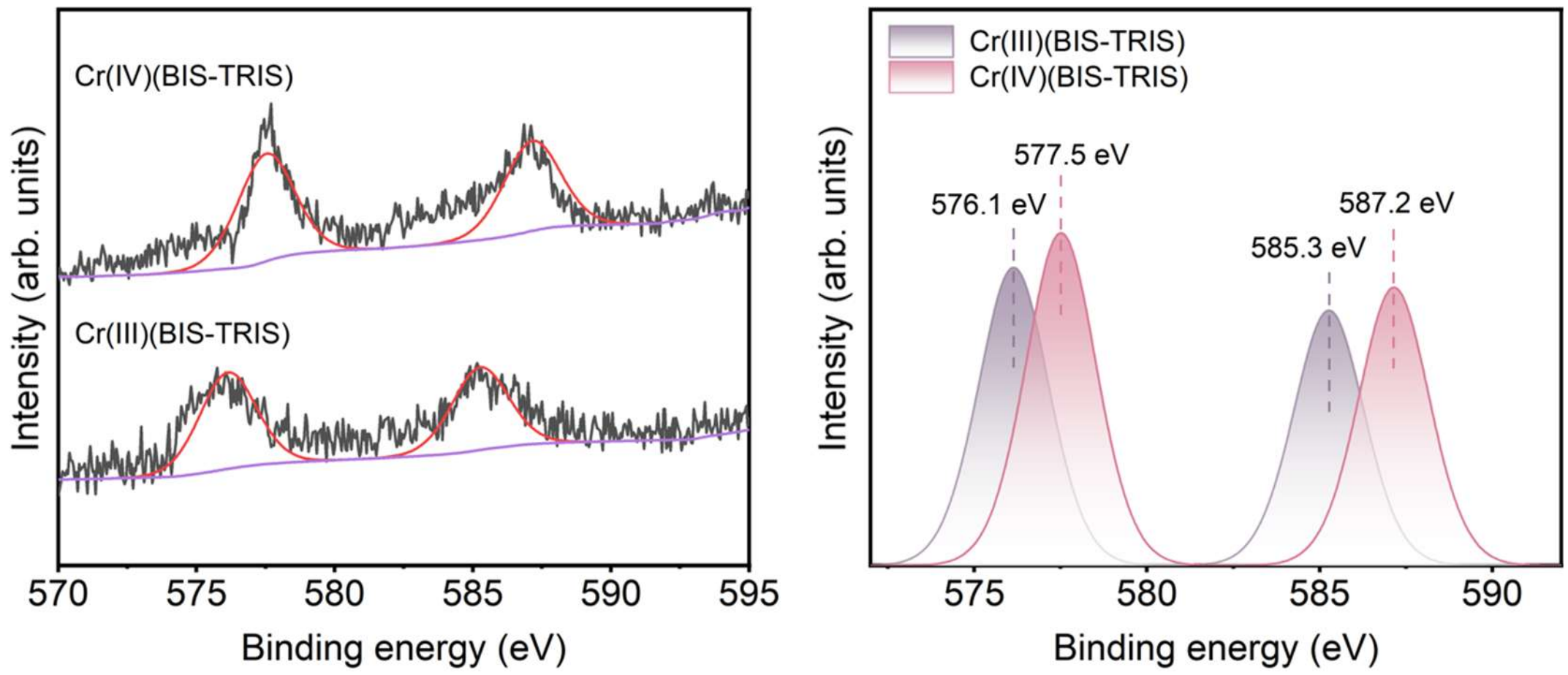

Supplementary Fig. 12 | Oxidation-state characterization of Cr(BIS-TRIS) by X-ray photoelectron spectroscopy (XPS). Samples were prepared from a 0.5 M Cr(BIS-TRIS) posolyte after the cell was discharged to 0% state of charge (SOC) or charged to 100% SOC at 80 mA cm^{-2} and 25 ± 1 °C. Ethanol at ten times the electrolyte volume was then added to precipitate the Cr(BIS-TRIS) complexes, and the resulting precipitates were collected for XPS measurements. Source data are provided as a Source Data file.

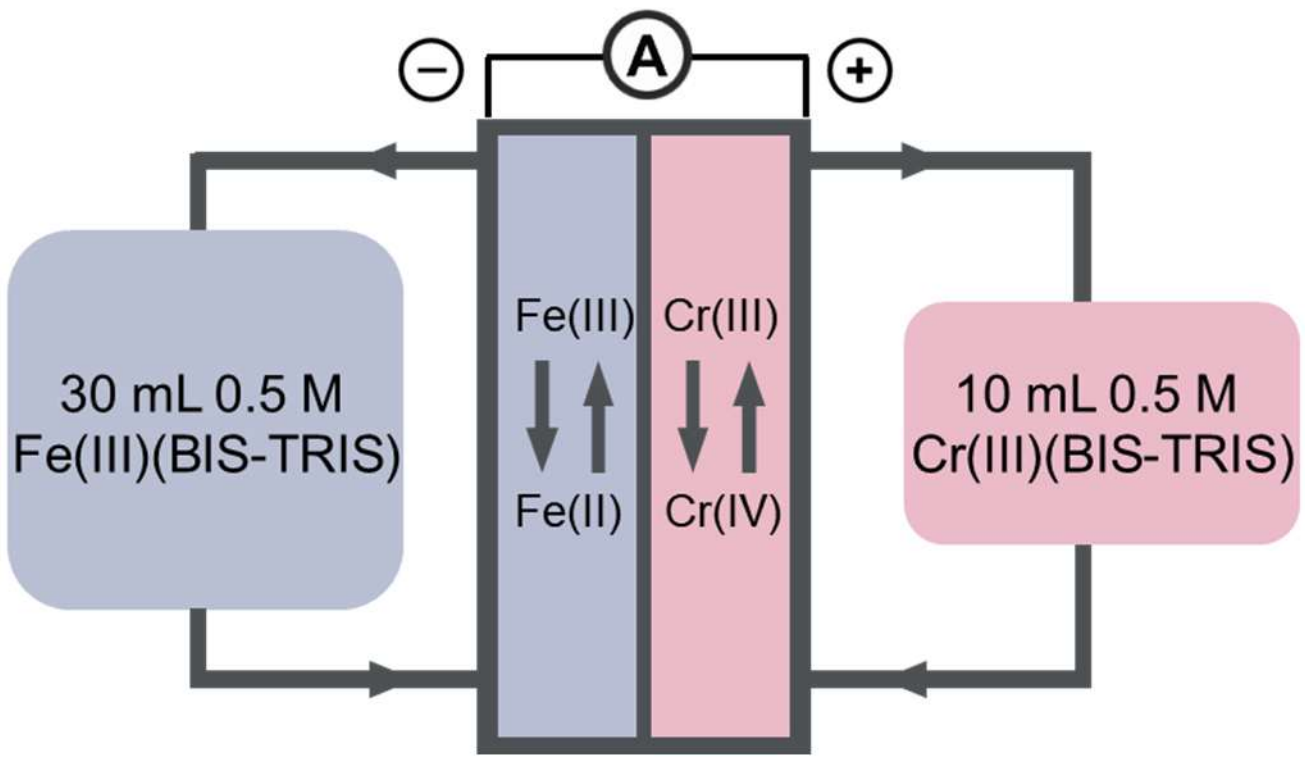

Supplementary Fig. 13 | Posolyte-capacity-limiting cell configuration used for Fig. 3b. A posolyte-capacity-limiting cell with 10 mL of 0.5 M Cr(III)(BIS-TRIS) posolyte and 30 mL of 0.5 M Fe(III)(BIS-TRIS) negolyte.

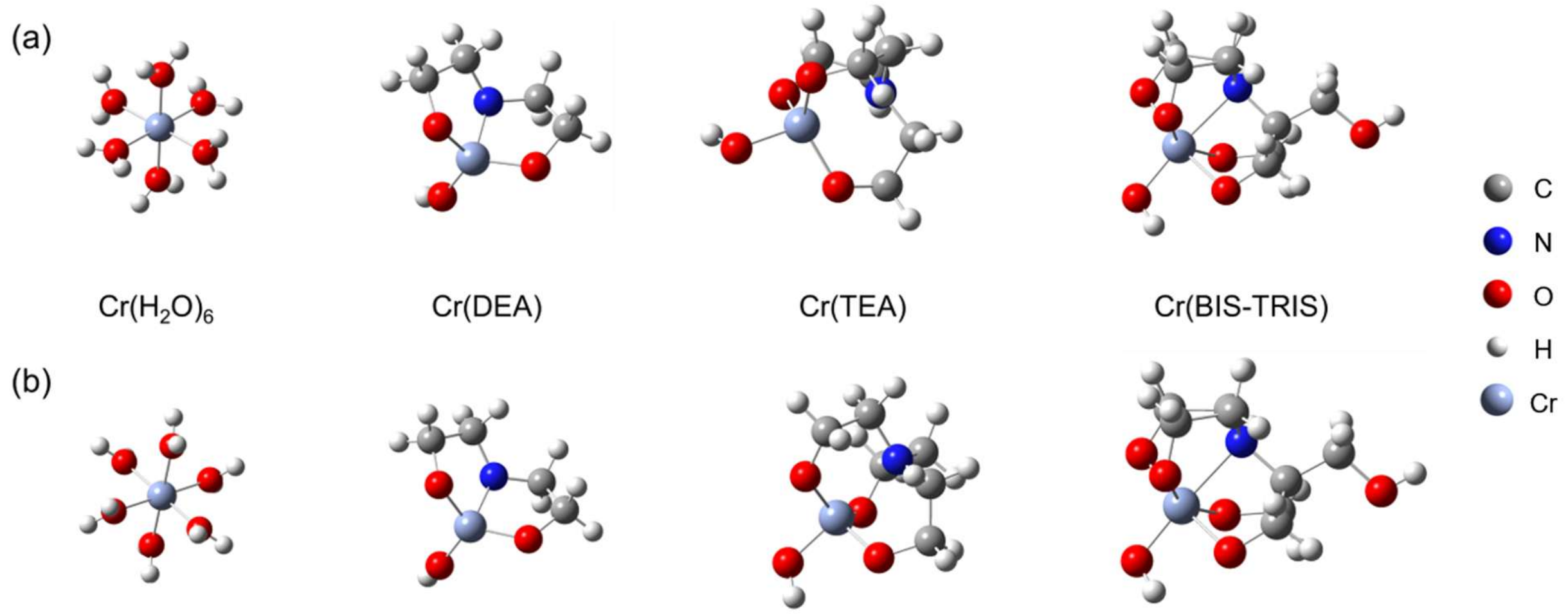

Supplementary Fig. 14 | Density functional theory (DFT) optimized structures of Cr(III) and Cr(IV) species. (a) Optimized structures of Cr(III) complexes with different ligands, including $Cr(H_2O)_6$, Cr(DEA), Cr(TEA), and Cr(BIS-TRIS). (b) Optimized structures of the corresponding Cr(IV) complexes.

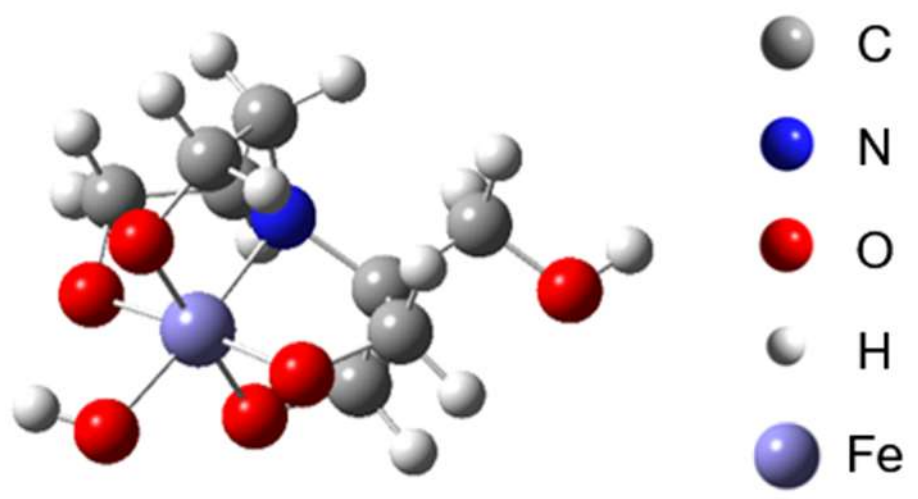

Supplementary Fig. 15 | Structure of Fe(BIS-TRIS) chelate[6].

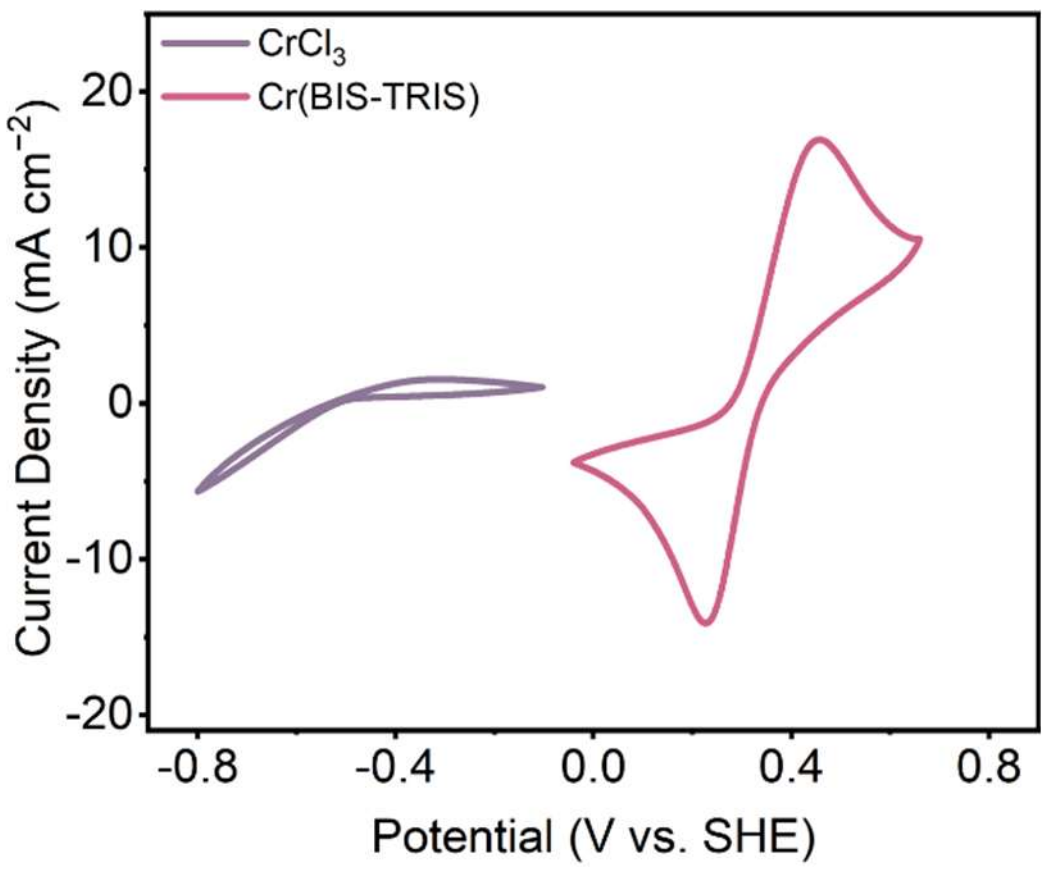

Supplementary Fig. 16 | Electrochemical comparison of $CrCl_3$ and Cr(BIS-TRIS). Cyclic voltammetry (CV) curves of 0.1 M $CrCl_3$ and 0.1 M Cr(BIS-TRIS) at a scan rate of 50 mV s^{-1}. All electrochemical tests were performed at 25 ± 1 °C. The potentials were not *iR* corrected. Source data are provided as a Source Data file.

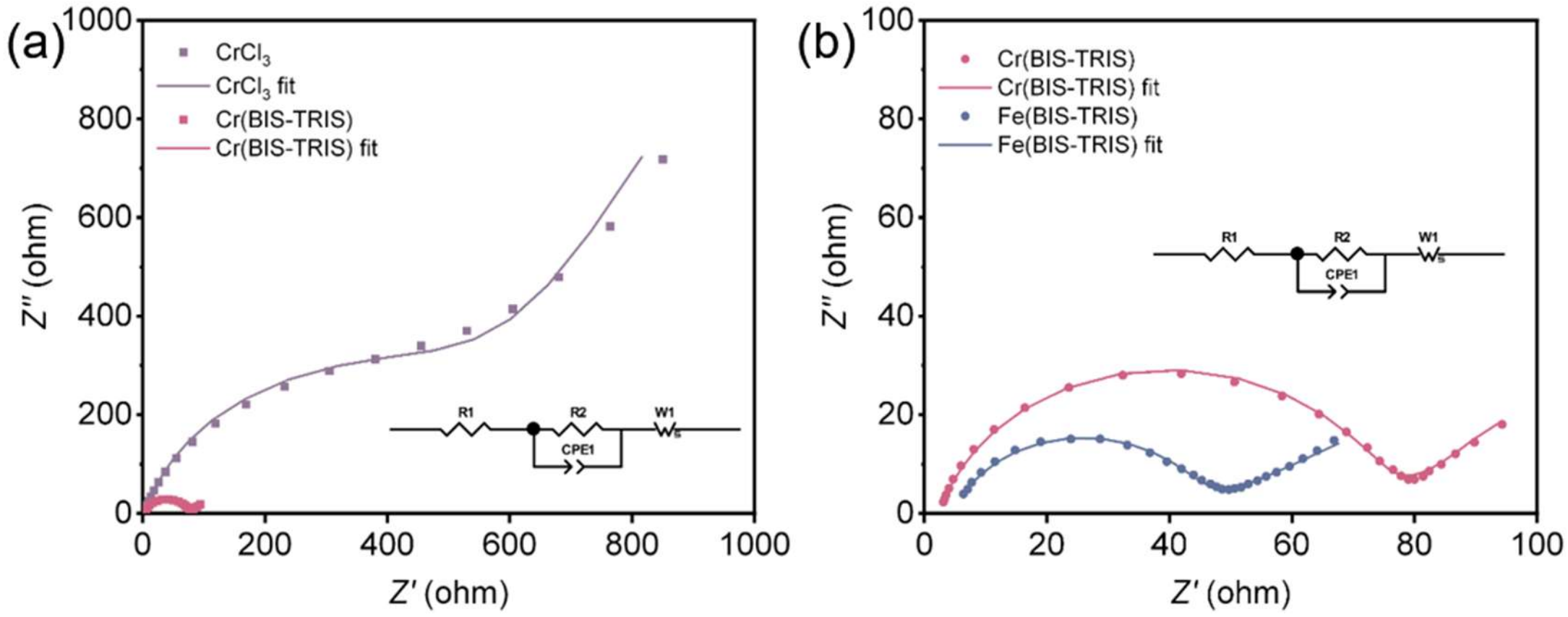

Supplementary Fig. 17 | Nyquist plots and equivalent-circuit fits of Cr(BIS-TRIS), $CrCl_3$, and Fe(BIS-TRIS). (a) A 0.1 M $CrCl_3$ solution containing 1 M HCl and a 0.1 M Cr(BIS-TRIS) solution containing 5 M KOH. (b) A 0.1 M Cr(BIS-TRIS) solution and a 0.1 M Fe(BIS-TRIS) solution, both containing 5 M KOH. Symbols represent the experimental data, and solid lines represent the equivalent-circuit fits. The insets show the equivalent circuit used for fitting. Measurements were performed at the open-circuit potential and 25 ± 1 °C. Source data are provided as a Source Data file.

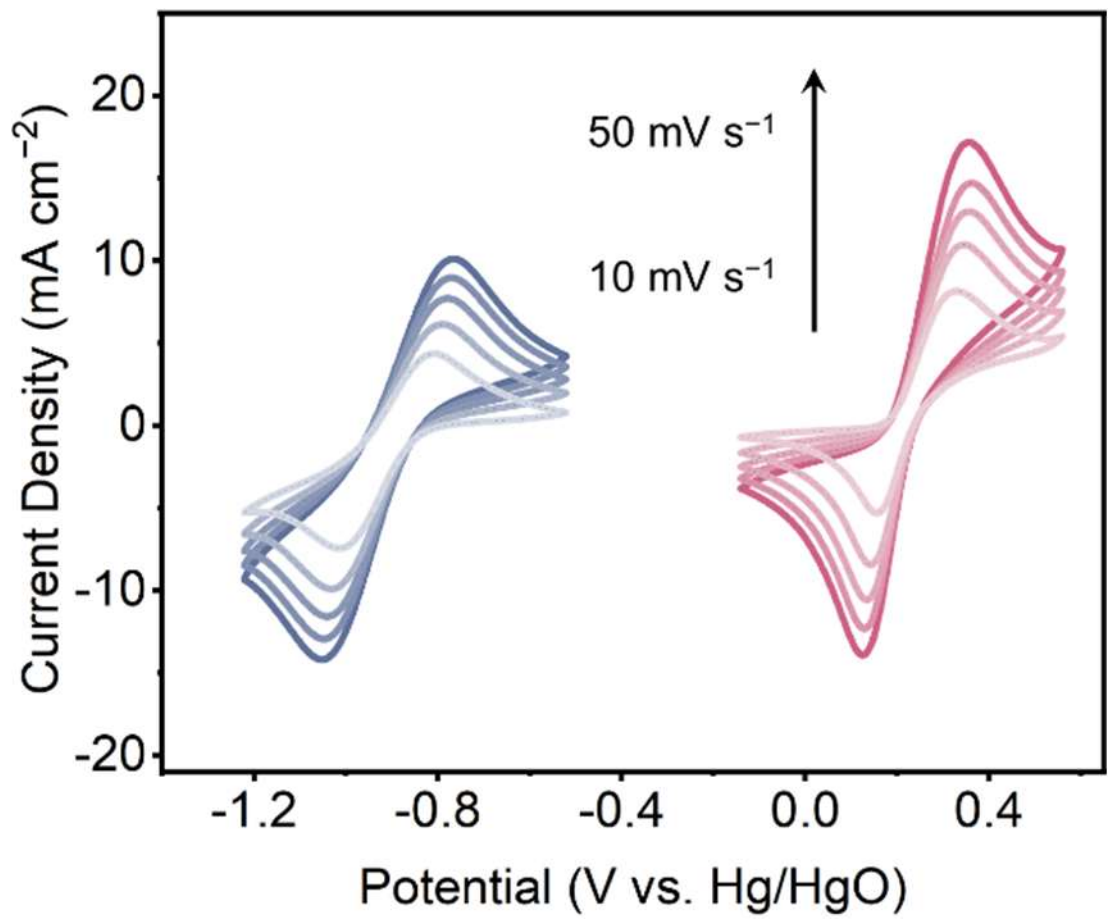

Supplementary Fig. 18 | Scan-rate dependence of Cr(BIS-TRIS) and Fe(BIS-TRIS) redox reactions. Cyclic voltammetry (CV) curves of 0.1 M Cr(BIS-TRIS) and 0.1 M Fe(BIS-TRIS) at different scan rates from 10 to 50 mV s^{-1}. All electrochemical tests were performed at 25 ± 1 °C. The potentials were not *iR* corrected. Source data are provided as a Source Data file.

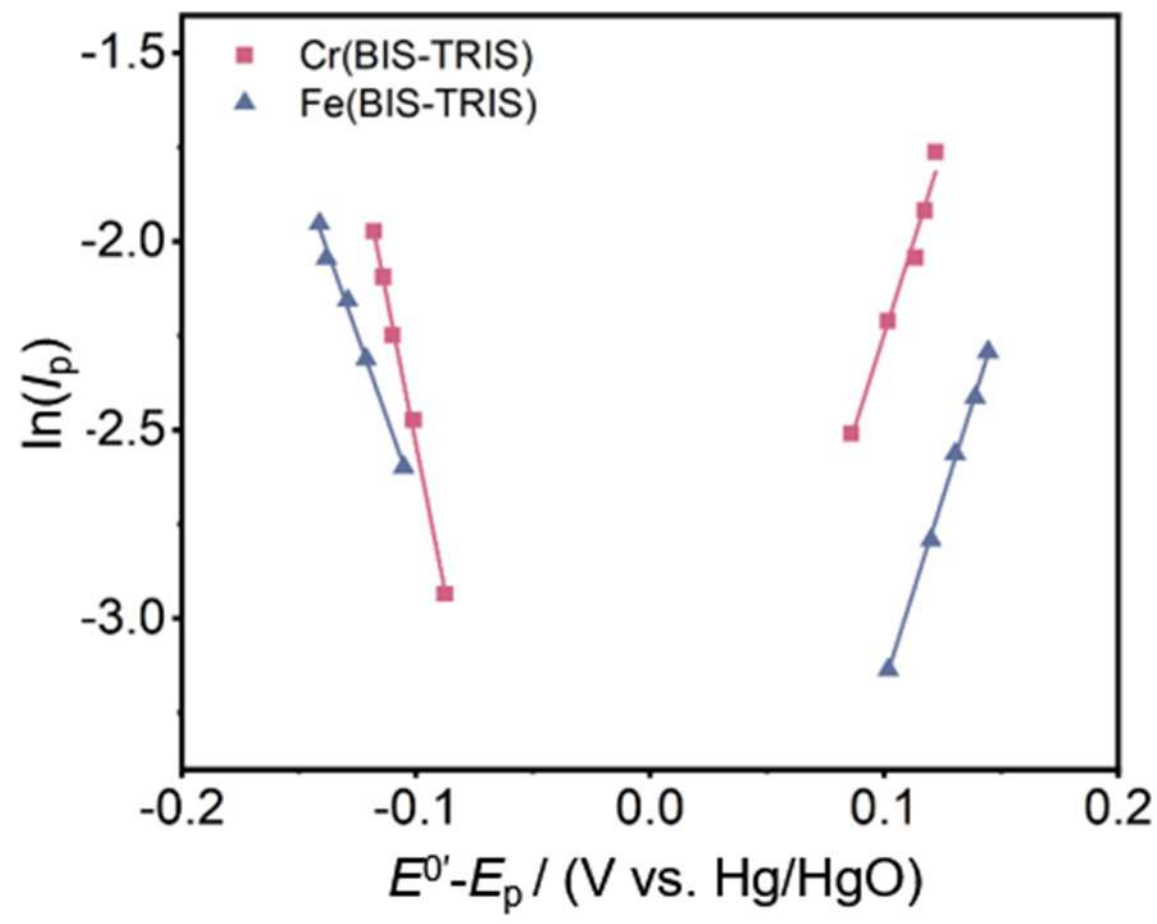

Supplementary Fig. 19 | Determination of standard electron-transfer rate constants of Cr(BIS-TRIS) and Fe(BIS-TRIS). Plots of ln(I_p) against $E^{0'}$-E_p for 0.1 M Cr(BIS-TRIS) and 0.1 M Fe(BIS-TRIS), used to determine the standard rate constants. All electrochemical tests were performed at 25 ± 1 °C. The potentials were not *iR* corrected. Source data are provided as a Source Data file.

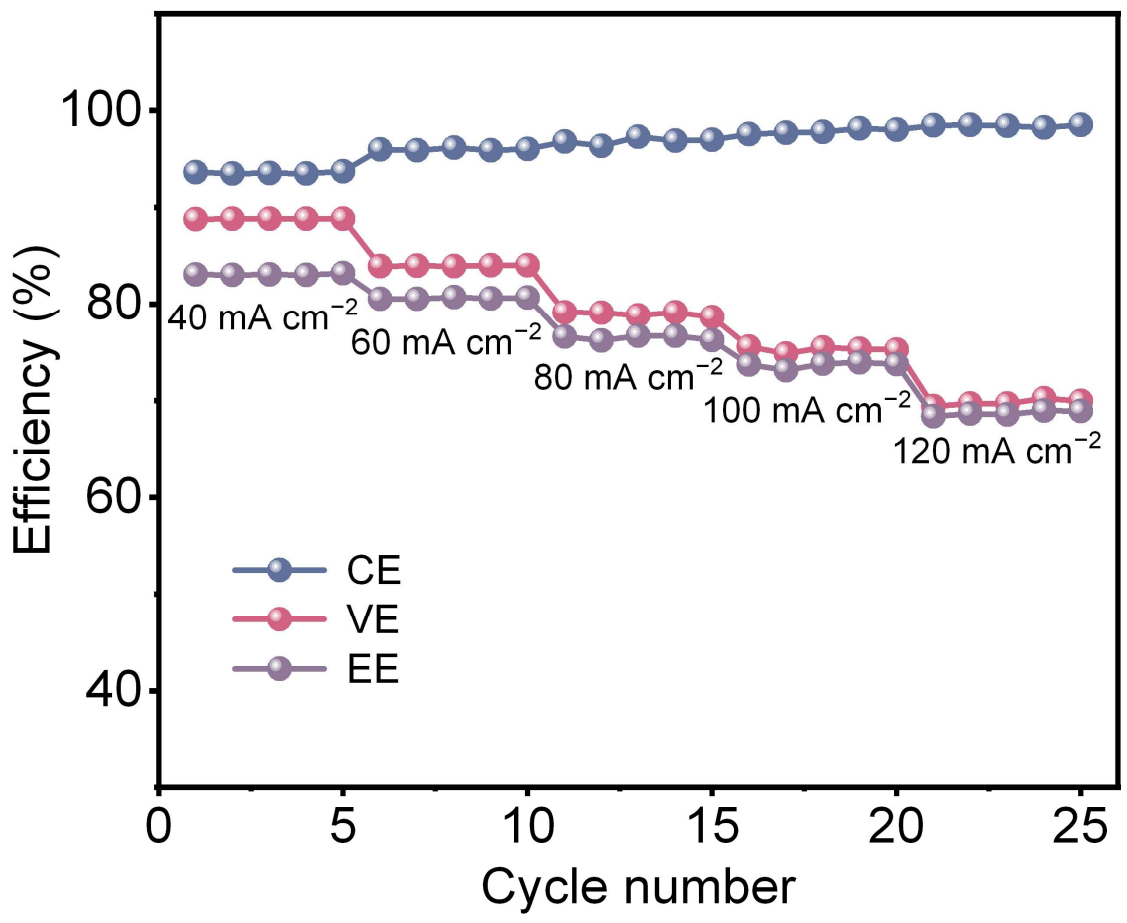

Supplementary Fig. 20 | Current-density-dependent efficiencies of the alkaline Fe-Cr redox flow battery (RFB). Coulombic efficiency (CE), voltage efficiency (VE), and energy efficiency (EE) values of the alkaline Fe-Cr RFB at 40, 60, 80, 100, and 120 mA cm^{-2}, with five consecutive charge-discharge cycles performed at each current density. All electrochemical tests were performed at 25 ± 1 °C. Source data are provided as a Source Data file.

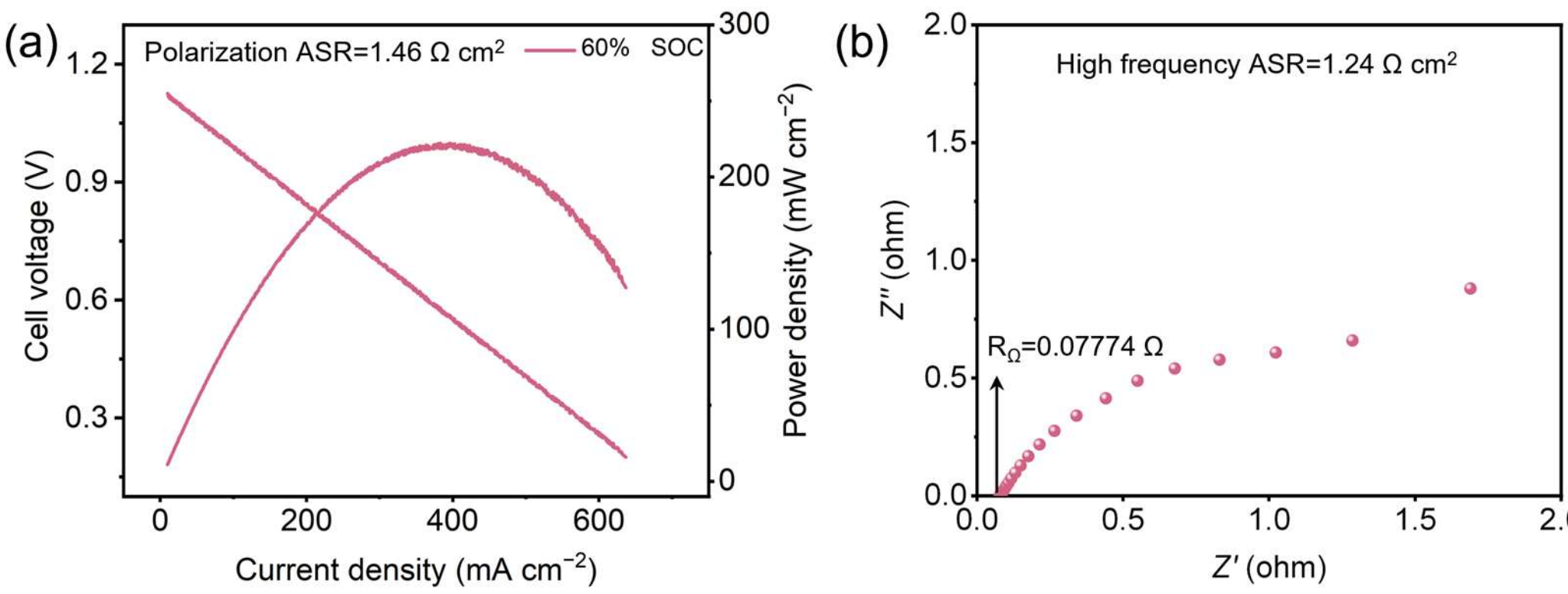

Supplementary Fig. 21 | Polarization and impedance of the alkaline Fe-Cr redox flow battery (RFB). (a) Polarization curve and (b) Nyquist plot of the 0.5 M Cr(BIS-TRIS)/Fe(BIS-TRIS) RFB. Before the measurements, the cell was charged to 60% state of charge (SOC) at 80 mA cm^{-2}. The high-frequency Ohmic resistance in b was determined by direct linear interpolation between the two adjacent high-frequency data points bracketing Z''=0. The points (Z', Z'')=(0.07703,-0.00194) and (0.07797,0.000629) yielded a real-axis intercept of 0.07774 Ω, corresponding to an area-specific resistance (ASR) of 1.24 Ω cm^2 based on an electrode area of 16 cm^2. The polarization curve in a yielded an ASR of 1.46 Ω cm^2. All electrochemical tests were performed at 25 ± 1 °C. Source data are provided as a Source Data file.

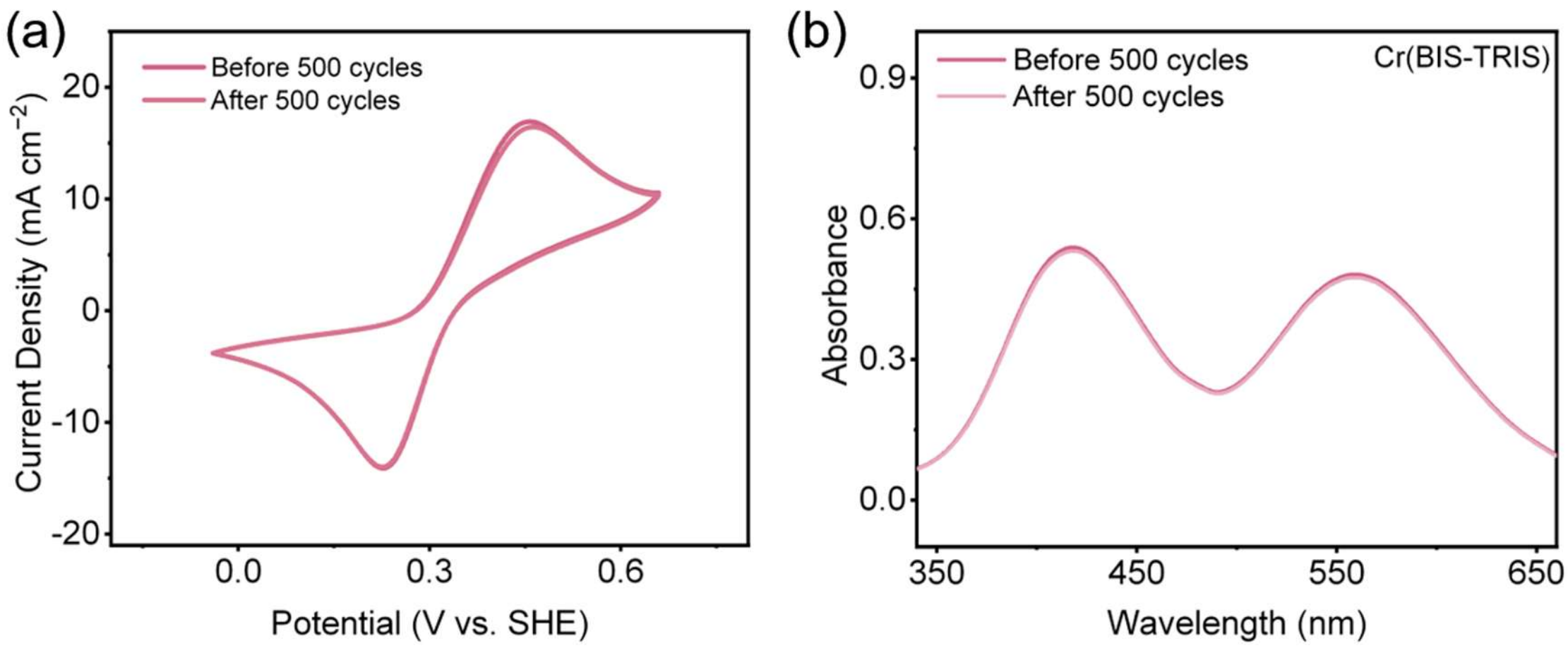

Supplementary Fig. 22 | Stability assessment of the Cr(BIS-TRIS) posolyte. (a) Cyclic voltammetry (CV) and (b) Ultraviolet-visible (UV-Vis) absorption spectra of the Cr(BIS-TRIS) posolyte with a ligand-to-metal ratio of 1.1:1 before battery cycling and after 500 charge-discharge cycles. The post-cycling sample was collected at the final discharge state (0% state of charge (SOC)) after 500 cycles at 100 mA cm^{-2} and 25 ± 1 °C. The potentials were not *iR* corrected. Source data are provided as a Source Data file.

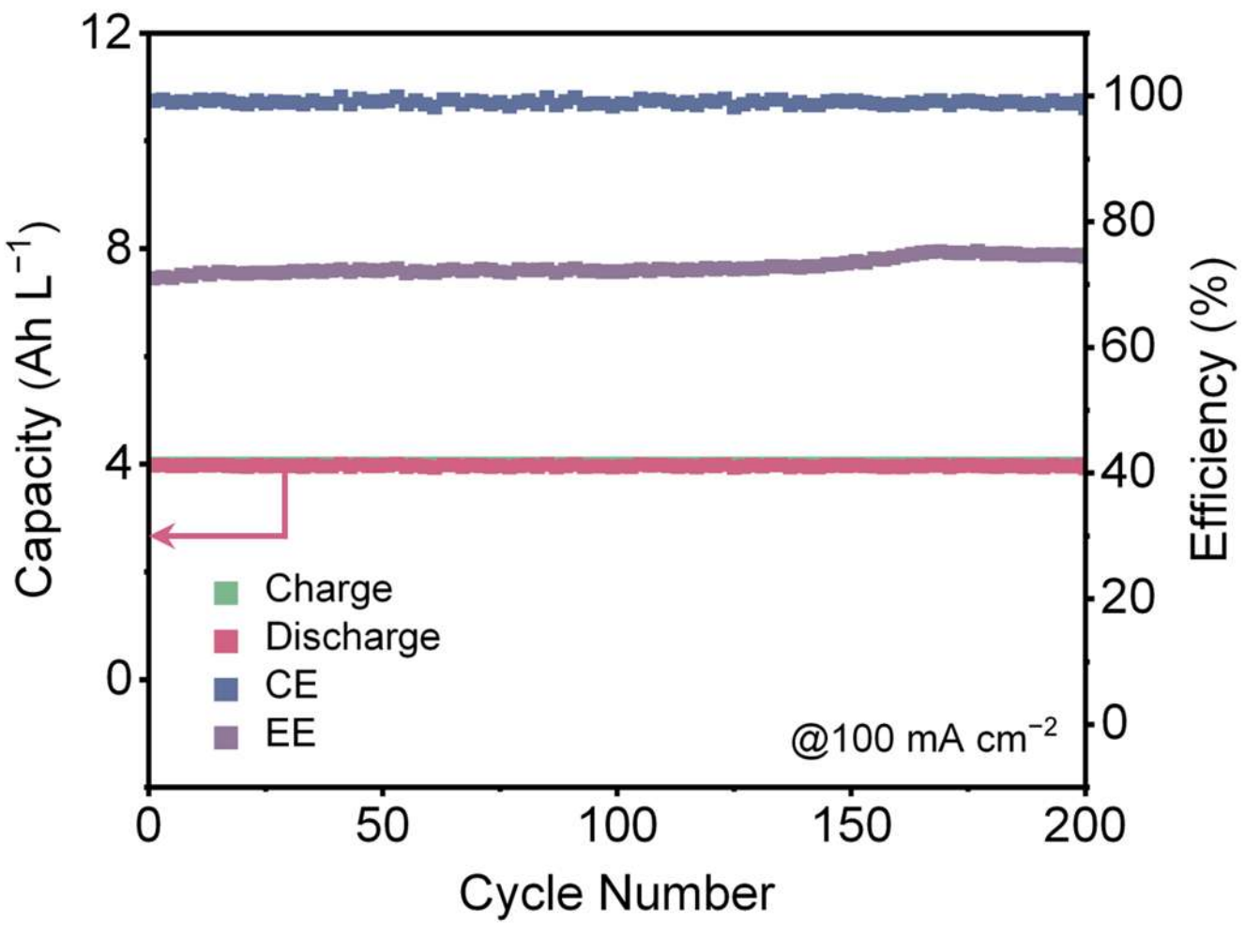

Supplementary Fig. 23 | Cycling performance of the alkaline Fe-Cr redox flow battery (RFB) with a mixed electrolyte at 80% state of charge (SOC). All electrochemical tests were performed at 25 ± 1 °C. Source data are provided as a Source Data file. The posolyte and negolyte were prepared using the same mixed electrolyte, with 10 mL of electrolyte used for each side.

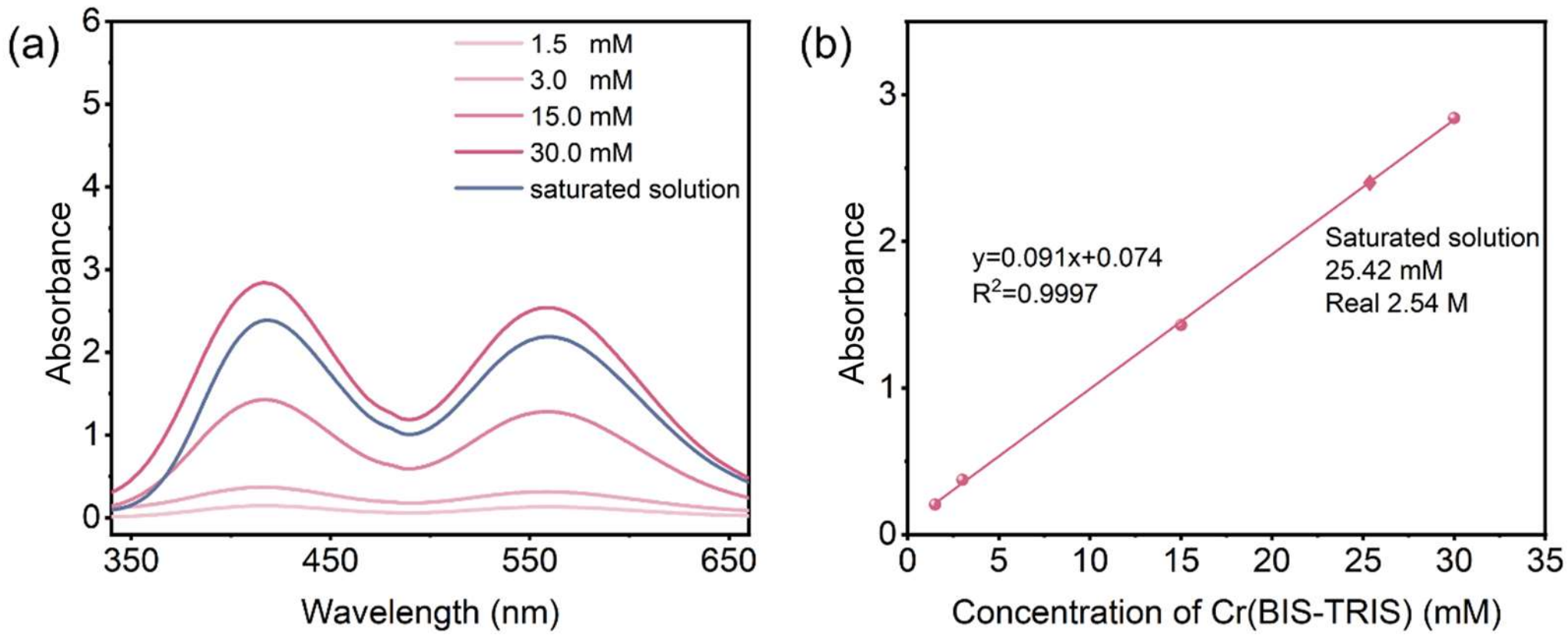

Concentration (mM)	Peak intensity
1.5	0.204
3.0	0.372
15	1.428
30	2.841
saturated solution	2.387

Supplementary Fig. 24 | Ultraviolet-visible (UV-Vis) determination of Cr(BIS-TRIS) solubility. (a) The UV-Vis spectra of Cr(III)(BIS-TRIS) solutions with various concentrations (1.5 mM, 3.0 mM, 15 mM, 30 mM, and the 100-fold diluted saturated solution). (b) The linear relationship between concentration and peak intensity at 437 nm. The inset table indicates the peak intensity of each solution at 437 nm. Source data are provided as a Source Data file.

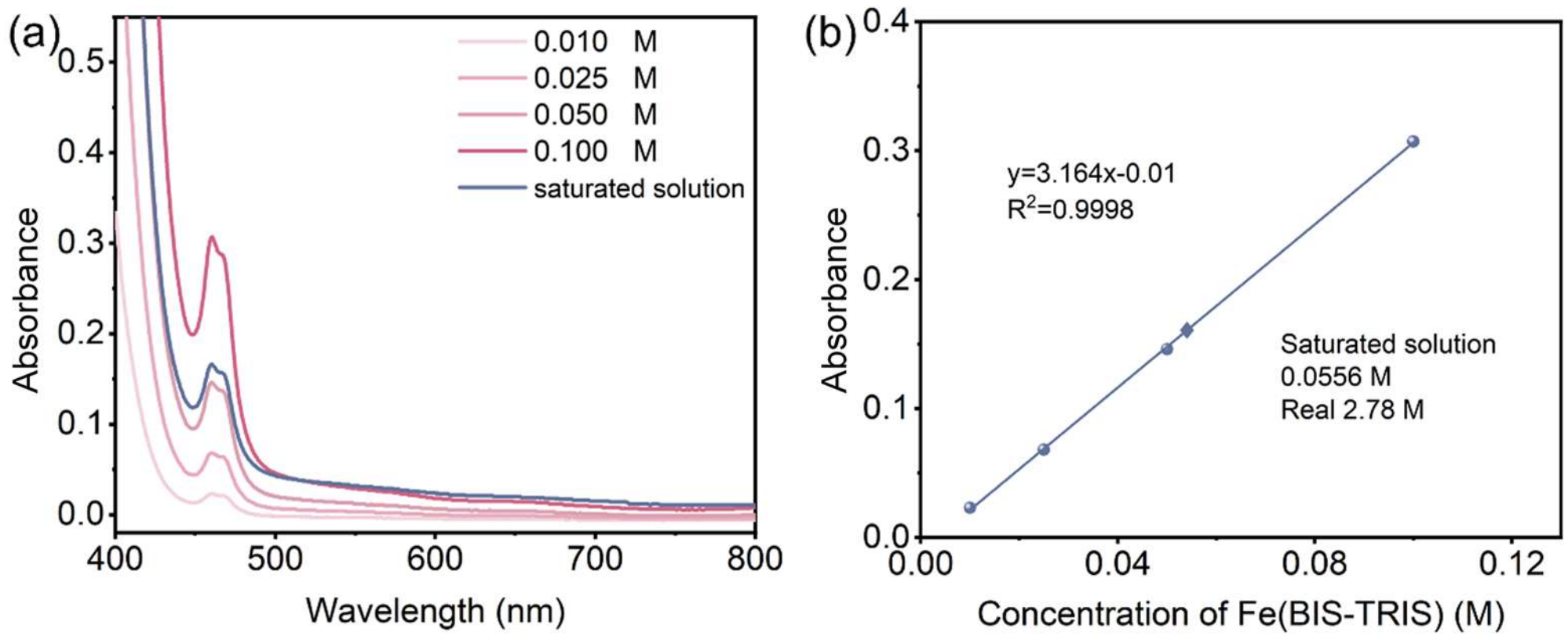

Concentration (M)	Peak intensity
0.01	0.023
0.025	0.068
0.05	0.146
0.1	0.307
saturated solution	0.166

Supplementary Fig. 25 | Ultraviolet-visible (UV-Vis) determination of Fe(BIS-TRIS) solubility. (a) The UV-Vis spectra of Fe(III)(BIS-TRIS) solutions with various concentrations (0.01 M, 0.025 M, 0.05 M, 0.1 M, and 50-fold diluted saturated solution). (b) The linear relationship between concentration and peak intensity at 460 nm. The inset table indicates the peak intensity of each solution at 460 nm. Source data are provided as a Source Data file.

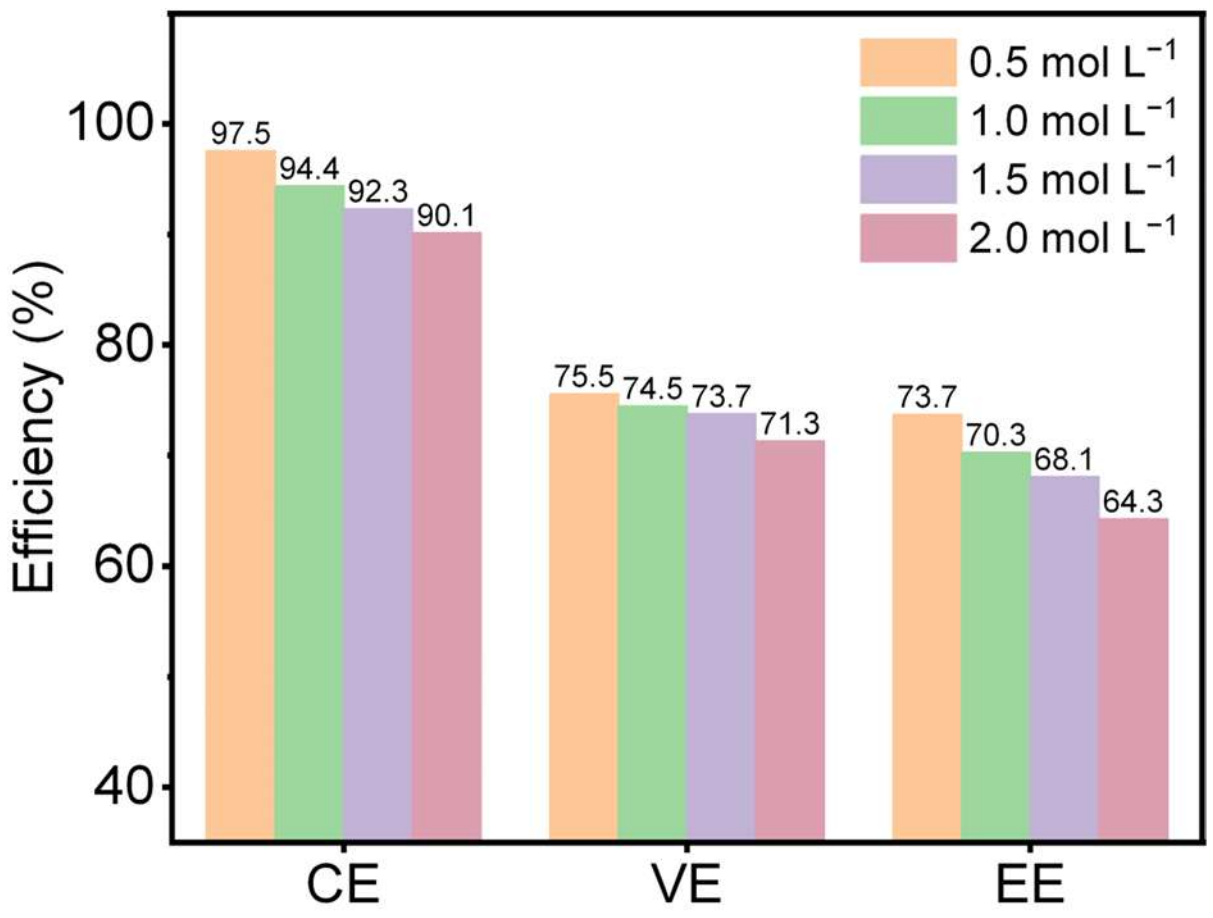

Supplementary Fig. 26 | Concentration-dependent efficiencies of alkaline Fe-Cr redox flow battery. Coulombic efficiency (CE), voltage efficiency (VE), and energy efficiency (EE) of alkaline Fe-Cr RFBs using different concentrations of electrolytes at 100% state of charge (SOC) and 100 mA cm^{-2}. All electrochemical tests were performed at 25 ± 1 °C. Source data are provided as a Source Data file.

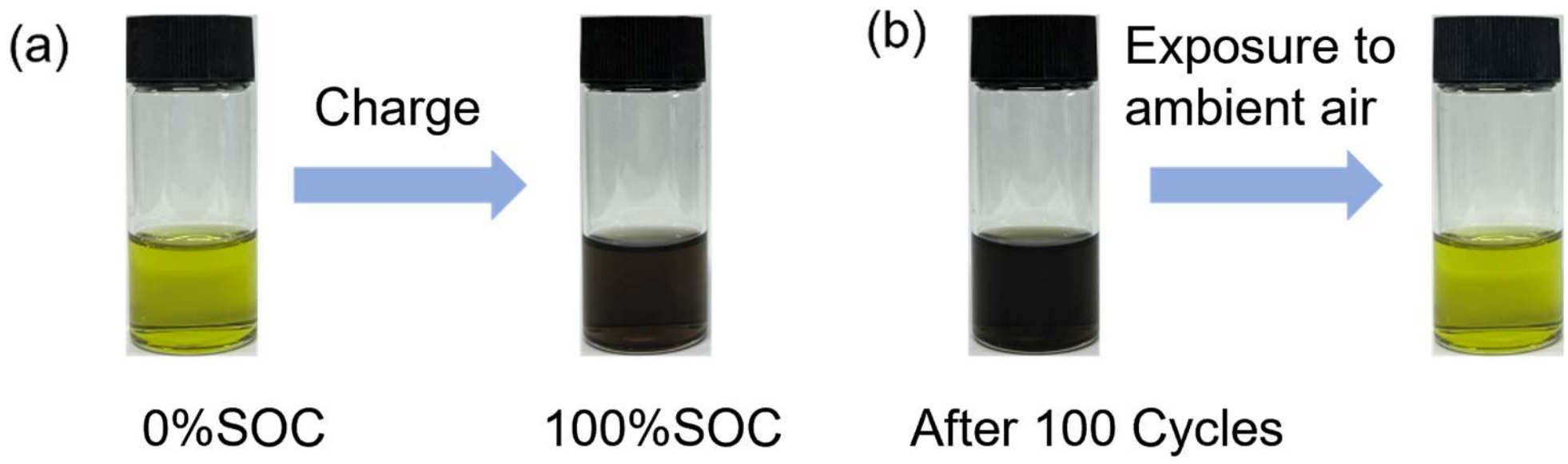

Supplementary Fig. 27 | Cycling-induced colour changes and air recovery of the Fe(BIS-TRIS) negolyte. (a) Optical images of the Fe(BIS-TRIS) negolyte collected at 0% and 100% state of charge (SOC) during the first charge-discharge cycle at 100 mA cm^{-2} and 25 ± 1 °C. (b) Optical images of the Fe(BIS-TRIS) negolyte after 100 cycles at 100 mA cm^{-2} and 25 ± 1 °C. The electrolyte samples after cycling were collected at the final discharge state (0% SOC). After exposure to air for 6 h at 25 ± 1 °C, the negolyte returned to its pre-cycling colour. Source data are provided as a Source Data file.

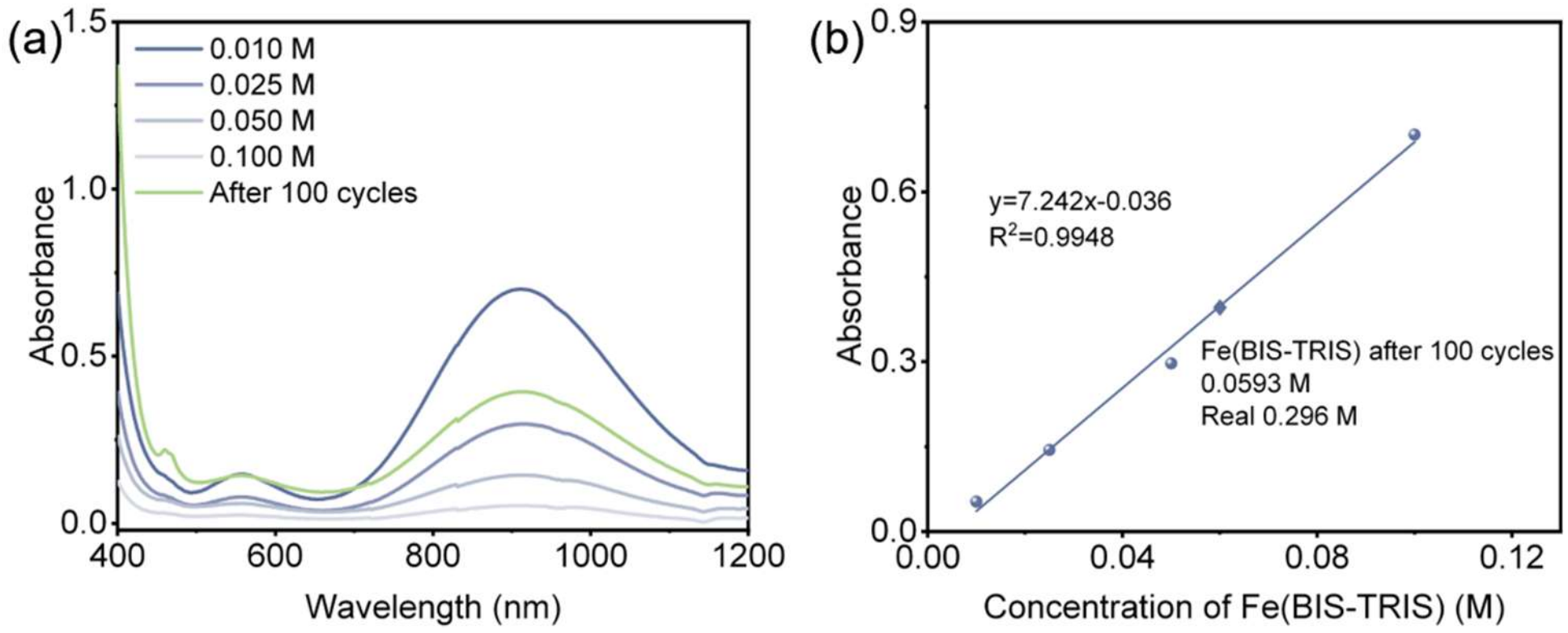

Concentration (M)	Peak intensity
0.01	0.053
0.025	0.144
0.05	0.297
0.1	0.701
post-cycling Fe(BIS-TRIS) negolyte	0.393

Supplementary Fig. 28 | Ultraviolet-visible (UV-Vis) quantification of Fe(BIS-TRIS) after cycling. (a) UV-Vis spectra of Fe(II)(BIS-TRIS) solutions at different concentrations (0.01, 0.025, 0.05 and 0.1 M) and a fivefold-diluted post-cycling Fe(BIS-TRIS) negolyte. The post-cycling sample was collected from a capacity-balanced cell at the end of the 100th discharge (0% state of charge (SOC)) after cycling at 100 mA cm^{-2} and 25 ± 1 °C. (b) Linear relationship between Fe(II)(BIS-TRIS) concentration and peak intensity at 911 nm. The inset table lists the peak intensity of each solution at 911 nm. Source data are provided as a Source Data file.

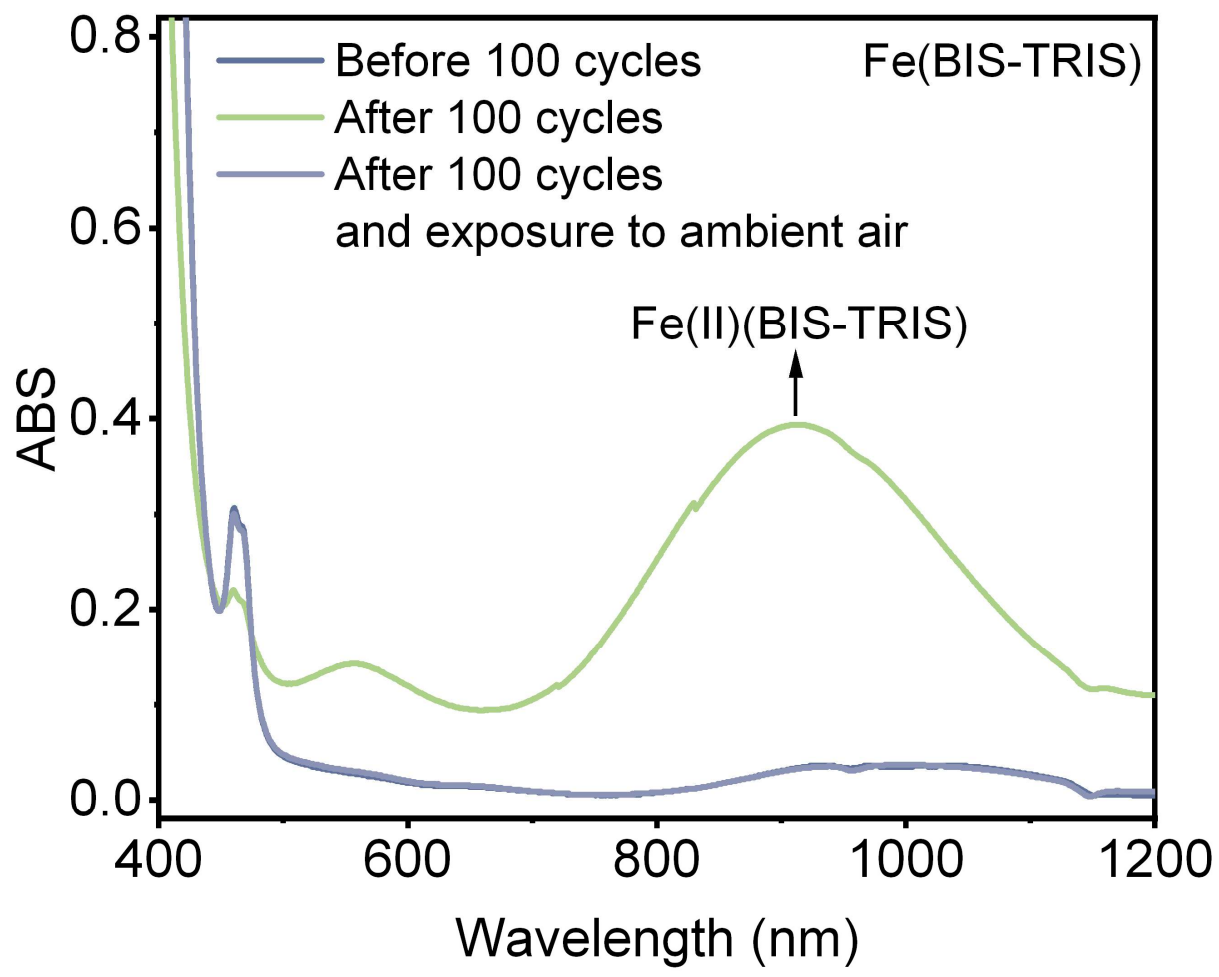

Supplementary Fig. 29 | Spectroscopic recovery of the cycled Fe(BIS-TRIS) negolyte after air exposure. Ultraviolet-visible (UV-Vis) spectra of the 0.5 M Fe(BIS-TRIS) negolyte before cycling, after 100 charge-discharge cycles at 100 mA cm^{-2} and 25 ± 1 °C, with the final discharge state corresponding to 0% state of charge (SOC), and after subsequent exposure to air for 6 h at 25 ± 1 °C. Before measurement, the samples were diluted fivefold with 1 M KOH. Source data are provided as a Source Data file.

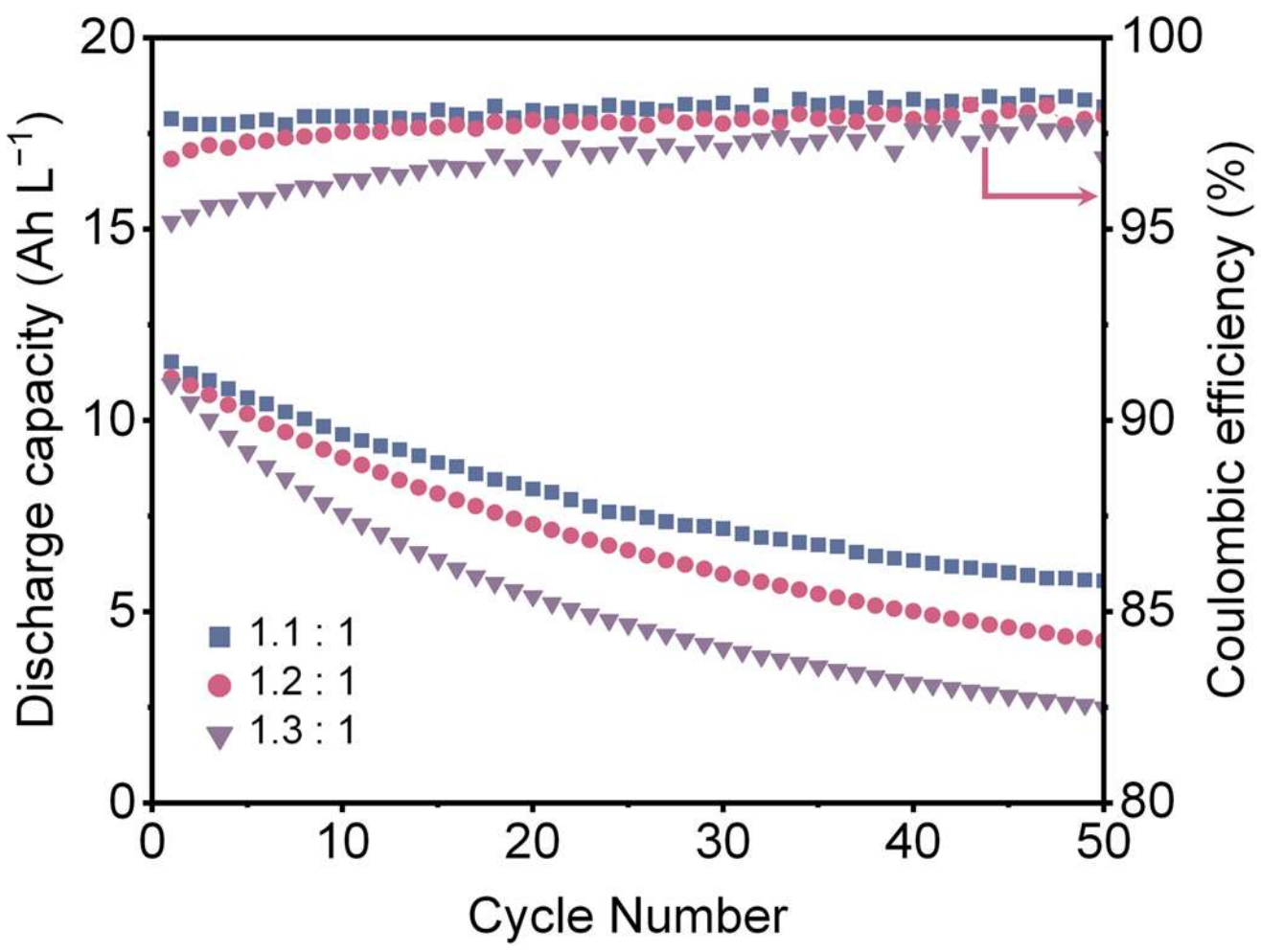

Supplementary Fig. 30 | Effect of the posolyte ligand-to-metal ratio on cycling performance. Cycling performance of a 0.5 M alkaline Fe-Cr redox flow battery (RFB) with varying ligand-to-metal ratios in the Cr(BIS-TRIS) posolyte at 100% state of charge (SOC) and 100 mA cm^{-2}. All electrochemical tests were performed at 25 ± 1 °C. Source data are provided as a Source Data file.

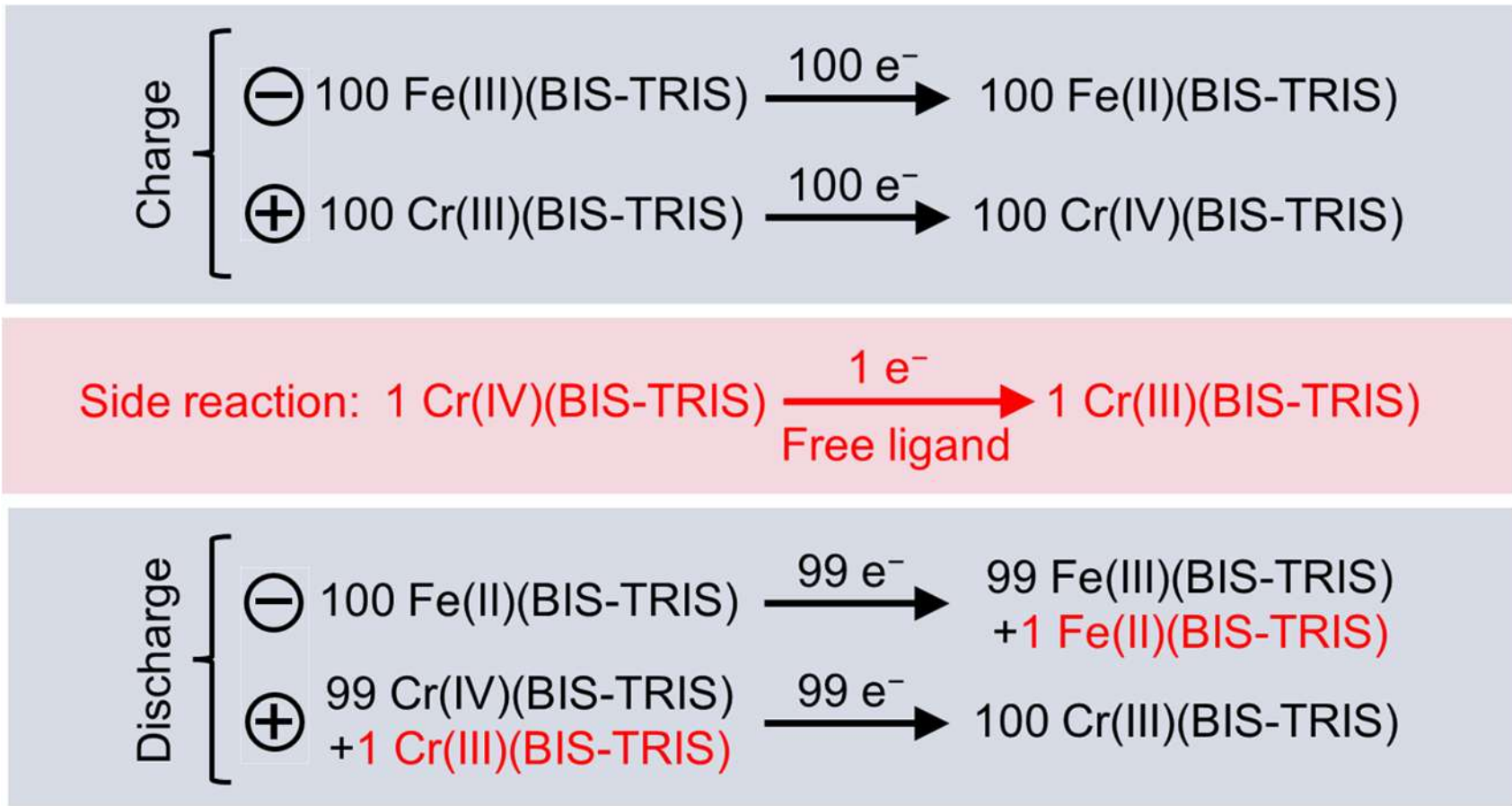

Supplementary Fig. 31 | Proposed capacity-decay mechanism involving Cr(IV)(BIS-TRIS) and free BIS-TRIS.

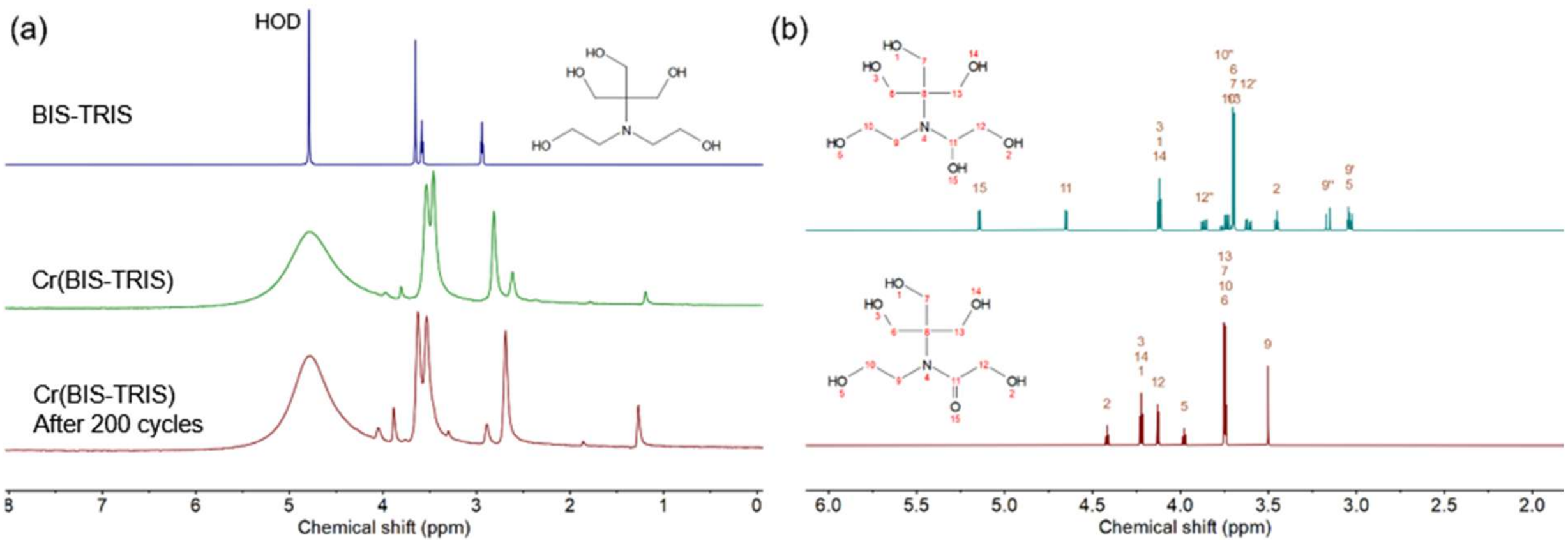

Supplementary Fig. 32 | ^{1}H nuclear magnetic resonance spectroscopy (NMR) analysis of the Cr(BIS-TRIS) posolyte and proposed ligand-oxidation products. (a) ^{1}H NMR spectra of BIS-TRIS and Cr(BIS-TRIS) before cycling and of the Cr(BIS-TRIS) posolyte collected at 100% state of charge (SOC) after 200 charge-discharge cycles at 100 mA cm^{-2} and 25 ± 1 °C. (b) Simulated ^{1}H NMR spectra of the proposed ligand-oxidation products. Source data are provided as a Source Data file.

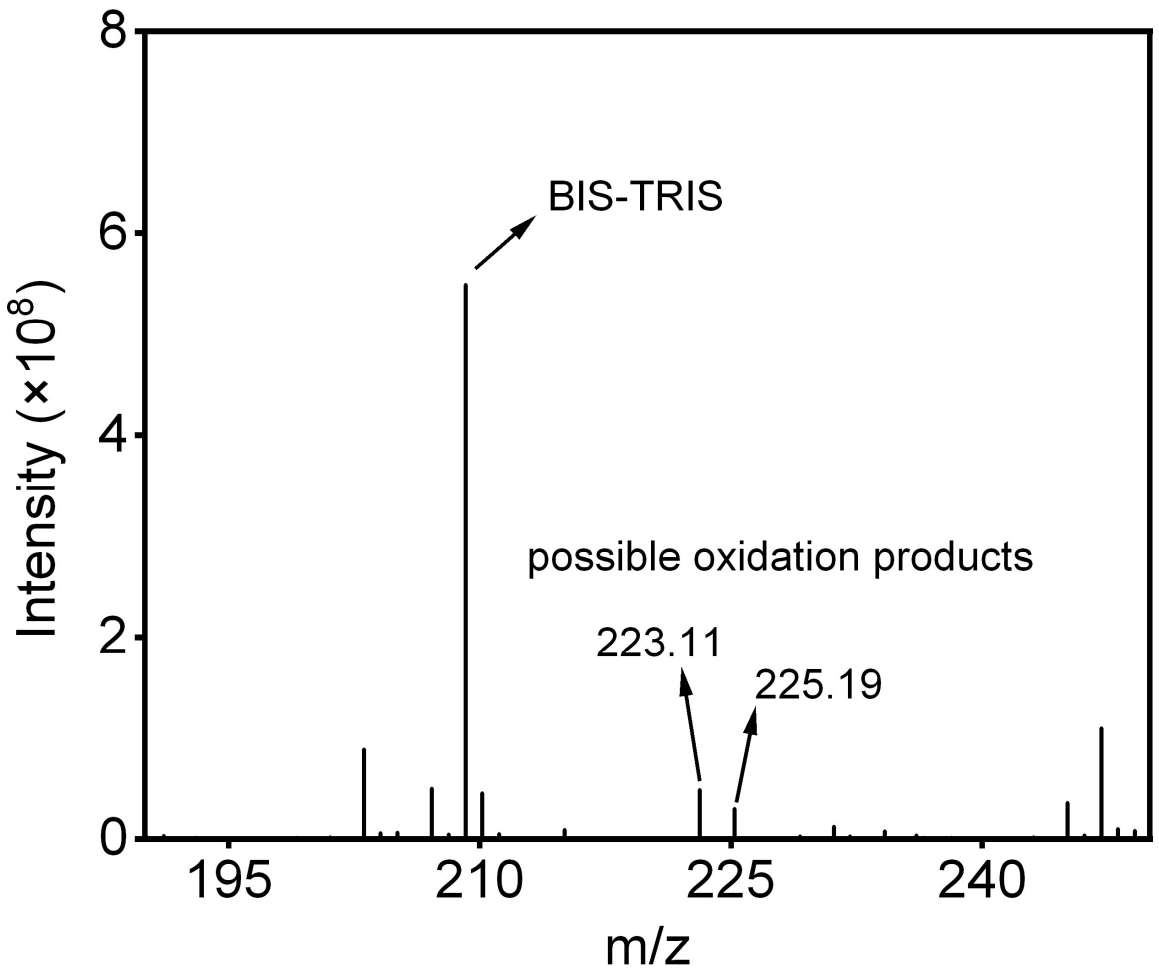

Supplementary Fig. 33 | Electrospray ionization high-resolution mass spectrometry (ESI-HRMS) characterization of the Cr(BIS-TRIS) posolyte after cycling. The sample was collected at 100% state of charge (SOC) after 200 charge-discharge cycles at 100 mA cm^{-2} and 25 ± 1 °C. Source data are provided as a Source Data file.

Supplementary Table 1. EXAFS fitting results for the samples.

Sample	Shell	CN^a	R(Å)^b	σ^2 (Å^2)^c	ΔE_0(eV)^d	R factor
Cr-foil	Cr-Cr	12*	2.49±0.05	0.0069	5.5±0.2	0.013
Cr_2O_3	Cr-O	6*	1.98±0.06	0.0012	3.5±0.3	0.007
	Cr-O-Cr	6*	2.94±0.01	0.0082		
Cr(BIS-TRIS)	Cr-O	4.98±0.5	1.95±0.09	0.0023	2.0±0.6	0.011
	Cr-N	1.11±0.1	1.99±0.09	0.0087		

$^a CN$: coordination numbers; $^b R$: bond distance; $^c \sigma^2$: Debye-Waller factors; ^{d} ΔE_0: the inner potential correction. The R factor indicates the goodness of fit.

Supplementary Table 2. Calculated energy changes during Cr(III/IV) redox reactions of different Cr species.

Species	Cr(III)	Cr(IV)	ΔE (Hartree)	ΔE (eV)
$Cr(H_2O)_6$	-1502.06985	-1501.15912	0.910731	24.78099051
Cr(DEA)	-1482.7596	-1482.5250	0.2346	6.383466
Cr(TEA)	-1636.66896	-1636.618541	0.050422	1.37198262
Cr(BIS-TRIS)	-1864.98162	-1865.017974	-0.036354	-0.98919234

Supplementary Table 3. Impedance parameters and fitting errors obtained using the equivalent circuit R_1-(R_2||CPE_1)-W_1 for Fe(BIS-TRIS), Cr(BIS-TRIS) and $CrCl_3$ at the glassy carbon electrode. R_1 represents the solution resistance (R_0), R_2 the charge-transfer resistance (R_{ct}), CPE_1 the constant-phase element associated with non-ideal double-layer capacitance, and W_1 the Warburg diffusion element. χ^2 is the chi-square goodness-of-fit statistic reported by ZView.

Species	R_0 (Ω)	R_{ct} (Ω)	χ^2
Cr(BIS-TRIS)	2.342	71.76	0.005237

Fe(BIS-TRIS)	4.236	39.08	0.008374
$CrCl_3$	2.704	517.5	0.024895

Supplementary Table 4. Diffusion Coefficients and standard rate constants of Fe(BIS-TRIS) and Cr(BIS-TRIS) chelates.

Species	D_O (cm^2 s^{-1})	D_R (cm^2 s^{-1})	K_O (cm s^{-1})	K_R (cm s^{-1})
Cr(BIS-TRIS)	6.93×10^{-6}	6.62×10^{-6}	6.85×10^{-3}	1.87×10^{-3}
Fe(BIS-TRIS)	3.03×10^{-6}	4.07×10^{-6}	2.79×10^{-3}	4.92×10^{-3}

Supplementary Table 5. The electrochemical properties of Cr-based active materials.

Species	Active-species concentration	Supporting electrolyte	Working electrode	Temperature	Analysis method	D_O (cm^2 s^{-1})	K_O (cm s^{-1})	Ref.
Cr(BIS-TRIS)	0.10 M	KOH	Glassy carbon	25 ± 1 °C	Randles-Ševčík (*D*); Nicholson-Shain peak-current analysis (*K*)	6.93×10^{-6}	6.85×10^{-3}	This work
$K_3[Cr(CN)_6]$	5 mM	NaCN	Glassy carbon	NR	Randles-Ševčík (*D*); Nicholson method (*K*)	7.16×10^{-6}	6.03×10^{-3}	7
Cr(EDTA)	5 mM	Acetate buffer	Graphite-disc	NR	Randles-Ševčík (*D*)	8.1×10^{-6}	NR	8
Cr(PDTA)	5 mM	Borate buffer	Glassy carbon	25 °C	Randles-Ševčík (*D*); Nicholson-Shain peak-current analysis (*K*)	6.2×10^{-6}	1.7×10^{-4}	9
$Cr(acac)_3$	0.05 M	$TEABF_4$/MeCN	Pt wire	Room temperature	Randles-Ševčík (*D*); exponential peak-current analysis (*K*)	4.91×10^{-6}	1.52×10^{-3}	10
$CrCl_3$	NR	KCl	Glassy carbon	NR	NR	NR	$< 10^{-6}$	11
$Cr(f\text{-}DPA)_2$	NR	KCl	Glassy carbon	NR	Nicholson method (*K*)	NR	10^{-3}	11

$CrCl_3$ (with Bi catalyst)	NR	Acidic electrolyte; composition NR	Bi-based electrode	NR	NR	6.2 $\times 10^{-6}$	1.35 $\times 10^{-3}$	[12]

NR, not reported.

Supplementary Table 6. Comparison of the concentrations and theoretical capacities of the redox-active species shown in **Fig. 4g**. All capacities were calculated assuming a one-electron reaction and are expressed per litre of the electrolyte containing the listed redox-active species, rather than per total electrolyte volume.

Species	Concentration (M)	Supporting electrolyte	pH	Counterion	Temperature	Theoretical Capacity (Ah L^{-1})	Ref.
$Fe(acac)_3$	0.37	TEATFSI/MeCN	NR	NR	NR	9.916	[13]
Fe(DIPSO)	0.6	KOH/NaOH	≥14	K^+, Na^+	NR	16.08	[14]
$Fe(CN)_6$	0.7	Water	Near-neutral	Na^+	Room temperature	18.76	[15]
$Cr(f\text{-}DPA)_2$	0.71	Water	NR	Br^-	NR	19.028	[11]
Fe(TIPA)	0.81	KOH	≥14	K^+	NR	21.708	[16]
Cr(PDTA)	1	NR	9–10	K^+	Room temperature	26.8	[9]
Fe(TEA)	1.1	KOH	≈14	K^+	NR	29.48	[17]
$Cr(CN)_6$	1.1	NaCN	NR	K^+, Na^+	NR	29.48	[7]
Fe(DTPA)	1.35	NR	9	K^+	NR	36.18	[18]
Cr(BIS-TRIS)	2.54	KOH/NaOH	≥14	K^+, Na^+	25 ± 1 °C	68.07	This work
Fe(BIS-TRIS)	2.78	KOH/NaOH	≥14	K^+, Na^+	25 ± 1 °C	74.5	This work

Supplementary Table 7. The raw material prices were collected from publicly available market quotations on Guidechem (https://china.guidechem.com/).

Material	Molecular weight (g/mol)	Price ($/kg)
$CrCl_3 \cdot 6H_2O$	266.48	0.41
$FeCl_3 \cdot 6H_2O$	270.3	0.35

BIS-TRIS	209.24	0.10
KOH	56.11	0.28

References

1. Ayari, F. et al. Ammoxidation of ethylene over low and over-exchanged Cr-ZSM-5 catalysts. *Appl. Catal. Gen.* **415-416**, 132-140 (2012).
2. Pantazopoulou, E. et al. Stabilization of tannery sludge by co-treatment with aluminum anodizing sludge and phytotoxicity of end-products. *Waste Manage.* **61**, 327-336 (2017).
3. E, T. et al. Recovering Cr(III) from chromium-containing waste: An in-depth study on mechanism via retaining organic matters. *Environ. Chem. Eng.* **9**, 105598 (2021).
4. Buslaev, GS. et al. Synthesis, molecular structure, and vibrational spectra of tetrakis(2-hydroxyethylammonium) chloride and its triethanolamine precursor and metabolite. *Russ. J. Gen. Chem.* **84**, 1904-1908 (2014).
5. Shin, M. et al. Electrolyte optimization of alkaline aqueous redox flow battery using iron-2,2-bis(hydroxymethyl)-2,2′,2′-nitrilotriethanol complex as active material for anolyte. *Chem. Eng. J.* **453**, 139738 (2023).
6. Ferguson, A. et al. Linking iron(iii) carboxylates into high-nuclearity complexes by using Bis-tris. *Dalton Trans.* **6**, 731-733 (2008).
7. Jang, J. et al. Full-hexacyanometallate aqueous redox flow batteries exceeding 1.5 V in an aqueous solution. *Adv. Energy Mater.* **13**, 2300707 (2023).
8. Bae, CH. et al. Chromium redox couples for application to redox flow batteries. *Electrochim. Acta* **48**, 279-287 (2002).
9. Robb, BH. et al. Chelated chromium electrolyte enabling high-voltage aqueous flow batteries. *Joule* **3**, 2503-2512 (2019).
10. Bamgbopa, MO. et al. The potential of non-aqueous redox flow batteries as fast-charging capable energy storage solutions: demonstration with an iron-chromium acetylacetonate chemistry. *J. Mater. Chem. A* **5**, 13457-13468 (2017).
11. Ruan, W. et al. Designing Cr complexes for a neutral Fe-Cr redox flow battery. *Chem. Commun.* **56**, 3171-3174 (2020).
12. Wu, CD. et al. A bismuth-based electrocatalyst for the chromous-chromic couple in acid electrolytes. *J. Electrochem. Soc.* **133**, 2109 (1986).
13. Zhen, Y. et al. A high-performance all-iron non-aqueous redox flow battery. *J. Power Sources* **445**, 227331 (2020).

14. Shin, M. et al. All iron aqueous redox flow batteries using organometallic complexes consisting of iron and 3-[bis(2-hydroxyethyl)amino]-2-hydroxypropanesulfonic acid ligand and ferrocyanide as redox couple. *Chem. Eng. J.* **391**, 125631 (2020).
15. DeBruler, C. et al. Unprecedented capacity and stability of ammonium ferrocyanide catholyte in pH neutral aqueous redox flow batteries. *Joule* **3**, 149-163 (2019).
16. Noh, C. et al. Highly stable aqueous organometallic redox flow batteries using cobalt triisopropanolamine and iron triisopropanolamine complexes. *Chem. Eng. J.* **405**, 126966 (2021).
17. Lê, A. et al. Highly soluble Fe(III)-triethanolamine complex relevant for redox flow batteries. *Electrochim. Acta* **301**, 472-477 (2019).
18. Waters, SE. et al. Effect of chelation on iron-chromium redox flow batteries. *ACS Energy Lett*. **5**, 1758-1762 (2020).
