# Supplementary material for: Ligand engineering enables an alkaline iron-chromium redox flow battery
Source: Nat Commun. 2026 Sep 30;17:11240. doi: 10.1038/s41467-026-77810-8 (PMC13720190; doi:10.1038/s41467-026-77810-8)
Supplement: Supplementary file 2 [file 41467_2026_77810_MOESM2_ESM.pdf]

<u>**Description of Additional Supplementary Files**</u>

Supplementary Data 1. Atomic coordinates of the DFT-optimized Cr(III)(BIS-TRIS) model ($C_8H_{16}CrNO_6$). Four hydroxyl groups of the BIS-TRIS ligand are deprotonated, whereas one ligand hydroxyl group remains protonated. The model contains one coordinated hydroxo ligand and no aqua ligand. The total charge is −2, and the spin multiplicity is 2 (doublet).

Supplementary Data 2. Atomic coordinates of the DFT-optimized Cr(IV)(BIS-TRIS) model ($C_8H_{16}CrNO_6$). Four hydroxyl groups of the BIS-TRIS ligand are deprotonated, whereas one ligand hydroxyl group remains protonated. The model contains one coordinated hydroxo ligand and no aqua ligand. The total charge is −1, and the spin multiplicity is 1 (singlet).

Supplementary Data 3. Atomic coordinates of the DFT-optimized Cr(III)(TEA) model ($C_6H_{13}CrNO_4$). All three hydroxyl groups of the TEA ligand are deprotonated. The model contains one coordinated hydroxo ligand and no aqua ligand. The total charge is −1, and the spin multiplicity is 2 (doublet).

Supplementary Data 4. Atomic coordinates of the DFT-optimized Cr(IV)(TEA) model ($C_6H_{13}CrNO_4$). All three hydroxyl groups of the TEA ligand are deprotonated. The model contains one coordinated hydroxo ligand and no aqua ligand. The total charge is 0, and the spin multiplicity is 1 (singlet).

Supplementary Data 5. Atomic coordinates of the DFT-optimized Cr(III)$(H_2O)_6$ model ($CrH_{12}O_6$). The model contains six fully protonated aqua ligands and no hydroxo ligand; no deprotonation is applied. The total charge is +3, and the spin multiplicity is 2 (doublet).

Supplementary Data 6. Atomic coordinates of the DFT-optimized Cr(IV)$(H_2O)_6$ model ($CrH_{12}O_6$). The model contains six fully protonated aqua ligands and no hydroxo ligand; no deprotonation is applied. The total charge is +4, and the spin multiplicity is 1 (singlet).

Supplementary Data 7. Atomic coordinates of the DFT-optimized Cr(III)(DEA) model ($C_4H_{10}CrNO_3$). Both hydroxyl groups of the DEA ligand are deprotonated. The model contains one coordinated hydroxo ligand and no aqua ligand. The total charge is 0, and the spin multiplicity is 2 (doublet).

Supplementary Data 8. Atomic coordinates of the DFT-optimized Cr(IV)(DEA) model ($C_4H_{10}CrNO_3$). Both hydroxyl groups of the DEA ligand are deprotonated. The model contains one coordinated hydroxo ligand and no aqua ligand. The total charge is +1, and the spin multiplicity is 3 (triplet).
