# Supplementary material for: Ligand engineering enables an alkaline iron-chromium redox flow battery
Source: Nat Commun. 2026 Sep 30;17:11240. doi: 10.1038/s41467-026-77810-8 (PMC13720190; doi:10.1038/s41467-026-77810-8)
Supplement: Supplementary file 3 [file 41467_2026_77810_MOESM4_ESM.pdf]

nature portfolio

Peer Review File

Ligand engineering enables an alkaline iron-chromium redox flow battery

Corresponding Author: Professor Jiangjiang Duan

This file contains all reviewer reports in order by version, followed by all author rebuttals in order by version.

Version 0:

Reviewer comments:

Reviewer #1

(Remarks to the Author)
In this study, the authors suggest Cr(BIS-TRIS) as an advanced active material of catholyte for alkaline flow batteries. This is important study because the currently available catholyte candidates are very limited. Based on comprehensive characterization, the authors demonstrate that Cr(BIS-TRIS) exhibits superior electrochemical performance to $CrCl_3$. Furthermore, full-cell tests and subsequent analysis of capacity decay associated with reactions involving free ligands corroborate that the Cr (BIS-TRIS) can become an excellent catholyte candidate. However, there are still some issues to be addressed.
1. The authors determined the chemical structure of Cr(BIS-TRIS) using ESI-HRMS. Although dilution is typically required for ESI-HRMS analysis, OH^- still appears to be chelated in the reported spectra. To enhance the reliability of the data, the authors should provide more detailed experimental conditions for the ESI-HRMS measurements, including the dilution factor and the solvent used for dilution.
2. The solubility of Fe(BIS-TRIS) and Cr(BIS-TRIS) were determined using UV-Vis spectra of saturated solutions. However, the preparation method of these saturated solutions is not clearly described in this manuscript. Since the measured solubility depends on how saturation is implemented, a detailed description of the preparation procedure should be included in the text.
3. In Supplementary Table 6, the authors present raw material costs as a total cost of 17.02 \$/kWh. However, the sources of the raw material prices were not specified. The authors should provide appropriate references or sources for such a cost estimation.
4. In Supplementary Fig. 15, the CV curves of $CrCl_3$ and Cr(BIS-TRIS) were compared. The authors should specify the supporting electrolyte used for $CrCl_3$. While it was stated that 5 M KOH was used for 0.1 M Cr(BIS-TRIS), the corresponding information for $CrCl_3$ was not shown in the text.
5. Regarding Supplementary Fig. 16, the authors should clarify whether EIS measurements were performed at the open-circuit potential (OCP) or at a specific applied potential, as the EIS response can be influenced by the measurement potential. In addition, as noted above, the supporting electrolyte used for $CrCl_3$ should be explained in the text.
6. In Supplementary Fig. 22, VE and EE appear to be interchanged. Since EE is defined as the product of CE and VE, EE cannot exceed VE. Please correct this issue.
7. On page 10, the diffusion coefficient (D0) of Cr(BIS-TRIS) was reported as 5.279×10^{-6} cm2 s−1, while 6.93×10^{-6} cm2 s−1 was listed in Supplementary Table 5. This discrepancy should be resolved. Additionally, Supplementary Tables 4 and 5 used different notations for diffusion coefficients (DO, DR, D0). Please clarify the distinction between DO and D0, as both notations appear to refer to the same value. Consistent notation is essential for clarity.
8. In Supplementary Fig. 27, the polarity labeling should be corrected. Since Fe(BIS-TRIS) is played as the anolyte and Cr(BIS-TRIS) as the catholyte, the positions of the positive (+) and negative (−) signs should be exchanged.
9. On page 11, the final sentence states, "The proposed oxidation pathways of BIS-TRIS are shown in Fig. 5d." However, Fig. 5d presents UV-Vis spectra, while the proposed oxidation reactions of BIS-TRIS were shown in Fig. 5f. Please verify and correct the figure citation.
10. Although there were some flow battery related researches using Fe(BIS-TRIS) or Cr(BIS-TRIS) as electrolyte candidate that were previously published, the references included in this manuscript were very limited. The authors should find out more references for that, and please include them in the revised manuscript.

Reviewer #2

(Remarks to the Author)
The sluggish kinetics of Cr(II/III) are widely recognized as a critical bottleneck in Fe-Cr redox flow batteries (RFBs), and strategies to mitigate this limitation have remained a focal point of extensive research. In this work, the authors report an unconventional, highly reversible Cr(III/IV) redox couple enabled by a unique amino-polyhydroxy ligand. This effectively circumvents the kinetic impediments of the traditional Cr(II)/Cr(III) reaction. Besides, they cleverly ultilize the same ligand to develop an alkaline Fe-Cr RFB with a reversed anolyte-catholyte configuration. This design is particularly compelling as it completely sidesteps the persistent limitations of acidic Fe-Cr chemistry. Therefore, I support the publication of this work in Nature Communications after revision.

1. Why can only amino-polyhydroxy ligands stabilize iron and chromium ions in alkaline environments? What is the key difference between them and amino-polycarboxylic or amino-polyphosphate ligands?
2. The use of an identical ligand for both the anolyte and catholyte represents a highly ingenious design strategy. In alkaline Fe-Cr RFB, can the anolyte and catholyte be mixed for operation, as is commonly practiced in acidic Fe-Cr systems?
3. What about the chelation stability? The authors need to consider the issue of spontaneous decomposition of the organic ligands under long-term operating conditions, especially whether the free ligands in solution will undergo decomposition.
4. The authors attribute the reversible capacity fading to a mild side reaction between Cr(IV)(BIS-TRIS) and free BIS-TRIS ligands. Based on this mechanism, I am curious about how the system behaves in a capacity-unbalanced cell (e.g., with excess anolyte).
5. The authors provide the CE data of alkaline Fe-Cr RFBs at different ligand-metal ratios of the Cr(BIS-TRIS) catholyte. Detailed cycling data should also be included to further support the findings.
6. The scale format of the ESP maps in Figure 3f needs to be further standardized.
7. In Figure 5f, more compelling evidence should be provided for the oxidation product analysis of BIS-TRIS, such as ESI-HRMS data.
8. Is the system oxygen sensitive? How do the authors mitigate that?
9. Although the authors have provided a preliminary estimate of the electrolyte cost for the alkaline Fe-Cr RFB, this cost only accounts for raw material expenses and does not represent the full manufacturing cost. It is suggested that the authors should phrase this with greater precision. The manufacturing cost of the alkaline Fe-Cr RFB electrolyte is likely to be higher than that of VRFBs.

Reviewer #3

(Remarks to the Author)
Manuscript NCOMMS-25-96732 entitled “Ligand engineering enables a catholyte-anolyte reversed alkaline iron-chromium redox flow battery” by Yang and colleagues reports on a chromium-based positive electrolyte for application in alkaline aqueous redox flow batteries. The previously reported complex Fe(BIS-TRIS) is used as a negolyte and coupled with Cr(BIS-TRIS) as posolyte, which is presented as a "reversed" configuration, as Cr-complexes are more commonly employed on the negative side. Indeed, various Cr(II/III)-based negolytes have been described, and exploration of the Cr(III/IV) couple as a posolyte is, in principle, intriguing.
While the experimental work is carefully executed and the interpretations are largely convincing, the scope and generality of the advance remain limited. The newly introduced posolyte does not surpass established benchmarks such as ferrocyanide in any key metric other than solubility, and the resulting full-cell performance is far inferior to previous reports employing Fe/Cr-based active materials. As such, the work represents an interesting materials study but does not constitute a broadly relevant conceptual advance that would merit publication in Nature Communications. After appropriate revisions, the study is better suited for a more specialized energy materials or electrochemistry journal.
Below are some general remarks that may help the authors improve their work and communicate their approach more effectively:
- The "reversed" narrative is unnecessarily distracting, I suggest simply discussing Fe-negative, Cr-positive, so what if it is often the other way around.
- P1: network-scale implementation --> grid-scale
- P1: abundant availability --> abundance
- P1: transformative approach --> unnecessarily hyperbolic language
- P2: Why is it important to develop alkaline-tolerant metal-organics? To minimize HER? Would organics not make more sense in alkaline environments?
- P2: amino-polyhydroxy ligands can stabilize metal ions even under alkaline conditions --> the same applies to aminopolycarboxylates but you state that e.g., CrPDTA is less alkaline-tolerant than Cr(BIS-TRIS), how can you rationalize this? Especially, because you state on P4 that electron donating deprotonated hydroxyl groups are the key here. CrPDTA also has four such groups.
- P2: How do you explain a full cell voltage of 1.15 V, while the authors of this study https://www.sciencedirect.com/science/article/pii/S1385894722052172 achieve 1.43 V while using the same negolyte and ferrocyanide, which has an almost identical potential to Cr(BIS-TRIS)?
- P4: There is no coordinating water in Cr(BIS-TRIS) as for Fe(BIS-TRIS)? How come you detect Cr(BIS-TRIS)OH fragment in HRMS? A crystal structure of the material would be highly informative.
- P5: "facilitating facile electron transfer20,29,35" in addition to the cited references, and for the discussion of XPS data, this study may be of interest to the authors: https://chemistry-europe.onlinelibrary.wiley.com/doi/full/10.1002/batt.202500250
- Fig 2a-c: From the text it is not evident to me how these ligands/solutions were assessed. Furthermore, it is inappropriate to classify aminopolycarboxylates and aminopolyphosphonates as weak ligands.

- P6: Why does a narrower HOMO-LUMO gap enhance electron-transfer "propensity"?
- P7: "high electron density of deprotonated oxygen" --> again, same logic applies to EDTA, PDTA.
- P8: remarkable reversibility --> unnecessary hyperbolic language
- P8: Cr(BIS-TRIS) is described as highly reversible but the peak separation in CV is 229 mV, which is hardly highly reversible.
- P8: How do D0 and k0 highlight "alkaline stability"?
- P10: The description of evolving negolyte imbalance is nice.
- P10: Ligand-to-metal ratio studies show that free ligand reacts with the charged posolyte species, so having free ligand is a bad idea. Why not synthesize pure Cr(BIS-TRIS) and prepare the electrolyte from that without free ligand? This is a key concern I have with this study (and most metal complex RFB studies for that matter): Synthesizing the electrolyte in-situ yields a highly skewed picture. What happens to the chloride ions from CrCl3? How do they affect solubility and electrochemical properties?

To describe a new material I would expect synthesis of the pure compound, i.e. isolate KCr(BIS-TRIS), characterize it, and then prepare RFB electrolytes from it. This criticism applies to almost every paper out there regarding e.g., Fe(BIS-TRIS), Fe(TEA), Fe(DIPSO), Ce(DTPA) etc.
- Can you clarify if the active material is KCr(BIS-TRIS) or K2Cr(BIS-TRIS) for example?
- P11: Can you provide experiments without free ligand to prove that there is no irreversible chelate decomposition? How does this affect Coulombic efficiency and capacity retention? According to your results the problem should be resolved by not having any free ligand? What is the upper pH-limit of Cr(BIS-TRIS)?
- The authors should acknowledge some of the work by Shin et al. who previously described Fe(BIS-TRIS) in detail: https://onlinelibrary.wiley.com/doi/10.1002/er.7718, https://www.sciencedirect.com/science/article/pii/S1385894722052172, https://www.sciencedirect.com/science/article/pii/S1385894723034137

Version 1:

Reviewer comments:

Reviewer #3

(Remarks to the Author)
The authors did an excellent job addressing my questions and concerns and I support publication of the article in Nature Communications after the following minor comments have been resolved:
- Figure R1/R6 is helpful and should be included in the supporting information.
- Abstract: The authors should specify that the 68.07 Ah/L are per posolyte volume not per total volume of electrolyte.
- L63: PDTA is labelled "strong-field ligand" which is correct, but later in the manuscript it is positioned as (relatively) weak.
- L72: "alternatively, alkaline systems..." CrPDTA is also operated in alkaline environments (pH 7-10). Much less alkaline that pH 14 but technically still alkaline.
- Figure 2a-c: The authors should remove "moderate ligand" from a) and b) because these ligands are by all means still very strong and industry standard ligands, I think it would be misleading to label them as moderate. In c) the authors should remove "super" because with a peak separation of >200 mV this is, as correctly described in the text, quasi-reversible, not super reversible.
- L256: ohmic --> Ohmic
- L259: are you sure this is just the membrane resistance, not the total Ohmic resistance?
- L277: The authors should point out in the main text that the solubility values are obtained for mixed-cation systems.

Open Access This Peer Review File is licensed under a Creative Commons Attribution 4.0 International License, which permits use, sharing, adaptation, distribution and reproduction in any medium or format, as long as you give appropriate credit to the original author(s) and the source, provide a link to the Creative Commons license, and indicate if changes were made.
In cases where reviewers are anonymous, credit should be given to 'Anonymous Referee' and the source.
The images or other third party material in this Peer Review File are included in the article's Creative Commons license, unless indicated otherwise in a credit line to the material. If material is not included in the article's Creative Commons license and your intended use is not permitted by statutory regulation or exceeds the permitted use, you will need to obtain permission directly from the copyright holder.
To view a copy of this license, visit https://creativecommons.org/licenses/by/4.0/

List of Changes and Response to the Reviewers' comments

Dear Editors and Reviewers:

We would like to express our sincere thanks to the reviewers for the critical and careful review of the entire manuscript. Their constructive suggestions and comments are helpful and inspiring for us to reword some of the statements in the text to make it precise, clear and easy to read. Listed below are our point-by-point responses to their questions/concerns. All changes in the Manuscript are marked as red.

Response to Reviewers' comments

Reviewer #1:

General Comment:

In this study, the authors suggest Cr(BIS-TRIS) as an advanced active material of catholyte for alkaline flow batteries. This is important study because the currently available catholyte candidates are very limited. Based on comprehensive characterization, the authors demonstrate that Cr(BIS-TRIS) exhibits superior electrochemical performance to $CrCl_3$. Furthermore, full-cell tests and subsequent analysis of capacity decay associated with reactions involving free ligands corroborate that the Cr (BIS-TRIS) can become an excellent catholyte candidate. However, there are still some issues to be addressed.

Response: We sincerely thank the reviewer for the positive assessment of our work and for the valuable suggestions provided to improve our manuscript.

Comment 1-1: **The authors determined the chemical structure of Cr(BIS-TRIS) using ESI-HRMS. Although dilution is typically required for ESI-HRMS analysis, OH^- still appears to be chelated in the reported spectra. To enhance the reliability of the data, the authors should provide more detailed experimental conditions for the ESI-HRMS measurements, including the dilution factor and the solvent used for dilution.**

Response: We thank the reviewer for this constructive comment. Accordingly, detailed experimental conditions for the ESI-HRMS measurements, including the dilution factor and the solvent used for

dilution, have been added on Page 5 of the **Revised Supporting Information**. The revised text is as follows:

*"**Electrosprayionization high resolution mass spectroscopy (ESI-HRMS).** ESI-HRMS (Thermo Scientific Q Exactive) was used to confirm the chemical structures of chromium chelates. Mass spectra were obtained in positive ion mode. The Cr(BIS-TRIS) sample was diluted 10-fold with 0.1% aqueous ammonia prior to ESI-HRMS measurement."*

Comment 1-2: The solubility of Fe(BIS-TRIS) and Cr(BIS-TRIS) were determined using UV-Vis spectra of saturated solutions. However, the preparation method of these saturated solutions is not clearly described in this manuscript. Since the measured solubility depends on how saturation is implemented, a detailed description of the preparation procedure should be included in the text.

Response: We thank the reviewer for this important comment. A detailed description of the preparation procedure for the saturated Fe(BIS-TRIS) and Cr(BIS-TRIS) solutions has now been added on Page 3 of the **Revised Supporting Information**. Briefly, the saturated solutions were prepared using excess reagents in a minimal volume of solvent to ensure saturation, followed by equilibration at room temperature and removal of undissolved solids prior to UV-Vis measurements. The revised description is provided below.

"To obtain saturated solutions, $FeCl_3·6H_2O$ or $CrCl_3·6H_2O$ and BIS-TRIS at a molar ratio of 1 : 1.1 were added to a solution containing 5 M KOH and 5 M NaOH. The combination of KOH and NaOH was adopted to enhance the solubility of the metal chelates via a diverse ion effect. The metal concentration was gradually increased with a gradient of 0.1 M (1.5 M, 1.6 M, ...) until visible hydroxide precipitation was observed. The resulting turbid suspension was then stirred at room temperature for 24 h and subsequently filtered to obtain a clear saturated solution. For UV-Vis measurements, standard solutions of Fe(BIS-TRIS) and Cr(BIS-TRIS) with known concentrations were prepared to construct calibration curves based on the Beer-Lambert law. The maximum solubility of the metal chelates in the alkaline electrolyte was then determined from the absorbance-concentration relationship."

Comment 1-3: In Supplementary Table 6, the authors present raw material costs as a total cost of

17.02 $/kWh. However, the sources of the raw material prices were not specified. The authors should provide appropriate references or sources for such a cost estimation.

Response: We thank the reviewer for these very helpful comments and suggestions. The raw material prices were collected from Guidechem (https://china.guidechem.com/) and the source has now been clearly specified in **Supplementary Table 6**. The electrolyte cost estimation method follows the approach reported in Ref. 1 in the **Revised Supporting Information** (*Angew. Chem. Int. Ed.*, **2024**, 63, e202316593). The calculation procedure is detailed below:

*"**Calculation of raw material cost of the electrolyte:** The price of raw materials was obtained from Guidechem.com (https://china.guidechem.com/) and listed in **Supplementary Table 6**. We think that the cost of the electrolytes should include the cost of the redox pairs (both positive redox pairs and negative redox pairs) and the cost of supporting electrolytes, which is in line with the actual situation of large-scale applications. We calculated the price of redox pairs in terms of theoretical energy density to facilitate comparison with the results in other papers. The electrolyte cost was calculated by the following equation[1].*

$$C_e(\$/kWh) = \left[\Sigma c_x(mol/L) \times M_x(g/mol) \times P_x(\$/kg)\right] / (n \times c \times F \times V) \quad (5)$$

where C_e is the raw material cost of the electrolyte, meaning how much $ the total cost including redox pairs for 1 kWh. c_x is the concentration of electrolytes (mol/L), M_x is the molecular weight of substances (g/mol), and P_x means how much $ the electrolytes cost for 1 kg ($/kg). n is the number of working electrons per molecule, c is the concentration of electrolytes, F is Faraday's constant of 26.8 Ah/mol, V is the cell voltage. For a 0.5 M Fe/Cr(BIS-TRIS) RFB, C_e=17.02 ($/kWh)."

Supplementary Table 6. *The raw material prices were collected from publicly available market quotations on Guidechem (https://china.guidechem.com/).*

Material	Molecular weight (g/mol)	Price ($/kg)
$CrCl_3 \cdot 6H_2O$	266.48	0.41
$FeCl_3 \cdot 6H_2O$	270.3	0.35
BIS-TRIS	209.24	0.10
KOH	56.11	0.28

Comment 1-4: In Supplementary Fig. 15, the CV curves of $CrCl_3$ and Cr(BIS-TRIS) were compared. The authors should specify the supporting electrolyte used for $CrCl_3$. While it was stated that 5 M KOH was used for 0.1 M Cr(BIS-TRIS), the corresponding information for $CrCl_3$ was not shown in the text.

Response: We thank the reviewer for this careful observation. The CV measurement of $CrCl_3$ was conducted in a solution containing 0.1 M $CrCl_3$ with 1 M HCl as the supporting electrolyte. This information has now been added on Page 3 of the **Revised Supporting Information**. The relevant section has been revised as follows:

"CV measurements were conducted using an EC-Lab potentiostat (BioLogic, VMP-300). A three-electrode configuration was employed with a glassy carbon working electrode (4 mm diameter) and a platinum flake counter electrode. For Cr(BIS-TRIS) and Fe(BIS-TRIS) in alkaline media, an Hg/HgO reference electrode was used. For $CrCl_3$ in acidic media, an Ag/AgCl reference electrode was employed. The supporting electrolytes were 5 M KOH for the complex solutions (0.1 M) and 1 M HCl for the $CrCl_3$ solution (0.1 M)."

Comment 1-5: Regarding Supplementary Fig. 16, the authors should clarify whether EIS measurements were performed at the open-circuit potential (OCP) or at a specific applied potential, as the EIS response can be influenced by the measurement potential. In addition, as noted above, the supporting electrolyte used for $CrCl_3$ should be explained in the text.

Response: We thank the reviewer for this valuable comment. The EIS measurements were performed at the open-circuit potential (OCP). In addition, the experimental details of the EIS measurements and the supporting electrolyte used for $CrCl_3$ have now been explicitly specified on Page 3 of the **Revised Supporting Information**. The revised text is provided below:

"EIS measurements were performed on a glassy carbon electrode with an active area of 0.1256 cm^2 over a frequency range from 0.01 Hz to 10^6 Hz. The measurements were conducted at open-circuit voltage with an amplitude of 10 mV."

"CV measurements were conducted using an EC-Lab potentiostat (BioLogic, VMP-300). A three-electrode configuration was employed with a glassy carbon working electrode (4 mm diameter) and a

platinum flake counter electrode. For Cr(BIS-TRIS) and Fe(BIS-TRIS) in alkaline media, an Hg/HgO reference electrode was used. For $CrCl_3$ in acidic media, an Ag/AgCl reference electrode was employed. The supporting electrolytes were 5 M KOH for the complex solutions (0.1 M) and 1 M HCl for the $CrCl_3$ solution (0.1 M).”

Comment 1-6: In Supplementary Fig. 22, VE and EE appear to be interchanged. Since EE is defined as the product of CE and VE, EE cannot exceed VE. Please correct this issue.

Response: We thank the reviewer for pointing out this mistake. The VE and EE labels in Supplementary Fig. 22 have now been corrected in the **Revised Supporting Information**. The revised figure is provided below:

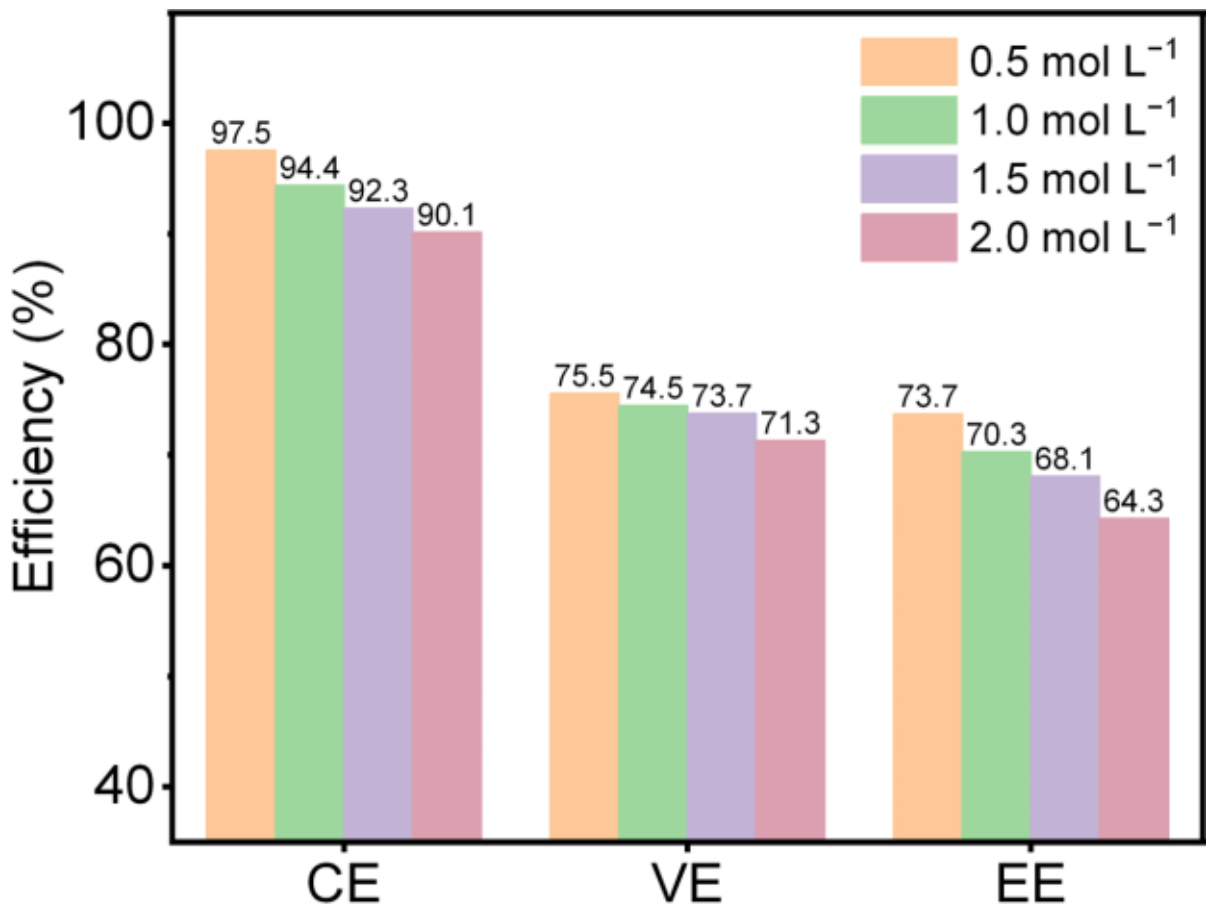

Supplementary Fig. 25. *Coulombic, voltage, and energy efficiencies of the alkaline Fe-Cr RFBs using different concentrations of electrolytes at 100 mA cm^{-2}.*

Comment 1-7: . On page 10, the diffusion coefficient (D0) of Cr(BIS-TRIS) was reported as 5.279 × 10^{-6} cm^2 s^{-1}, while 6.93 × 10^{-6} cm^2 s^{-1} was listed in Supplementary Table 5. This discrepancy should be resolved. Additionally, Supplementary Tables 4 and 5 used different notations for diffusion coefficients (DO, DR, D0). Please clarify the distinction between DO and D0, as both notations appear to refer to the same value. Consistent notation is essential for clarity .

Response: We thank the reviewer for this valuable comment. The diffusion coefficient values and the corresponding notations have been corrected and standardized. The revised text and tables are shown below.

*"Cr(BIS-TRIS) exhibits D_O of 6.93 × 10^{-6} cm^2 s^{-1} and k_O of 6.85×10^{-3} cm s^{-1}, representing a 1000-fold enhancement over uncatalyzed Cr(II/III) in $CrCl_3$ (**Supplementary Table 4**)."*

Supplementary Table 4. *Diffusion Coefficients and standard rate constants of Fe(BIS-TRIS) and Cr(BIS-TRIS) chelates.*

Species	D_O (cm^2 s^{-1})	D_R (cm^2 s^{-1})	K_O (cm s^{-1})	K_R (cm s^{-1})
Cr(BIS-TRIS)	6.93×10^{-6}	6.62×10^{-6}	6.85×10^{-3}	1.87×10^{-3}
Fe(BIS-TRIS)	3.03×10^{-6}	4.07×10^{-6}	2.79×10^{-3}	4.92×10^{-3}

Supplementary Table 5. *The electrochemical properties of Cr-based active materials.*

Species	D_O (cm^2 s^{-1})	K_O (cm s^{-1})	Reference
Cr(BIS-TRIS)	6.93×10^{-6}	6.85×10^{-3}	This work
$K_3[Cr(CN)_6]$	7.16×10^{-6}	6.03×10^{-3}	14
Cr(EDTA)	8.1×10^{-6}	-	15
Cr(PDTA)	6.2×10^{-6}	1.7×10^{-4}	16
$Cr(acac)_3$	4.91×10^{-6}	1.52×10^{-3}	17
$CrCl_3$	-	$<10^{-6}$	18
$[Cr(f\text{-}DPA)_2]Br$	-	10^{-3}	18
$CrCl_3$ (with Bi catalyst)	6.2×10^{-6}	1.35×10^{-3}	19

Comment 1-8: **. In Supplementary Fig. 27, the polarity labeling should be corrected. Since Fe(BIS-TRIS) is played as the anolyte and Cr(BIS-TRIS) as the catholyte, the positions of the positive (+) and negative (−) signs should be exchanged.**

Response: We thank the reviewer for this observation. The polarity signs in this figure have been corrected. The revised figure is provided below:

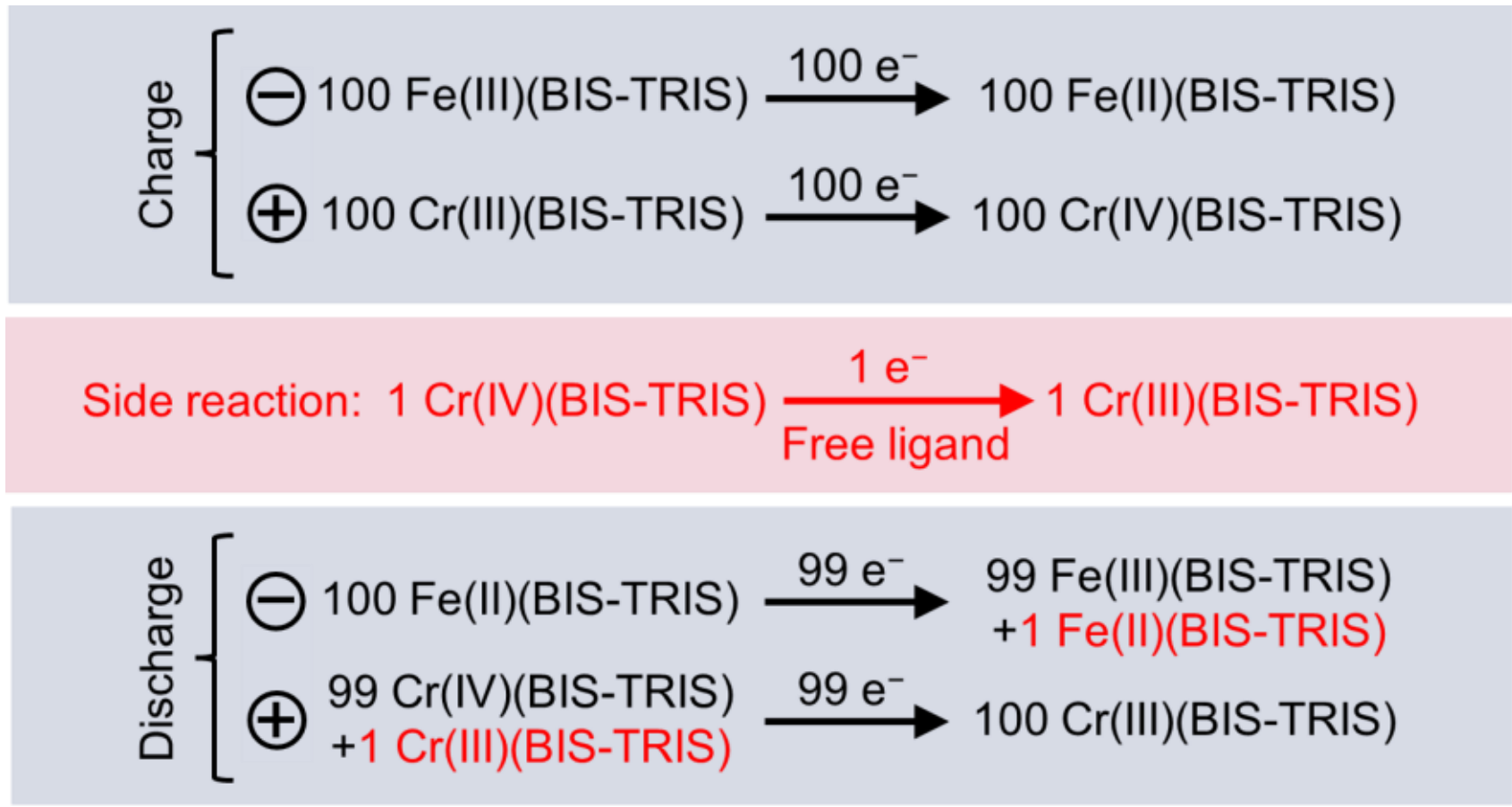

Supplementary Fig. 30. *The specific explanation for capacity degradation due to the side reaction between Cr(IV)(BIS-TRIS) and free BIS-TRIS ligands.*

Comment 1-9: **. On page 11, the final sentence states, "The proposed oxidation pathways of BIS-TRIS are shown in Fig. 5d." However, Fig. 5d presents UV-Vis spectra, while the proposed oxidation reactions of BIS-TRIS were shown in Fig. 5f. Please verify and correct the figure citation.**

Response: We appreciate the reviewer for very carefully reviewing our manuscript. The figure citation in the text has been corrected. The proposed oxidation pathways of BIS-TRIS are now correctly referenced as **Fig. 5**f in the **Revised Manuscript**.

Comment 1-10: **Although there were some flow battery related researches using Fe(BIS-TRIS) or Cr(BIS-TRIS) as electrolyte candidate that were previously published, the references included in this manuscript were very limited. The authors should find out more references for that, and please include them in the revised manuscript.**

Response: We thank the reviewer for this helpful suggestion. Cr(BIS-TRIS) is first proposed here as a catholyte candidate. Fe(BIS-TRIS) has previously been reported as an anolyte in alkaline all-iron RFBs. Relevant references have now been added to the **Revised Manuscript** and are listed below.

"49. Shin, M. et al. Electrolyte optimization of alkaline aqueous redox flow battery using iron-2,2-bis(hydroxymethyl)-2',2'-nitrilotriethanol complex as active material for anolyte. Chem. Eng. J. ***453****, 139738 (2023).*

50. Shin, M. et al. Spectroscopic and electrochemical analyses elucidating capacity degradation mechanism of iron-ligand complex and air in all iron aqueous redox flow batteries. Chem. Eng. J. ***471****, 144682 (2023).*

51. Shin, M. et al. Aqueous redox flow battery using iron 2,2-bis(hydroxymethyl)-2,2',2'-nitrilotriethanol complex and ferrocyanide as newly developed redox couple. Int. J. Energy Res. ***46****, 8175-8185 (2022)."*

Reviewer #2:

General Comment:

The sluggish kinetics of Cr(II/III) are widely recognized as a critical bottleneck in Fe-Cr redox flow batteries (RFBs), and strategies to mitigate this limitation have remained a focal point of extensive research. In this work, the authors report an unconventional, highly reversible Cr(III/IV) redox couple enabled by a unique amino-polyhydroxy ligand. This effectively circumvents the kinetic impediments of the traditional Cr(II)/Cr(III) reaction. Besides, they cleverly ultilize the same ligand to develop an alkaline Fe-Cr RFB with a reversed anolyte-catholyte configuration. This design is particularly compelling as it completely sidesteps the persistent limitations of acidic Fe-Cr chemistry. Therefore, I support the publication of this work in Nature Communications after revision.

Response: We sincerely thank the reviewer for the positive assessment of our work and for the valuable suggestions provided to improve our manuscript.

Comment 2-1: **Why can only amino-polyhydroxy ligands stabilize iron and chromium ions in alkaline environments? What is the key difference between them and amino-polycarboxylic or amino-polyphosphate ligands?**

Response: Thank you for your insightful comment. The fundamental distinction among these ligand classes arises from the local electronic structure of the coordinating oxygen atoms, particularly the spatial distribution of negative charge and its influence on σ-donor strength.

Under strongly alkaline conditions, the hydroxyl groups in amino-polyhydroxy ligands (e.g., BIS-TRIS) are deprotonated to form alkoxide species ($–O^{-}$). In this configuration, the negative charge is highly localized on a single oxygen atom and does not significantly participate in π-resonance delocalization. As a result, the coordinating oxygen exhibits relatively high local electron density and enhanced σ-donor capability. For high charge-density hard metal centers such as Fe(II/III) and Cr(II/III), stronger σ donation increases the M-O bond strength and improves the ligand's ability to compete with OH^{-} for coordination. This effectively suppresses hydrolysis and hydroxide precipitation, thereby maintaining soluble complex formation under strongly alkaline conditions (**Fig. R1**a).

In contrast, in amino-polycarboxylate ligands (e.g., EDTA), the negative charge of the carboxylate group ($–COO^{-}$) is delocalized over two oxygen atoms through π resonance. Although such

delocalization stabilizes the overall structure, it reduces the local charge density at each individual oxygen donor, thereby attenuating the σ-donor strength of a single coordination site. Consequently, its competitive ability against OH^- for binding to the metal center is comparatively limited in strongly alkaline media (**Fig. R1**b).

For amino-polyphosphonate ligands (e.g., ATMP), the phosphonate group ($–PO_3^{2-}$) is typically highly deprotonated under alkaline conditions and carries a high overall formal charge. However, this negative charge is distributed across multiple P-O bonds and is modulated by the strong polarization character of the P-O linkage. Despite the high overall anionic charge, the local electron density at any individual oxygen atom is not as concentrated as in alkoxide donors, and the effective σ-donor strength per coordinating oxygen is moderated by this charge distribution (**Fig. R1**c).

Therefore, from the perspective of local electronic structure, alkoxide-type oxygen donors exhibit a higher degree of charge localization and stronger σ-donor ability, whereas carboxylate and phosphonate oxygen donors experience charge delocalization or dispersion effects that lower the local electron density at each individual oxygen site. This difference directly influences their ability to compete with OH^- for metal coordination and constitutes an important electronic factor underlying the superior capability of amino-polyhydroxy ligands to suppress metal hydrolysis and maintain soluble complex formation in strongly alkaline environments. Consequently, amino-polyhydroxy ligands can effectively stabilize iron and chromium ions in alkaline solutions without precipitation, whereas amino-polycarboxylate and amino-polyphosphonate ligands are more susceptible to hydroxide substitution at the coordination sites, leading to the formation of insoluble metal hydroxides.

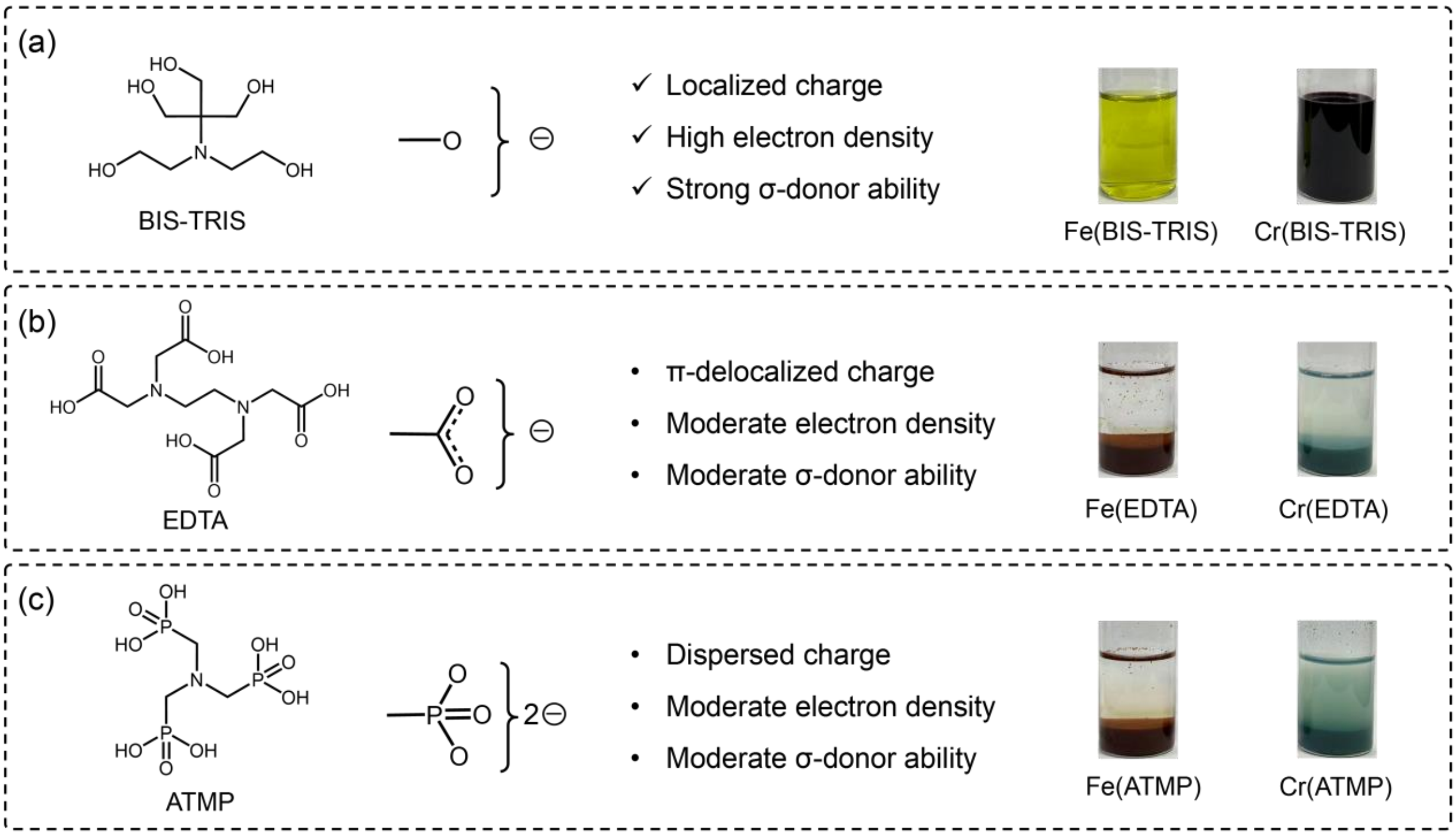

Fig. R1. Comparison of ligand electronic characteristics and electrolyte stability in alkaline systems (pH=14). Electronic structure of coordinating atoms in (a) an amino-polyhydroxy ligand (BIS-TRIS), (b) an amino-polycarboxylate ligand (EDTA), and (c) an amino-polyphosphonate ligand (ATMP), and their corresponding stability toward metal ions under alkaline conditions.

Comment 2-2: The use of an identical ligand for both the anolyte and catholyte represents a highly ingenious design strategy. In alkaline Fe-Cr RFB, can the anolyte and catholyte be mixed for operation, as is commonly practiced in acidic Fe-Cr systems?

Response: We sincerely thank the reviewer for this insightful comment. As suggested, in conventional acidic Fe-Cr systems, the use of identical supporting electrolytes allows mixing of the anolyte and catholyte for operation. Similarly, in our alkaline Fe-Cr RFB, the anolyte and catholyte can also be mixed due to the use of the same chelating ligand in both electrolytes.

Benefiting from this symmetric design, we directly prepared a mixed electrolyte and employed it for full-cell testing. The mixed-electrolyte cell was evaluated at 80% state of charge (SOC) and exhibited stable cycling performance over 200 cycles, delivering a CE of 99.0% and a EE of 72.9% at 100 mA cm^{-2} (**Supplementary Fig. 22**).

In addition, we have incorporated a description of the mixed-electrolyte cell performance in the **Revised Manuscript** and **Revised Supporting Information**, as detailed below.

"To synthesize a 0.5 M mixed electrolyte, 2.13 g $CrCl_3·6H_2O$ and 3.24 g $FeCl_3·6H_2O$ was dissolved into 20 mL of DIW and stirred for 30 min. Subsequently, 4.5 g of BIS-TRIS was added to the mixture and stirred for 30 min. Then, 6.73 g KOH was dissolved into the mixture, stirring for 24 hours to obtain the mixed electrolyte. Finally, the volume of the solution was adjusted to 40 mL with DIW."

*"Owing to the use of the same ligand in both electrolytes, the anolyte and catholyte of the alkaline Fe-Cr RFB can be prepared as a mixed solution, as is commonly practiced in acidic Fe-Cr systems. The resulting mixed-electrolyte system exhibits competitive performance, delivering a CE of 99.0%, a EE of 72.9%, and stable cycling over 200 cycles (**Supplementary Fig. 22**)."*

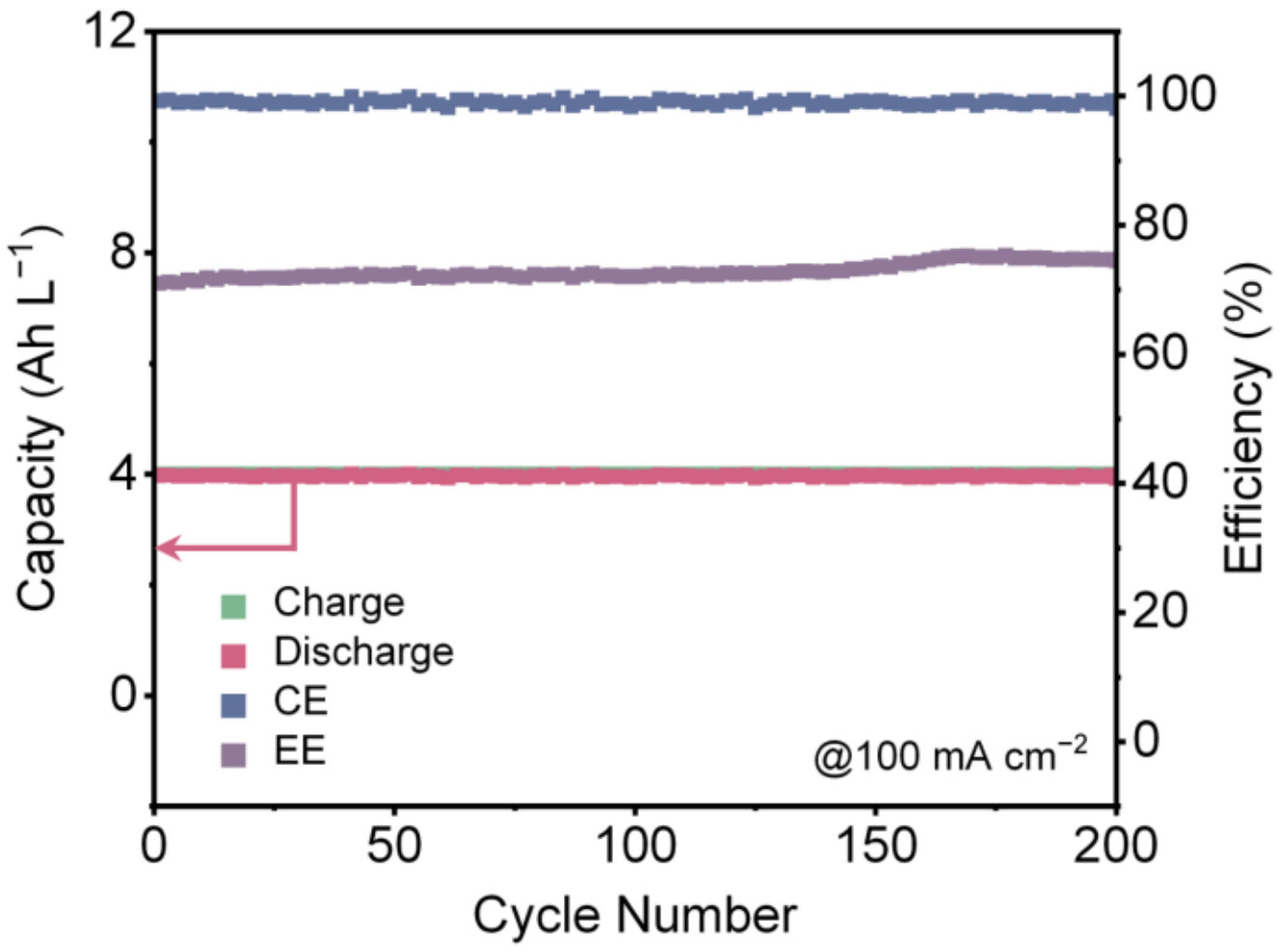

Supplementary Fig. 22. *Cycling performance of the alkaline Fe-Cr RFB with a mixed electrolyte at 80% SOC and 100 mA cm^{-2}.*

Comment 2-3: What about the chelation stability? The authors need to consider the issue of spontaneous decomposition of the organic ligands under long-term operating conditions, especially whether the free ligands in solution will undergo decomposition.

Response: We sincerely thank the reviewer for this careful and important comment. To evaluate the stability of the Cr(BIS-TRIS) chelate during operation, we analyzed the catholyte after 500 charge-discharge cycles (83 h). Cyclic voltammetry and UV-Vis absorption spectra were recorded and compared with those of the fresh electrolyte (**Supplementary Fig. 21**). No noticeable changes in redox behavior or spectral features were observed, indicating that the coordinated Cr(BIS-TRIS) species remained stable during the tested cycling period, with no detectable decomposition under the present

conditions.

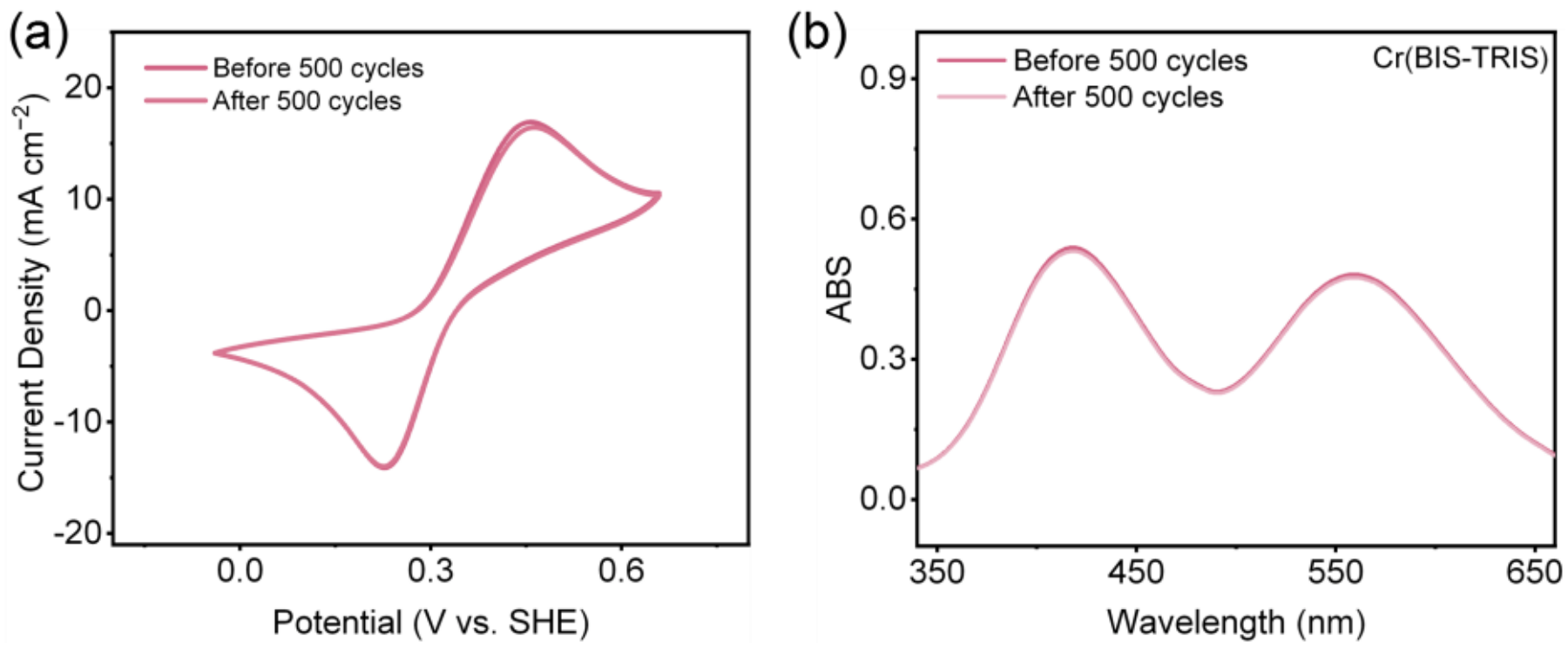

Supplementary Fig. 21. *CV and UV-Vis spectra of the Cr(BIS-TRIS) catholyte with a ligand-to-metal ratio of 1.1:1 before and after 500 charge-discharge cycles.*

At the same time, it should be noted that the Cr(BIS-TRIS) chelate is formed through a reversible coordination equilibrium:

$$Cr^{3+} + \text{BIS-TRIS} + 5OH^{-} \rightleftharpoons [Cr(\text{BIS-TRIS})(OH)]^{2-} + 4H_2O \quad (1)$$

Because this coordination process is reversible, the long-term stability of the chelate is inherently linked to the availability of free BIS-TRIS ligand in solution. Although no detectable decomposition of the coordinated Cr(BIS-TRIS) species was observed within 500 cycles under the tested conditions, gradual consumption of free ligand during prolonged operation may shift the coordination equilibrium and eventually compromise chelate stability. This effect becomes more evident when the amount of free ligand is insufficient. For example, in the Cr(BIS-TRIS) catholyte with a ligand-to-metal ratio of 1:1, the capacity can only be restored to ~90% upon oxygen introduction after 100 cycles, indicating that a small degree of irreversible chelate decomposition occurs under this condition, as also evidenced by the optical image of the cycled electrolyte (**Fig. R2**). Therefore, periodic replenishment of the ligand may be necessary to maintain chelate stability during prolonged operation.

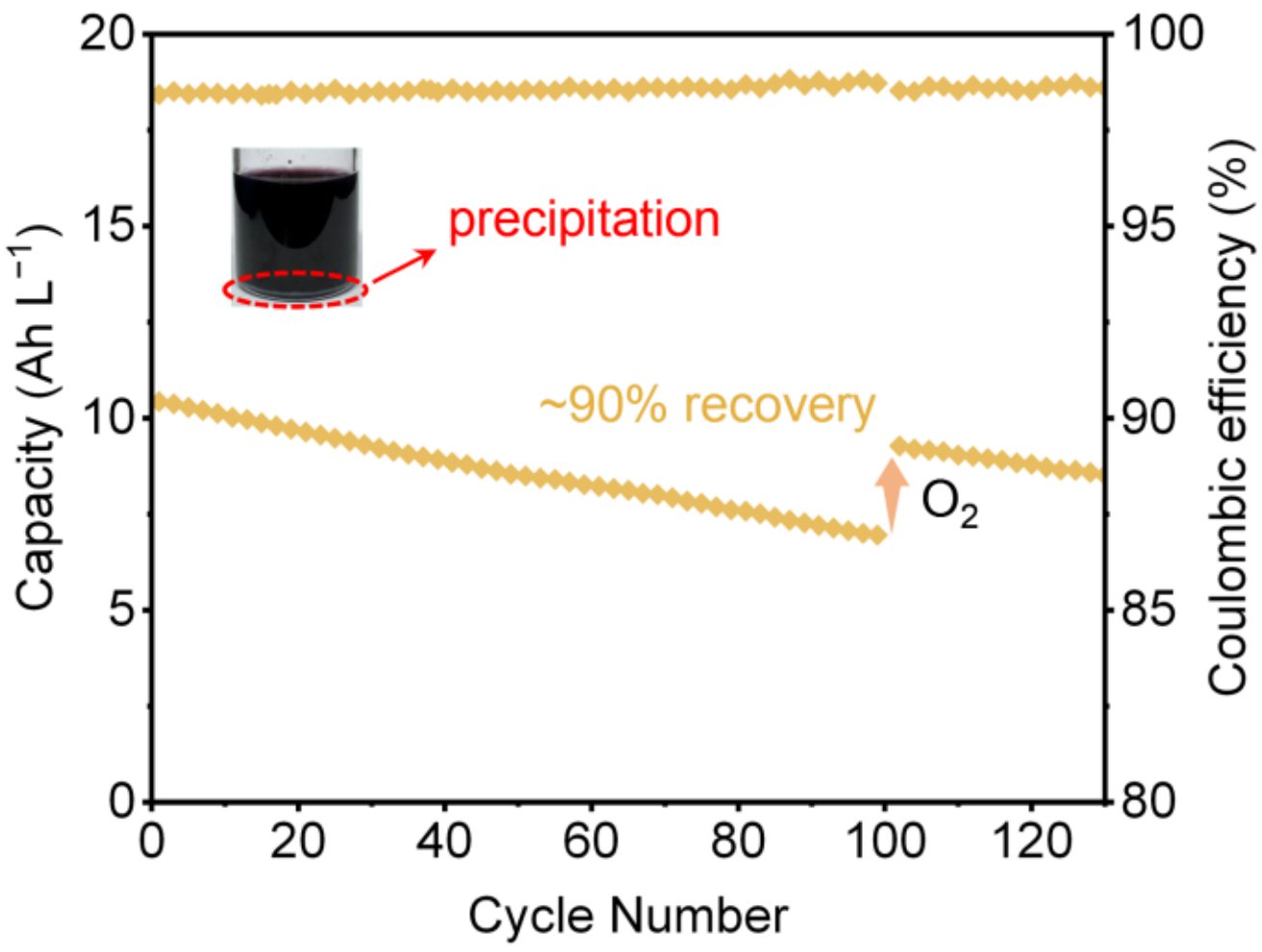

Fig. R2. *Extended cycling performance and capacity recovery of the 1:1 system upon oxygen exposure of the anolyte, together with an optical image of the catholyte of the 1:1 system after cycling.*

Comment 2-4: The authors attribute the reversible capacity fading to a mild side reaction between Cr(IV)(BIS-TRIS) and free BIS-TRIS ligands. Based on this mechanism, I am curious about how the system behaves in a capacity-unbalanced cell (e.g., with excess anolyte).

Response: We thank the reviewer for this valuable comment. According to the proposed fading mechanism, the side reaction in the catholyte can lead to a charge imbalance between the anolyte and catholyte, resulting in the accumulation of Fe(II)(BIS-TRIS) and a corresponding deficiency of Fe(III)(BIS-TRIS) in the anolyte, which causes the observed capacity decay (**Fig. R3**a). Therefore, when excess Fe(III)(BIS-TRIS) is supplied to the anolyte, the cell capacity is expected to remain stable (**Fig. R3**b). To test this, we constructed a capacity-unbalanced cell with a 50% excess anolyte (Fe(III)(BIS-TRIS)). As shown in **Fig. 4**f, the cell operated stably over 500 cycles without any observable capacity decay, providing further experimental validation of the proposed fading mechanism.

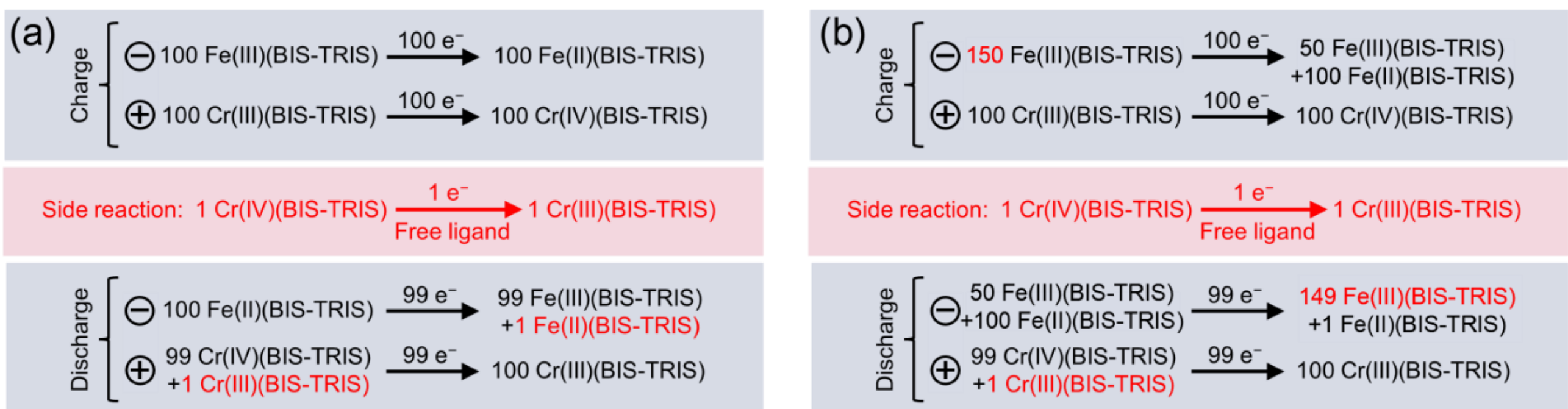

Fig. R3. Effect of side reaction on the charge-discharge behavior of the alkaline Fe-Cr RFB under (a) capacity-balanced and (b) anolyte-excess conditions.

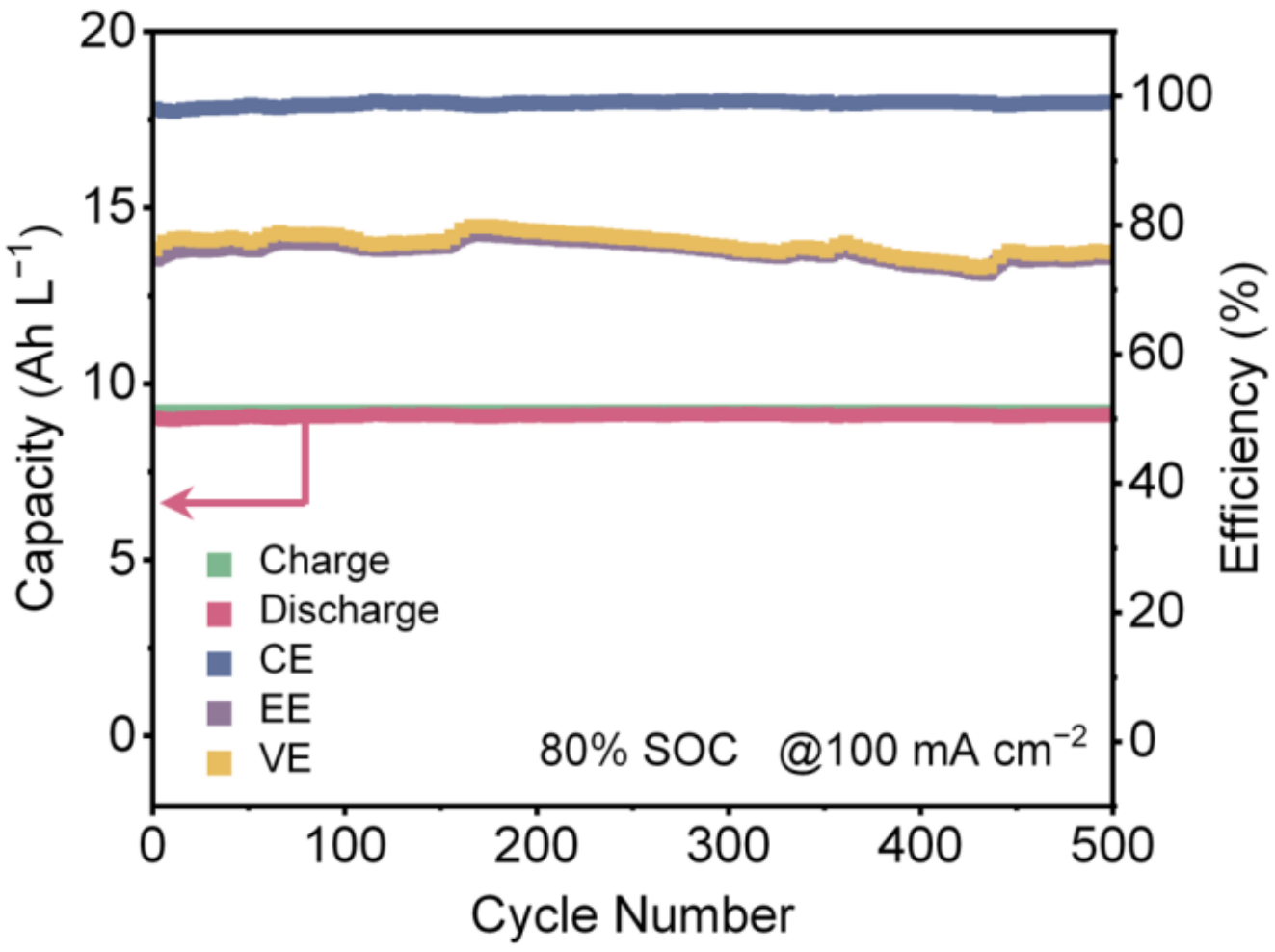

Fig.4. *(f) Cycling performance of the alkaline Fe-Cr RFB with 50% excess anolyte at 80% SOC.*

Comment 2-5: **The authors provide the CE data of alkaline Fe-Cr RFBs at different ligand-metal ratios of the Cr(BIS-TRIS) catholyte. Detailed cycling data should also be included to further support the findings.**

Response: We sincerely thank the reviewer for this helpful suggestion. In response, we have added the 50-cycle Coulombic efficiency and discharge-capacity data for alkaline Fe-Cr RFBs with different ligand-to-metal ratios in the Cr(BIS-TRIS) catholyte. The revised content and corresponding figure are shown below.

*"Further analysis revealed that the CE is highly sensitive to the concentration of free ligand in the catholyte. As the ligand-to-metal ratio increased from 1.1:1 to 1.2:1 and 1.3:1, the average CE decreased from 98.3% to 97.7% and 96.9%, respectively (**Supplementary Fig. 29**), indicating that uncoordinated BIS-TRIS molecules participate in parasitic side reactions with the oxidized Cr species."*

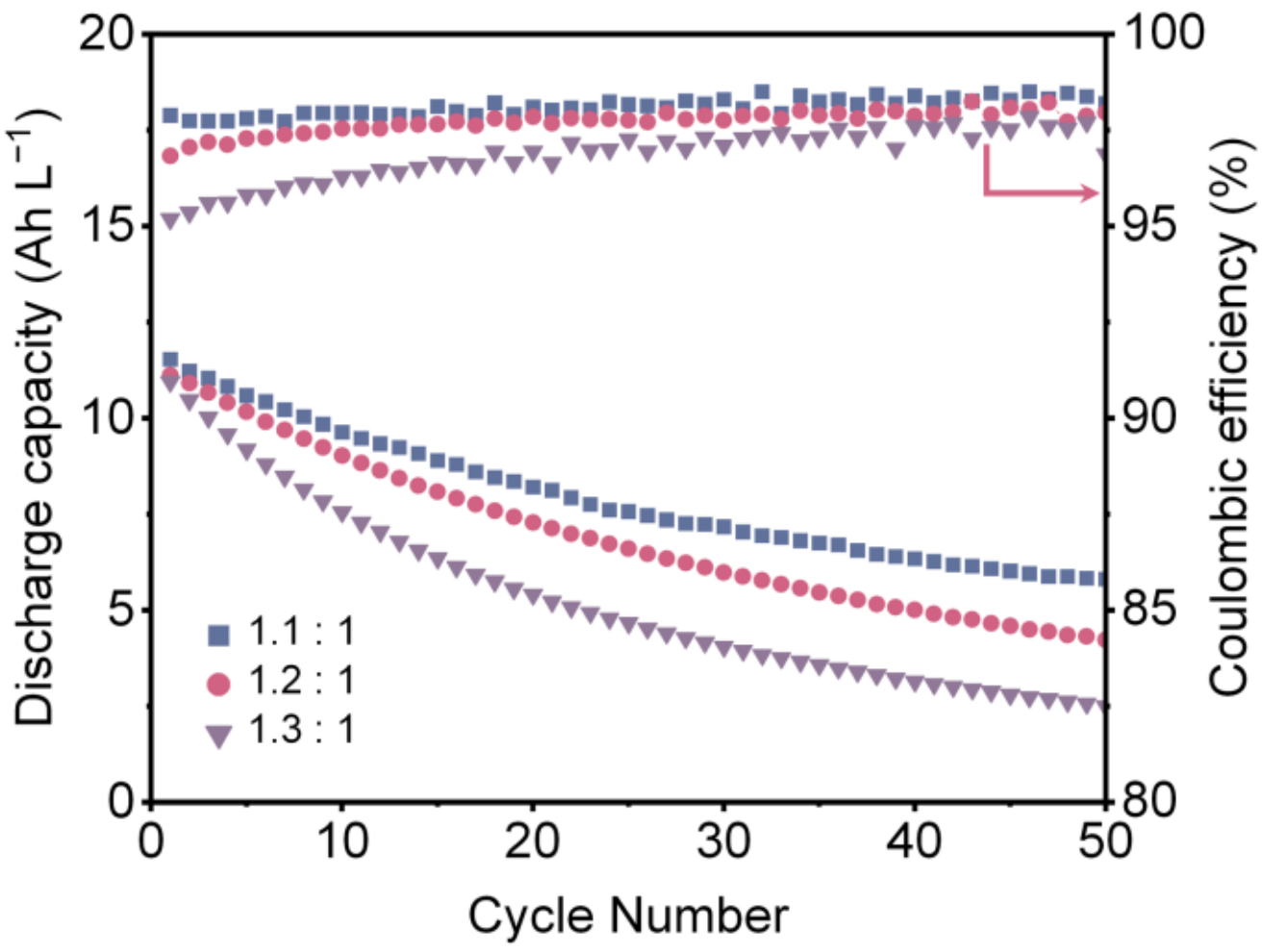

Supplementary Fig. 29. *Cycling performance of a 0.5 M alkaline Fe-Cr RFB with varying ligand-to-metal ratios in the Cr(BIS-TRIS) catholyte at 100% SOC and 100 mA cm^{-2}.*

Comment 2-6: The scale format of the ESP maps in Figure 3f needs to be further standardized.

Response: We thank the reviewer for this careful observation. The scale format of the ESP maps in **Fig. 3**f has been standardized in the **Revised Manuscript**, as shown below.

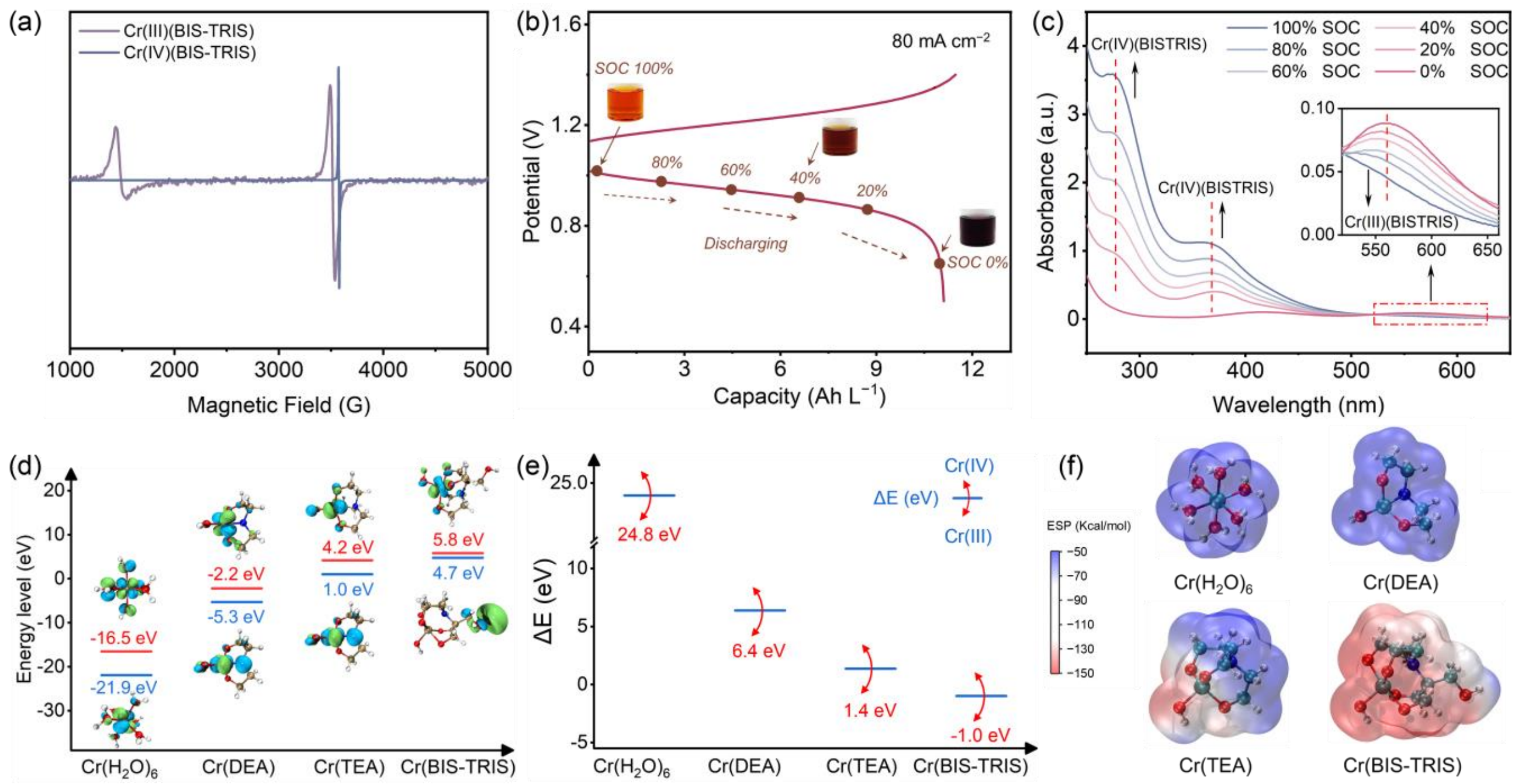

Fig. 3 | Electrochemical mechanism and theoretical calculation of the Cr(BIS-TRIS) catholyte. *(a) EPR spectra of charged-state Cr(IV)(BIS-TRIS) and discharged-state Cr(III)(BIS-TRIS). (b) A typical voltage profile of Cr(BIS-TRIS) and the corresponding solution photographs at different SOC (0%,*

40%, and 100%). (c) UV-Vis spectra of Cr(BIS-TRIS) electrolytes at different SOC (0%, 20%, 40%, 60%, 80%, and 100%). (d, e) (d) Calculated HOMO and LUMO energy levels and (e) energy changes of different Cr species during Cr(III/IV) redox reactions. (f) The ESP maps of different Cr species.

Comment 2-7: In Figure 5f, more compelling evidence should be provided for the oxidation product analysis of BIS-TRIS, such as ESI-HRMS data.

Response: We thank the reviewer for this valuable suggestion. In response, we performed ESI-HRMS measurements on the catholyte after 500 cycles to provide additional evidence for the oxidation product analysis of BIS-TRIS. As shown in **Fig. R4**a, we propose two possible oxidation pathways for the free BIS-TRIS ligand and the corresponding oxidation product structures. The two possible oxidation products were calculated to have *m/z* values of 225.12 and 223.11, respectively, and corresponding peaks were observed in the ESI-HRMS spectra (**Fig. R4**b). These results provide further support for the proposed oxidation product structures.

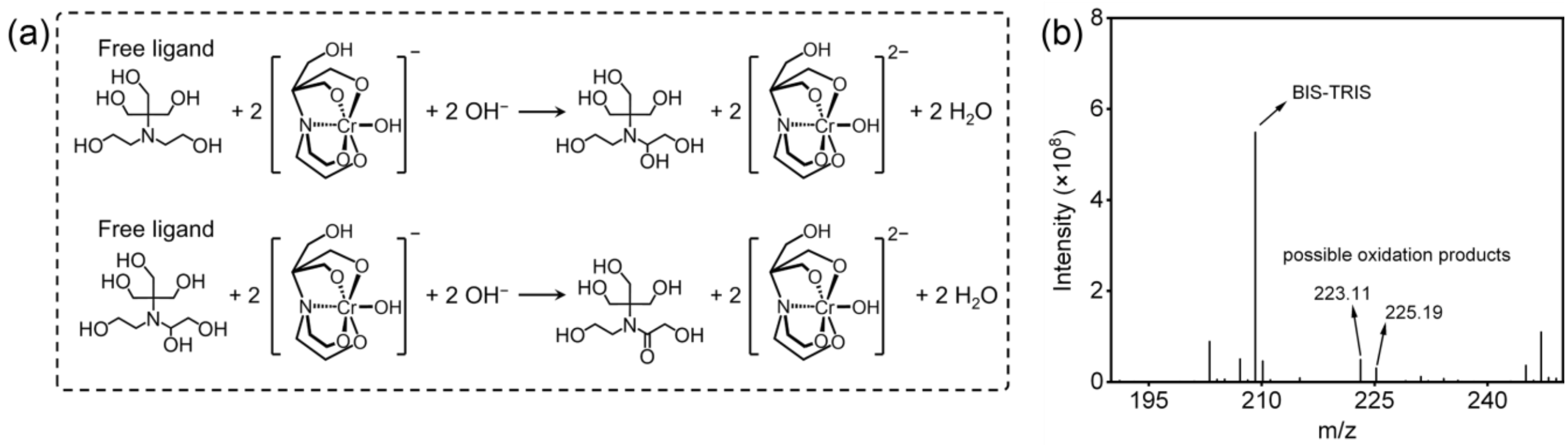

Fig. R4. *(a) Possible oxidation reactions of the free BIS-TRIS ligand and the corresponding oxidation product structures. (b) ESI-HRMS spectra of the Cr(BIS-TRIS) catholyte after cycling tests.*

Comment 2-8: Is the system oxygen sensitive? How do the authors mitigate that?

Response: We thank the reviewer for this question. In the alkaline Fe-Cr RFB system, only the anolyte is oxygen-sensitive. Specifically, the charged-state Fe(II)(BIS-TRIS) species can be oxidized by oxygen to Fe(III)(BIS-TRIS), leading to capacity loss (*Chem. Eng. J.*, **2023**, 471, 144682). To mitigate this side reaction, the anolyte reservoir was purged with nitrogen for 10 minutes prior to cell operation to remove oxygen.

Comment 2-9: **. Although the authors have provided a preliminary estimate of the electrolyte cost for the alkaline Fe-Cr RFB, this cost only accounts for raw material expenses and does not represent the full manufacturing cost. It is suggested that the authors should phrase this with greater precision. The manufacturing cost of the alkaline Fe-Cr RFB electrolyte is likely to be higher than that of VRFBs.**

Response: We sincerely thank the reviewer for this valuable comment. To improve clarity and precision, we have revised the manuscript by replacing "electrolyte cost" with "raw material cost of the electrolyte."

Reviewer #3:

General Comment:

Manuscript NCOMMS-25-96732 entitled "Ligand engineering enables a catholyte-anolyte reversed alkaline iron-chromium redox flow battery" by Yang and colleagues reports on a chromium-based positive electrolyte for application in alkaline aqueous redox flow batteries. The previously reported complex Fe(BIS-TRIS) is used as a negolyte and coupled with Cr(BIS-TRIS) as posolyte, which is presented as a "reversed" configuration, as Cr-complexes are more commonly employed on the negative side. Indeed, various Cr(II/III)-based negolytes have been described, and exploration of the Cr(III/IV) couple as a posolyte is, in principle, intriguing.

While the experimental work is carefully executed and the interpretations are largely convincing, the scope and generality of the advance remain limited. The newly introduced posolyte does not surpass established benchmarks such as ferrocyanide in any key metric other than solubility, and the resulting full-cell performance is far inferior to previous reports employing Fe/Cr-based active materials. As such, the work represents an interesting materials study but does not constitute a broadly relevant conceptual advance that would merit publication in Nature Communications. After appropriate revisions, the study is better suited for a more specialized energy materials or electrochemistry journal.

Response: We sincerely thank the reviewer for the assessment of our work and for the valuable suggestions provided to improve our manuscript. In the following, we clarify both the comparison between Cr(BIS-TRIS) and $Fe(CN)_6$ catholytes and the apparent discrepancy between the full-cell performance of our alkaline Fe-Cr RFB and previously reported Fe-Cr systems.

(1) Response regarding the comparison between Cr(BIS-TRIS) and $Fe(CN)_6$

An ideal catholyte should simultaneously provide high solubility, fast electrochemical kinetics, robust chemical stability, and low cost. We agree that $Fe(CN)_6$ catholytes remain important benchmarks because of their favorable redox reversibility and kinetics. However, their application in strongly alkaline systems is fundamentally constrained by limited solubility and poor chemical stability.

In contrast, Cr(BIS-TRIS) was deliberately designed as an advanced alkaline catholyte. In addition to exhibiting favorable electrochemical kinetics comparable to those of $Fe(CN)_6$, Cr(BIS-

TRIS) demonstrates significantly enhanced solubility and stability under strongly alkaline conditions. A concise comparison is summarized below.

- **Solubility:** The solubility of redox-active materials in the supporting electrolyte is a key parameter that determines the achievable capacity of RFBs. However, the energy density of $Fe(CN)_6$-based RFBs remains limited by the relatively low solubility of $Fe(CN)_6$. For example, the solubilities of $Na_4[Fe(CN)_6]$ and $K_4[Fe(CN)_6]$ in water are only 0.56 M and 0.76 M, respectively. To address this limitation, considerable efforts have been devoted to improving solubility through cation or ligand engineering, for example by using $(NH_4)_4[Fe(CN)_6]$, $Li_4[Fe(CN)_6]$, and $Na_4[Fe(Dcbpy)_2CN_2]$ (*Nat. Energy* **2021**, 6, 873–881; *Angew. Chem. Int. Ed.* **2023**, 62, e202304667; *Joule* **2019**, 3, 149–163). Although these studies have demonstrated effective strategies for enhancing solubility, the corresponding high-solubility ferrocyanide derivatives typically require more complicated preparation procedures. For example, $Li_4[Fe(CN)_6]$ and $(NH_4)_4[Fe(CN)_6]$ are obtained through resin-based ion-exchange column processing, followed by purification and composition verification, while $Na_4[Fe(Dcbpy)_2CN_2]$ requires a multistep coordination synthesis with careful pH control. Such procedures increase operational complexity and limit scalability. By contrast, the Cr(BIS-TRIS) electrolyte in this work can be prepared in situ by simple mixing, while still exhibiting an intrinsic solubility exceeding 2.0 M, nearly three times that of conventional $Fe(CN)_6$ systems.
- **Cost:** Based on publicly available raw material prices (https://china.guidechem.com/), the estimated costs of $K_4[Fe(CN)_6]$ and $Na_4[Fe(CN)_6]$ are approximately 0.66 and 0.34 \$ mol^{-1}, respectively. Solubility-enhancing strategies based on derivatives such as $Li_4[Fe(CN)_6]$ can further increase the cost substantially because of the additional processing and synthetic complexity involved. These costs are all higher than the combined cost of $CrCl_3$ and BIS-TRIS (~0.13 \$ mol^{-1}). This comparison suggests that Cr(BIS-TRIS) may offer a more favorable cost profile for large-scale applications.
- **Stability:** In terms of stability, $Fe(CN)_6$ faces considerable stability challenges in alkaline environments. Specifically, it undergoes a slow but irreversible CN^-/OH^- ligand exchange reaction, which leads to chemical decomposition and the formation of $Fe(OH)_3$ (*Nano Energy* **2017**, 42, 215–221; *Adv. Energy Mater.* **2023**, 13, 2203762). In contrast, Cr(BIS-TRIS) was specifically designed as a robust alkaline catholyte, and its chemical and electrochemical stability

under high-pH conditions has been systematically demonstrated in this study, with no observable decomposition during prolonged cycling.

- **Electrochemical kinetics:** $Fe(CN)_6$ has long served as a benchmark due to its favorable redox reversibility and kinetics. In this work, Cr(BIS-TRIS) exhibits a k of 6.85 × 10^{-6} cm s^{-1}, which is comparable to that of $Fe(CN)_6$ (5.89 × 10^{-6} cm s^{-1}). Consistently, full-cell measurements show similar energy efficiencies for Fe(BIS-TRIS)/Cr(BIS-TRIS) RFB and Fe(BIS-TRIS)/$Fe(CN)_6$ RFB, reaching 78.6% and 78.3% at 80 mA cm^{-2}, respectively (*Chem. Eng. J* **2023**, 453, 139738). These results indicate that Cr(BIS-TRIS) and $Fe(CN)_6$ exhibit comparable electrochemical kinetics.

(2) Response regarding the comparison between alkaline Fe-Cr RFB and previous Fe-Cr RFB reports

The Fe-Cr RFB has been extensively investigated for decades because of the low cost and earth abundance of both active elements. However, previously reported Fe-Cr systems are persistently limited by hydrogen evolution reaction (HER). In acidic Fe-Cr RFBs, the Cr(II/III) redox reaction suffers from sluggish Cr^{2+}/Cr^{3+} redox kinetics and severe HER, often requiring catalysts and elevated temperature to achieve acceptable performance (*Adv. Mater.* **2025**, 37, 2502094; *Adv. Sci.* **2025**, 13, e12148). In near-neutral Fe-Cr RFBs, Cr chelates based on the same Cr(II/III) chemistry are also used as the anolyte. However, the low redox potential of these Cr chelates, such as Cr(II/III)(PDTA) at −1.10 V vs. SHE, falls below the electrochemical stability window of water, making HER thermodynamically unavoidable even under near-neutral conditions (*Joule* **2019**, 3, 2503–2512). Moreover, the resulting HER further increases the local pH beyond the stability window of the Cr chelates, leading to irreversible chelate decomposition.

By contrast, the alkaline Fe-Cr RFB offers fundamental advantages in both reaction kinetics and HER suppression. It employs an unconventional Cr(III/IV) redox couple at +0.34 V vs. SHE, which circumvents the Jahn-Teller effects that limit traditional Cr(II/III) chemistry and thereby enables rapid electron-transfer kinetics. In addition, HER is intrinsically disfavored in alkaline media, and both redox couples lie within the electrochemical stability window of water. Therefore, the alkaline Fe-Cr system addresses the central limitations that have long constrained acidic and near-neutral Fe-Cr chemistries.

Regarding the reviewer's concern that the full-cell performance appears inferior to previously reported Fe-Cr RFBs, we believe that this impression mainly arises from differences in cell configuration and testing protocols. In this work, the alkaline Fe-Cr RFB was evaluated using a symmetric cell configuration with balanced electrolyte volumes and cycling up to 100% state of charge (SOC). This rigorous testing protocol was designed to probe the intrinsic performance limits and reveal potential degradation pathways under realistic and demanding conditions. By comparison, previous Fe-Cr RFB studies often employed asymmetric configurations, with the counter-electrolyte used in excess, or cycled under limited SOC (e.g., 80%), which may artificially extend apparent cycling stability but obscure intrinsic capacity decay. For example, in the state-of-the-art Fe-Cr system, such as the $Fe(CN)_6$/Cr(PDTA) RFB, cycling tests are conducted with $Fe(CN)_6$ at twice the concentration of Cr(PDTA) and under a limited 80% SOC (*Joule* **2019**, 3, 2503–2512). While such strategies can extend apparent cycling stability, they may obscure intrinsic capacity decay and degradation pathways.

To enable a more direct comparison, we re-evaluated our alkaline Fe-Cr RFB under similar conditions. As shown in **Fig. R5**, when operated at 80% SOC with a 50% excess of anolyte (Fe(BIS-TRIS)), the alkaline Fe-Cr RFB delivered stable cycling over 500 cycles with no observable capacity decay, while maintaining a Coulombic efficiency of 99.0% and an energy efficiency of 75.8%. The improved cycling stability is mainly attributed to the avoidance of deep charging, which slows parasitic side reactions and prolongs cell lifetime. Under these comparable conditions, the performance of our alkaline Fe-Cr RFB is clearly competitive with, and in cycling stability exceeds, that of previously reported state-of-the-art Fe-Cr systems (*Joule* **2019**, 3, 2503−2512; *Adv. Energy Mater.* **2023**, 13, 2300707; *ACS Energy Lett.* **2020**, 5, 1758−1762). Furthermore, both the anolyte and catholyte in the alkaline Fe-Cr RFB exhibit solubilities exceeding 2 M, significantly surpassing those of current state-of-the-art Fe-Cr systems.

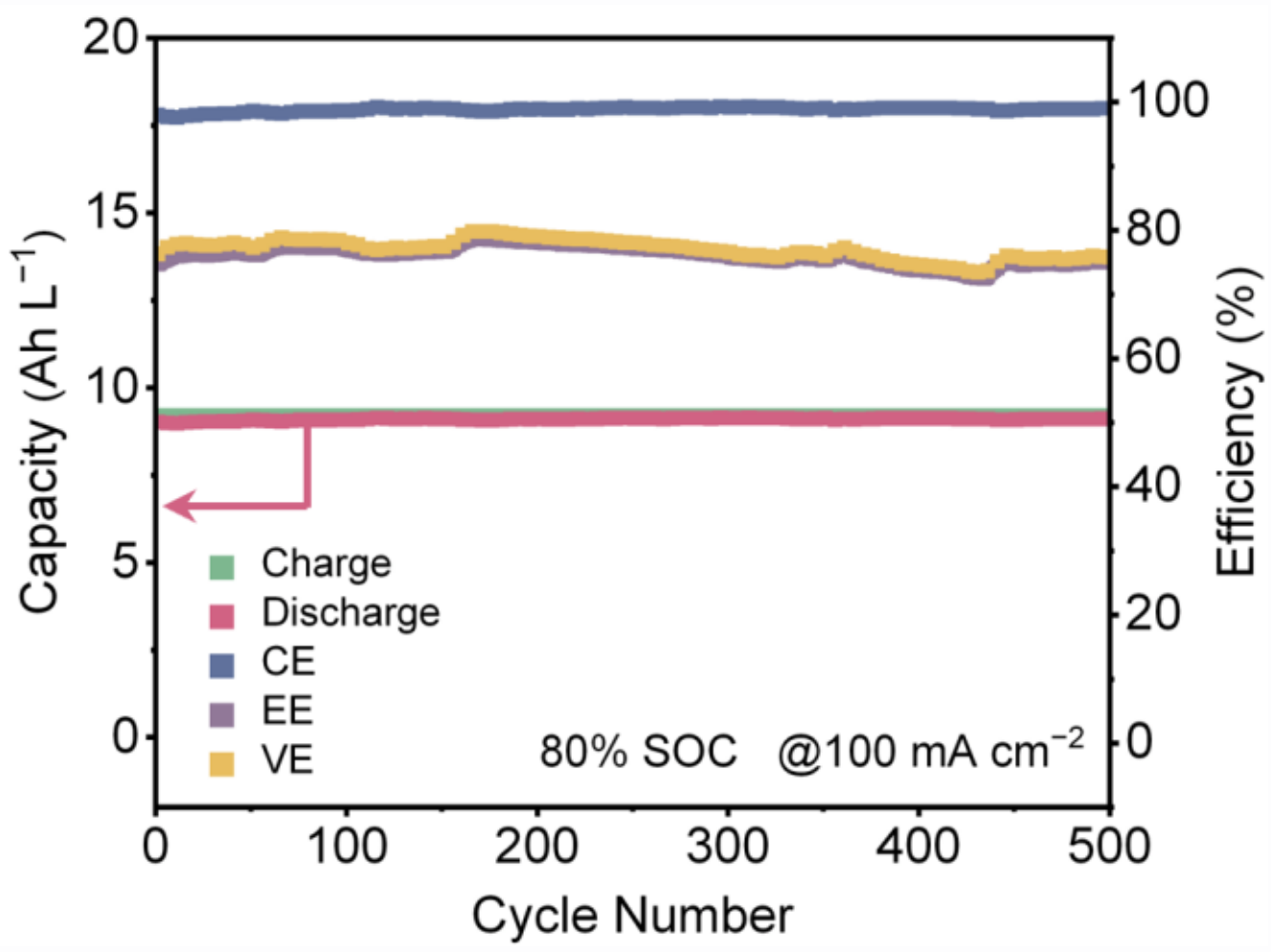

Fig. R5. Cycling performance of the alkaline Fe-Cr RFB with 50% excess analyte at 80% SOC and 100 mA cm^{-2}.

Comment 3-1: The "reversed" narrative is unnecessarily distracting, I suggest simply discussing Fe-negative, Cr-positive, so what if it is often the other way around.

Response: We thank the reviewer for this constructive comment. In the **Revised Manuscript**, we have removed the "reversed" description. The revised title and abstract are provided below.

"Ligand engineering enables an alkaline iron-chromium redox flow battery"

"Abstract: Iron-chromium (Fe-Cr) redox flow batteries (RFBs) are promising for long-duration energy storage owing to their safety, low cost, and environmental compatibility. However, their widespread implementation has been impeded for decades by sluggish Cr(II/III) redox kinetics and the parasitic hydrogen evolution reaction (HER) in Cr-based anolytes. Here, we overcome this persistent limitation by re-engineering Fe-Cr redox chemistry through a chelation-driven molecular design strategy. Employing the strong-field ligand bis(hydroxymethyl)-2,2',2''-nitrilotriethanol (BIS-TRIS), we reshape the electronic configurations of Fe and Cr centers and rationally tune their redox potentials, enabling an alkaline Fe-Cr RFB with inherently rapid redox kinetics and effective HER suppression. BIS-TRIS stabilizes an octahedral hexacoordinate Cr(BIS-TRIS) chelate and unlocks an unconventional Cr(III/IV) redox couple at +0.34 V vs. SHE, circumventing the Jahn-Teller effects that plague traditional Cr(II/III) chemistry and thereby accelerating electron-transfer kinetics. Paired with a Fe(BIS-TRIS) anolyte employing the same ligand, the full cell operates at 1.15 V within the aqueous stability window and sustains stable cycling over 500 cycles with a Coulombic efficiency of 99.0% and

an energy efficiency of 75.8% at 100 mA cm^{-2} (80% of state-of-charge). Combined with a low projected raw material cost (\$17.02 kWh^{-1}) and a high theoretical capacity (68.07 Ah L^{-1}), this work establishes a scalable and cost-effective chelation strategy for revitalizing Fe-Cr RFBs for grid-scale, long-duration energy storage."

Comment 3-2: P1: network-scale implementation --> grid-scale, P1: abundant availability --> abundance, P1: transformative approach --> unnecessarily hyperbolic language.

Response: We thank the reviewer for the careful reading of our manuscript and for these helpful suggestions. The manuscript has been revised accordingly. Specifically, "network-scale implementation" has been changed to "grid-scale", "abundant availability" has been replaced with "abundance", and "transformative approach" has been revised to "promising strategy".

Comment 3-3: P2: Why is it important to develop alkaline-tolerant metal-organics? To minimize HER? Would organics not make more sense in alkaline environments?

Response: We thank the reviewer for raising this important question. The development of alkaline-tolerant metal chelates is important because it enables Fe-Cr redox chemistry to operate under strongly alkaline conditions, where the hydrogen evolution reaction (HER) is intrinsically less favorable. In conventional acidic and near-neutral Fe-Cr RFBs, HER remains a persistent and difficult-to-resolve problem. Therefore, moving Fe-Cr chemistry into alkaline media provides a promising route to intrinsically mitigate HER.

However, this strategy is only viable if the metal-active species remain stable in strongly alkaline solution. This is a major challenge because most metal ions and metal complexes tend to undergo hydroxide substitution or ligand dissociation at high pH, eventually leading to the formation of insoluble metal hydroxide precipitates. Such instability is commonly observed in metal complexes with amino-polycarboxylate or amino-polyphosphonate ligands. Therefore, the central challenge in developing alkaline Fe-Cr RFBs lies in maintaining the coordination stability of metal chelates under strongly alkaline conditions.

Encouragingly, strong-field amino-polyhydroxy ligands can overcome this limitation. Under strongly alkaline conditions, the hydroxyl groups are deprotonated to form alkoxide species ($–O^{-}$),

which exhibit stronger σ-donor ability than hydroxide, carboxylate, and phosphonate oxygen donors. As a result, these alkoxide donors reinforce the metal-ligand interaction and help preserve the coordination stability of the Cr chelate in strongly alkaline solution. A more detailed comparison of these three types of oxygen donors is provided in our response to **Comment 3-4**.

Therefore, the development of alkaline-tolerant metal chelates is essential for realizing a practical alkaline Fe-Cr RFB chemistry that combines intrinsic HER suppression with stable metal coordination. We have also revised this part of the manuscript accordingly. The revised text is provided below.

"Alternatively, alkaline systems provide a thermodynamically favorable pathway to suppress HER. In addition, alkaline electrolytes are substantially less corrosive, enabling the use of cost-effective materials and improving system durability. However, developing stable metal-active species under alkaline conditions remains challenging, as most metal ions and their complexes tend to form insoluble hydroxide precipitates. Encouragingly, amino-polyhydroxy ligands exhibit exceptional capabilities in stabilizing metal ions even under strongly alkaline conditions, owing to their multidentate coordination and the high local electron density of alkoxide donors [28-30]. These features strengthen metal-ligand interactions and suppress hydroxide-induced dissociation, making amino-polyhydroxy ligands promising candidates for constructing stable alkaline RFB systems."

Comment 3-4: **P2: amino-polyhydroxy ligands can stabilize metal ions even under alkaline conditions --> the same applies to aminopolycarboxylates but you state that e.g., CrPDTA is less alkaline-tolerant than Cr(BIS-TRIS), how can you rationalize this? Especially, because you state on P4 that electron donating deprotonated hydroxyl groups are the key here. CrPDTA also has four such groups.**

Response: We thank the reviewer for this insightful comment. Amino-polycarboxylate ligands can indeed form stable metal chelates under near-neutral conditions; however, under strongly alkaline conditions, their coordination sites are readily displaced by hydroxide ions, leading to the formation of insoluble metal hydroxides. In contrast, amino-polyhydroxy ligands can effectively stabilize metal ions even in strongly alkaline media.

We would like to clarify that the key distinction does not lie merely in the presence of deprotonated oxygen donors, but in the local electronic structure of the coordinating oxygen atoms.

Specifically, the critical difference is whether the negative charge is localized on a single oxygen atom or delocalized over multiple atoms. Although both ligand classes contain deprotonated oxygen atoms, these oxygen donors are electronically inequivalent.

Under strongly alkaline conditions, the hydroxyl groups in amino-polyhydroxy ligands (e.g., BIS-TRIS) are deprotonated to form alkoxide species ($–O^{–}$). In these alkoxide donors, the negative charge is highly localized on a single oxygen atom and does not significantly participate in π-resonance delocalization. As a result, each coordinating oxygen possesses high local electron density and strong σ-donor capability. For hard, high charge-density metal centers such as Fe^{3+} and Cr^{3+}, this strong σ donation enhances metal-ligand bond strength and improves the ability of the ligand to compete with $OH^{–}$ for coordination, thereby suppressing hydrolysis and hydroxide precipitation under strongly alkaline conditions (**Fig. R6**a).

In contrast, in amino-polycarboxylate ligands (e.g., EDTA), the negative charge of each carboxylate group ($–COO^{–}$) is delocalized over two oxygen atoms through π resonance. Although these groups are also deprotonated, the local electron density at each individual oxygen donor is reduced by this delocalization, which weakens the effective σ-donor strength of a single coordination site. Consequently, carboxylate oxygen donors are less competitive against $OH^{–}$ in strongly alkaline media, making the corresponding metal complexes more susceptible to hydroxide substitution and precipitation (**Fig. R6**b). Similarly, for amino-polyphosphonate ligands (e.g., ATMP), although the phosphonate group ($–PO_3^{2–}$) carries a high overall negative charge, this charge is distributed across multiple P-O bonds and modulated by the strong polarization of the P-O linkage. As a result, the local electron density at each coordinating oxygen remains less concentrated than in alkoxide donors, leading to weaker effective σ-donor strength and reduced competitiveness against $OH^{–}$ (**Fig. R6**c).

Therefore, from the perspective of local electronic structure, alkoxide-type oxygen donors exhibit a higher degree of charge localization and stronger σ-donor ability, whereas carboxylate and phosphonate oxygen donors experience charge delocalization or dispersion effects that lower the local electron density at each individual oxygen site. This difference directly governs their ability to compete with $OH^{–}$ for metal coordination and underlies the markedly improved stability of Fe and Cr complexes in strongly alkaline environments. Consequently, amino-polyhydroxy ligands can effectively stabilize iron and chromium ions in alkaline solutions without precipitation, whereas amino-polycarboxylate and amino-polyphosphonate ligands are more susceptible to hydroxide substitution at the coordination

sites, leading to the formation of insoluble metal hydroxides.

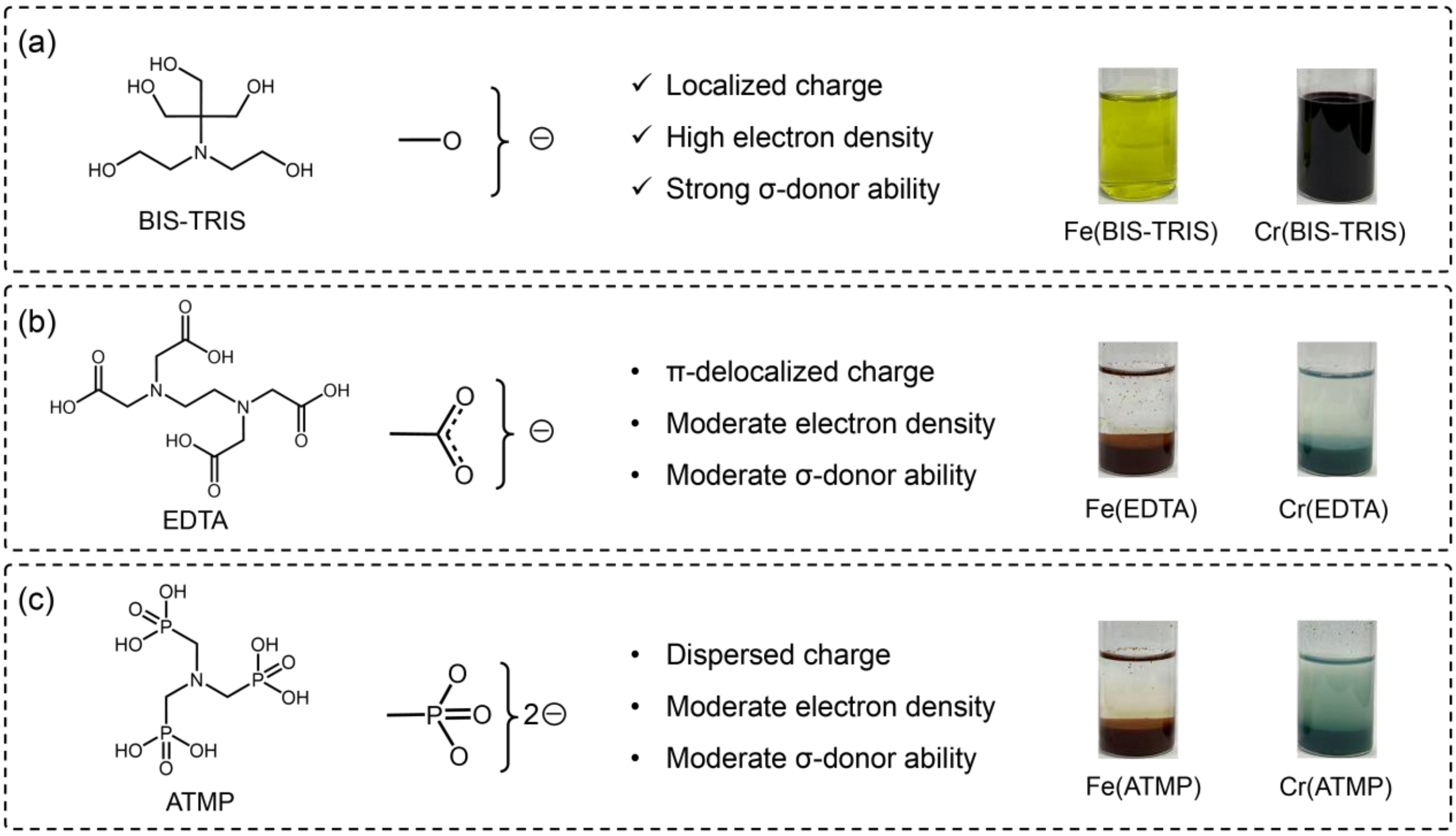

Fig. R6. *Comparison of ligand electronic characteristics and electrolyte stability in alkaline systems. Electronic structure of coordinating atoms in (a) an amino-polyhydroxy ligand (BIS-TRIS), (b) an amino-polycarboxylate ligand (EDTA), and (c) an amino-polyphosphonate ligand (ATMP), together with optical images of their corresponding metal chelates in alkaline media (pH = 14).*

To further clarify these differences, we have revised the corresponding discussion on Page 6 of the **Revised Manuscript**. The revised text is as follows:

*"We first evaluated the ability of different classes of ligands to stabilize Cr ions under strongly alkaline conditions (pH = 14). Among the three ligand classes, amino-polycarboxylate and amino-polyphosphonate ligands exhibited poor coordination stability, leading to the formation of Cr hydroxide precipitates in alkaline media. This instability arises because carboxylate and phosphonate oxygen donors undergo charge delocalization or dispersion, which reduces the local electron density at individual oxygen sites. Consequently, hydroxide ions can competitively displace the donor atoms, leading to metal-ligand bond cleavage, ligand dissociation, and eventual precipitation of Cr hydroxides (**Fig. 2d**, **Supplementary Figs. 2,3,5,6**). Therefore, although these ligands can form stable chelates with metal ions under near-neutral conditions, they are unsuitable for alkaline RFB applications. In contrast, amino-polyhydroxy ligands (e.g., diethanolamine (DEA), triethanolamine*

*(TEA), and BIS-TRIS) displayed significantly enhanced coordination stability under identical alkaline conditions (**Fig. 2d**, **Supplementary Fig. 4**). In strongly basic media, the hydroxyl groups of these ligands are deprotonated to form alkoxide species (–O⁻), in which the negative charge is localized on a single oxygen atom[19,20,31]. This charge localization increases the local electron density and strengthens the σ-donor capability of the coordinating oxygen, thereby reinforcing metal-ligand bonding and maintaining the structural integrity of the Cr chelates in strongly alkaline solution."*

Comment 3-5: **P2: How do you explain a full cell voltage of 1.15 V, while the authors of this study https://www.sciencedirect.com/science/article/pii/S1385894722052172 achieve 1.43 V while using the same negolyte and ferrocyanide, which has an almost identical potential to Cr(BIS-TRIS)?**

Response: We thank the reviewer for this valuable comment. The difference in full-cell voltage mainly arises from the different redox potentials of the catholytes. As shown in **Fig. R7**, the redox potential of the $Fe(CN)_6^{3-}/Fe(CN)_6^{4-}$ couple was measured to be approximately 0.54 V vs. SHE in strongly alkaline solution, whereas the Cr(BIS-TRIS) redox couple exhibits a lower potential of 0.34 V vs. SHE. Therefore, the full-cell voltage of the Fe(BIS-TRIS)/$Fe(CN)_6$ system is expected to be higher than that of the Fe(BIS-TRIS)/Cr(BIS-TRIS) system. In addition, the Fe(BIS-TRIS) anolyte in the literature report was prepared under different electrolyte conditions, including a different KOH concentration and a different ligand-to-metal ratio. Specifically, the reported anolyte was prepared in 4.5 M KOH with a ligand-to-metal ratio of 1.5:1, whereas our anolyte was prepared in 1.5 M KOH and 1.5 M NaOH with a ligand-to-metal ratio of 1.1:1. Such differences in anolyte preparation and supporting electrolyte composition may also lead to a slight shift in the anolyte potential.

Overall, the lower cell voltage in our system is primarily attributed to the lower catholyte potential of Cr(BIS-TRIS), while differences in anolyte preparation conditions may further contribute to a minor voltage deviation.

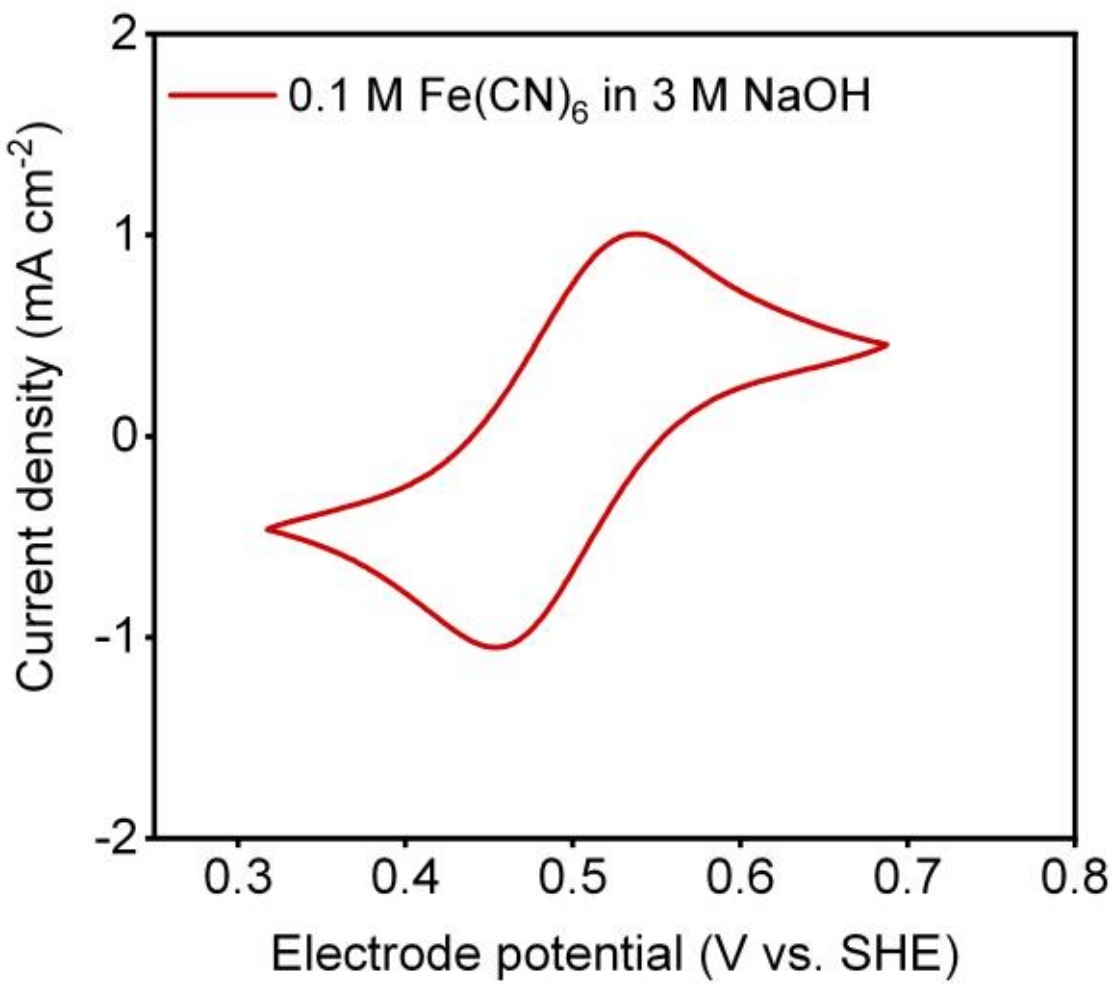

Fig. R7. CV curve of 0.1 $Fe(CN)_6$ at a scan rate of 50 mV s^{-1}.

Comment 3-6: P4: There is no coordinating water in Cr(BIS-TRIS) as for Fe(BIS-TRIS)? How come you detect Cr(BIS-TRIS)OH fragment in HRMS? A crystal structure of the material would be highly informative.

Response: We thank the reviewer for this valuable comment. Our structural analysis of the Cr(BIS-TRIS) complex follows the ESI-HRMS approach reported by Shin et al. for the Fe(BIS-TRIS) chelate (*Chem. Eng. J.* **2023**, 453, 139738). Specifically, the proposed structure of the Fe(BIS-TRIS) complex, based on previously reported iron chelate systems, consists of a 1:1 coordination between Fe^{3+} and the BIS-TRIS ligand with an additional hydroxide ligand, without coordinated water molecules. The corresponding theoretical *m/z* values were calculated using ChemDraw. The experimentally observed ESI-HRMS spectra matched well with the predicted values, thereby supporting the proposed structure.

In this work, we evaluated the structure of the Cr(BIS-TRIS) chelate following the same approach. In alkaline media, BIS-TRIS typically coordinates with metal ions to form complexes consisting of a metal center, one BIS-TRIS ligand, and an additional hydroxide ligand, as reported for Fe(BIS-TRIS) and related complexes (e.g., Mn(BIS-TRIS), Ni(BIS-TRIS), and Co(BIS-TRIS) chelates) (*Dalton Trans.* **2008**, 6, 731–733; *Dalton Trans.* **2009**, 1, 41–50; *Inorg. Chem.* **2019**, 58, 14876–14884). Based on these precedents, we expected Cr(BIS-TRIS) to adopt a coordination motif similar to that of Fe(BIS-TRIS). Accordingly, the expected *m/z* values of the Cr(BIS-TRIS) species were calculated using ChemDraw. ESI-HRMS detects a $[Cr(BIS\text{-}TRIS)(OH)]^{2-}$ fragment at $m/z = 274.0609$, with an isotopic distribution that closely matches the simulated pattern (**Fig. R8**a–c), confirming the expected

molecular composition. These results support a coordination structure in which a Cr center is coordinated by one BIS-TRIS ligand with an additional hydroxide ligand. Furthermore, Raman and Fourier-transform infrared (FTIR) spectroscopy indicate that the BIS-TRIS ligand coordinates to the Cr center through both nitrogen and oxygen donor atoms (**Fig. R8**d,e), providing additional evidence consistent with the structure suggested by the ESI-HRMS analysis.

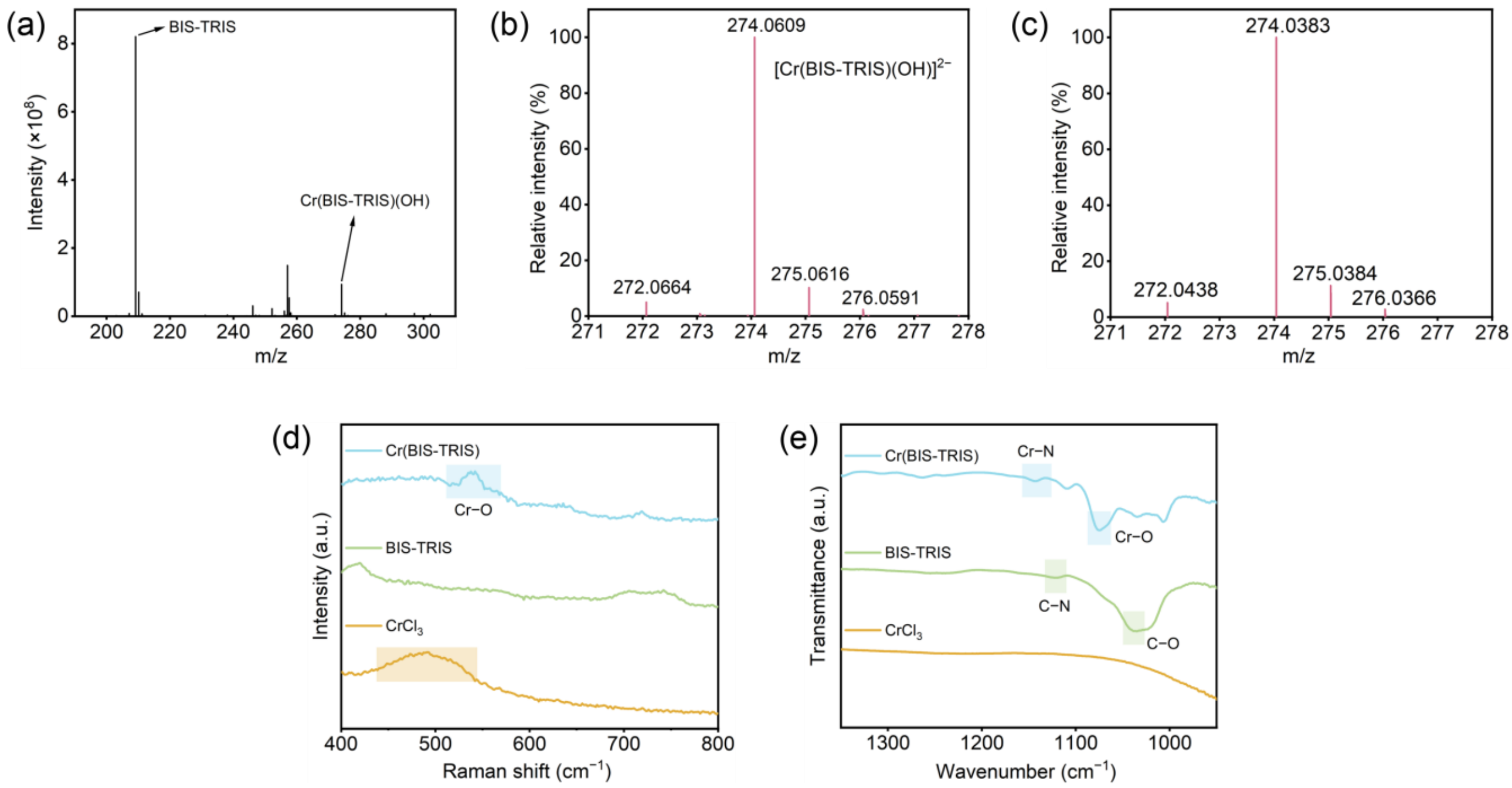

Fig. R8. (a)–(b) Experimentally observed and (c) simulated ESI-HRMS spectra of Cr(BIS-TRIS). (d) Raman and (e) FTIR spectra of Cr(BIS-TRIS), BIS-TRIS, and $CrCl_3$ solutions.

We agree that obtaining a crystal structure would provide more direct structural insight. However, despite numerous attempts using various crystallization methods, including slow evaporation, vapor diffusion, and solvent diffusion, suitable single crystals for X-ray diffraction could not be obtained. This difficulty likely arises from the strong hydrophilicity and high solubility of the Cr(BIS-TRIS) chelate in strongly alkaline aqueous media. As an alternative, we followed recently reported studies (*Nat. Commun.* **2026**, 17, 1119; *Nat. Commun.* **2024**, 15, 2566; *Angew. Chem. Int. Ed.* **2024**, 63, e202316593) and employed synchrotron X-ray absorption fine structure spectroscopy (XAFS) to probe the coordination structure of the Cr(BIS-TRIS) chelate. XAFS is a powerful technique capable of providing detailed information about the coordination environment of the absorbing atom, including the identity of coordinating atoms, bond distances, and coordination numbers. Importantly, XAFS

measurements are performed directly in solution, enabling characterization of the metal-ligand coordination structure under realistic electrolyte conditions. Since our study focuses on the coordination structure of Cr(BIS-TRIS) in aqueous alkaline electrolytes relevant to flow battery operation, XAFS provides a more representative description of the coordination environment in solution.

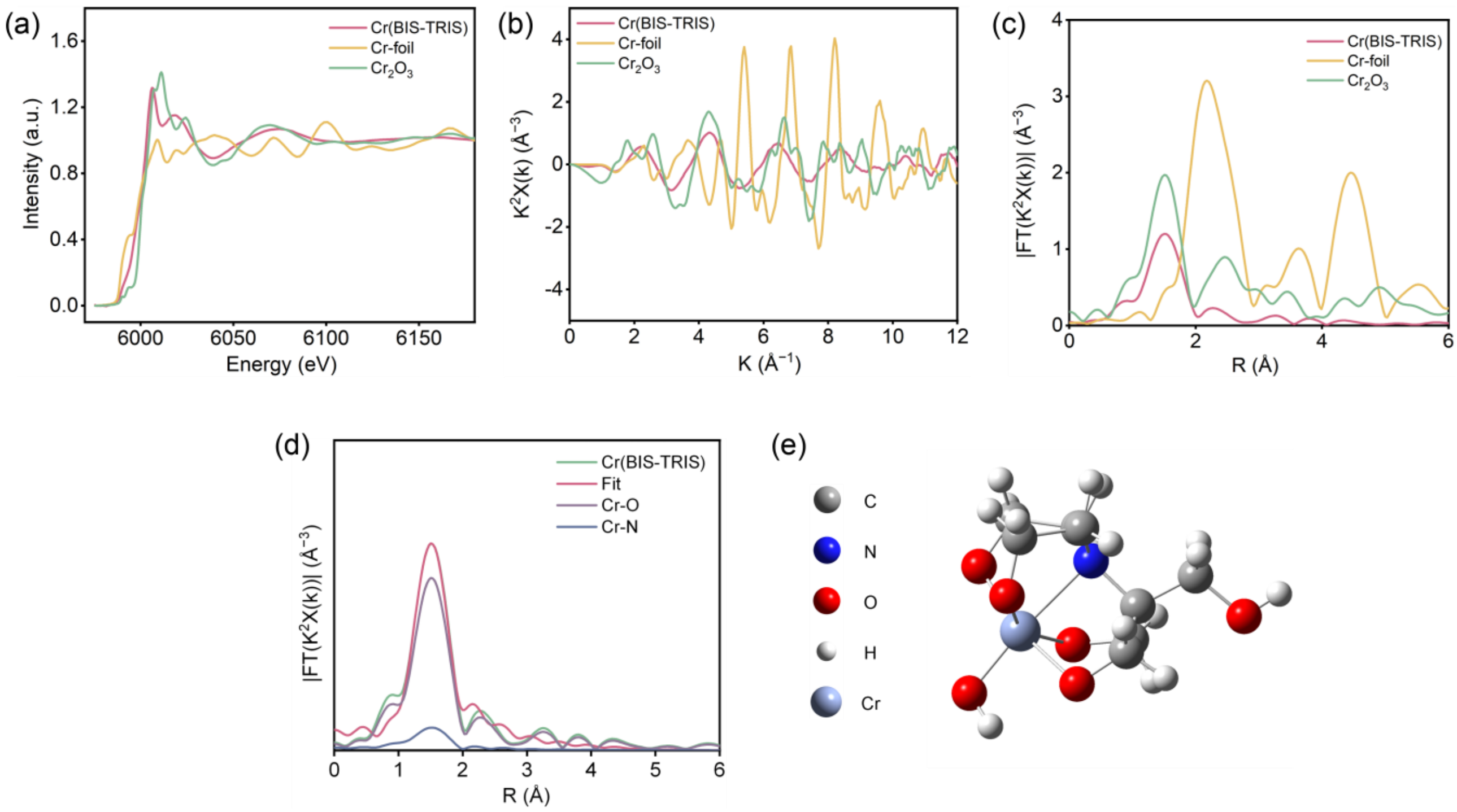

Fig. R9. (a) XAS spectra of Cr(BIS-TRIS) and reference samples Cr foil and Cr_2O_3. (b) Fourier transforms of the Cr K-edge EXAFS of Cr(BIS-TRIS) and reference samples Cr foil and Cr_2O_3 in K-space. (c) Fourier-transformed experimental EXAFS spectra of Cr(BIS-TRIS), Cr foil, and Cr_2O_3 in R-space. (d) Fourier-transformed EXAFS fitting curves of Cr(BIS-TRIS). (e) Structure of Cr(BIS-TRIS).

To further verify the coordination structure, XAFS measurements were performed, and the extended X-ray absorption fine structure (EXAFS) region was analyzed in detail (**Fig. R9**). The local coordination environment around the Cr center was determined by least-squares fitting of the Fourier-transformed EXAFS spectra, with fitting parameters summarized in **Supplementary Table 1**. The EXAFS fitting results reveal that the Cr(BIS-TRIS) chelate contains five Cr-O bonds with an average bond distance of 1.95 Å and one Cr-N bond with a bond distance of 1.99 Å. The reliability of the fitting was further validated through analysis of coordination numbers, bond lengths, Debye-Waller factors,

and fitting residuals. Specifically, the fitted Cr-O coordination number is 4.98 ± 0.5, which overlaps with the expected value of five within the fitting uncertainty, while the Cr-N coordination number is 1.11 ± 0.1, consistent with the expected value of one. The total coordination number is therefore 6.09 ± 0.51, in excellent agreement with the anticipated six-coordinate structure. The fitted bond lengths are also consistent with known chromium coordination chemistry. The Cr-O bond length of 1.95 ± 0.09 Å overlaps with the reference value of Cr_2O_3 (1.98 ± 0.06 Å), while the Cr-N bond length of 1.99 ± 0.09 Å falls within the typical Cr^{3+}-N bond length range of 1.95–2.05 Å. The Debye-Waller factors are positive and within reasonable ranges (0.0023 $Å^2$ for Cr-O and 0.0087 $Å^2$ for Cr-N), and the fitting quality is high (R-factor = 0.011), indicating excellent agreement between the experimental data and the proposed structural model.

Taken together, the ESI-HRMS analysis, Raman and FTIR spectroscopy, and solution-phase XAFS characterization consistently support that Cr(BIS-TRIS) adopts a 1:1 coordination between Cr^{3+} and the BIS-TRIS ligand with an additional hydroxide ligand, forming a six-coordinate structure composed of one nitrogen donor and five oxygen donors. These results provide a coherent description of the coordination environment of Cr(BIS-TRIS) under alkaline electrolyte conditions. We hope this explanation satisfactorily addresses the reviewer's concern.

Supplementary Table 1. EXAFS data fitting results of samples.

Sample	*Shell*	*CN[a]*	*R(Å)[b]*	*σ^2 ($Å^2$)[c]*	*ΔE_0(eV)[d]*	*R factor*
Cr-foil	*Cr-Cr*	*12**	*2.49±0.05*	*0.0069*	*5.5±0.2*	*0.013*
Cr_2O_3	*Cr-O*	*6**	*1.98±0.06*	*0.0012*	*3.5±0.3*	*0.007*
	Cr-O-Cr	*6**	*2.94±0.01*	*0.0082*		
Cr(BIS-TRIS)	*Cr-O*	*4.98±0.5*	*1.95±0.09*	*0.0023*	*2.0±0.6*	*0.011*
	Cr-N	*1.11±0.1*	*1.99±0.09*	*0.0087*		

[a]CN: coordination numbers; [b]R: bond distance; [c]σ^2: Debye-Waller factors; [d] ΔE_0: the inner potential correction. R factor: goodness of fit. Error bounds that characterize the structural parameters obtained by EXAFS spectroscopy were estimated as CN±20%; R ± 1%; σ^2 ± 20%.

<u>Comment 3-7:</u> **P5: "facilitating facile electron transfer20,29,35" in addition to the cited references,**

and for the discussion of XPS data, this study may be of interest to the authors: https://chemistry-europe.onlinelibrary.wiley.com/doi/full/10.1002/batt.202500250.

Response: We thank the reviewer for this helpful suggestion and for bringing this relevant study to our attention. We have carefully reviewed the suggested reference and revised the corresponding discussion on Page 8 of the **Revised Manuscript** accordingly. The modified text is shown below.

*"XPS analysis further supports this valence transition. The positive shift of the Cr 2p binding energies in the charged electrolyte indicates a more electron-deficient Cr center, consistent with the formation of the higher oxidation state[35]. In the discharged state, the Cr 2p spectrum shows dominant peaks at binding energies of 576.1 eV and 585.3 eV, which are assigned to Cr(III)(BIS-TRIS). After charging, new peaks emerge at higher binding energies of 577.5 eV and 587.2 eV, characteristic of Cr(IV)(BIS-TRIS)[39-41] (**Supplementary Fig. 11**)."*

*"35. Thurston, J. R. et al. Electronic structure distortions in chromium chelates impair redox kinetics in flow batteries. Batteries Supercaps, **8**, e202500250 (2025)."*

Comment 3-8: Fig 2a-c: From the text it is not evident to me how these ligands/solutions were assessed. Furthermore, it is inappropriate to classify aminopolycarboxylates and aminopolyphosphonates as weak ligands.

Response: We thank the reviewer for this valuable comment. We have provided additional details on how the ligands were evaluated on Page 6 of the **Revised Manuscript**. In addition, the description of aminopolycarboxylates and aminopolyphosphonates as "weak ligands" has been revised to "moderate ligands". The revised text is shown below.

"The electrochemical performance of chelated electrolytes is primarily governed by their coordination stability and redox reversibility. For an effective alkaline Fe-Cr RFB, the Cr chelate must (i) maintain robust coordination stability in alkaline media without metal-ligand dissociation and (ii) exhibit reversible and kinetically facile redox behavior. Guided by these design principles, we systematically screened a series of multidentate ligands to identify an optimal coordinating framework capable of stabilizing Cr species under highly basic conditions. Because metal centers are generally stabilized by amine-based multidentate backbones, we further classified the candidate ligands

*according to the nature of their auxiliary donor groups, namely carboxylate, phosphate, and hydroxyl groups. This classification yields three representative ligand families: amino-polycarboxylic, amino-polyphosphate, and amino-polyhydroxy ligands. Ten multidentate ligands from each class were evaluated comparatively (**Supplementary Figs. 2–4**), and representative examples are presented in **Fig. 2a–c**.*

*We first evaluated the ability of different classes of ligands to stabilize Cr ions under strongly alkaline conditions (pH = 14). Among the three ligand classes, amino-polycarboxylate and amino-polyphosphonate ligands exhibited poor coordination stability, leading to the formation of Cr hydroxide precipitates in alkaline media. This instability arises because carboxylate and phosphonate oxygen donors undergo charge delocalization or dispersion, which reduces the local electron density at individual oxygen sites. Consequently, hydroxide ions can competitively displace the donor atoms, leading to metal-ligand bond cleavage, ligand dissociation, and eventual precipitation of Cr hydroxides (**Fig. 2d**, **Supplementary Figs. 2,3,5,6**). Therefore, although these ligands can form stable chelates with metal ions under near-neutral conditions, they are unsuitable for alkaline RFB applications. In contrast, amino-polyhydroxy ligands (e.g., diethanolamine (DEA), triethanolamine (TEA), and BIS-TRIS) displayed significantly enhanced coordination stability under identical alkaline conditions (**Fig. 2d**, **Supplementary Fig. 4**). In strongly basic media, the hydroxyl groups of these ligands are deprotonated to form alkoxide species (–O$^-$), in which the negative charge is localized on a single oxygen atom[19,20,31]. This charge localization increases the local electron density and strengthens the σ-donor capability of the coordinating oxygen, thereby reinforcing metal-ligand bonding and maintaining the structural integrity of the Cr chelates in strongly alkaline solution."*

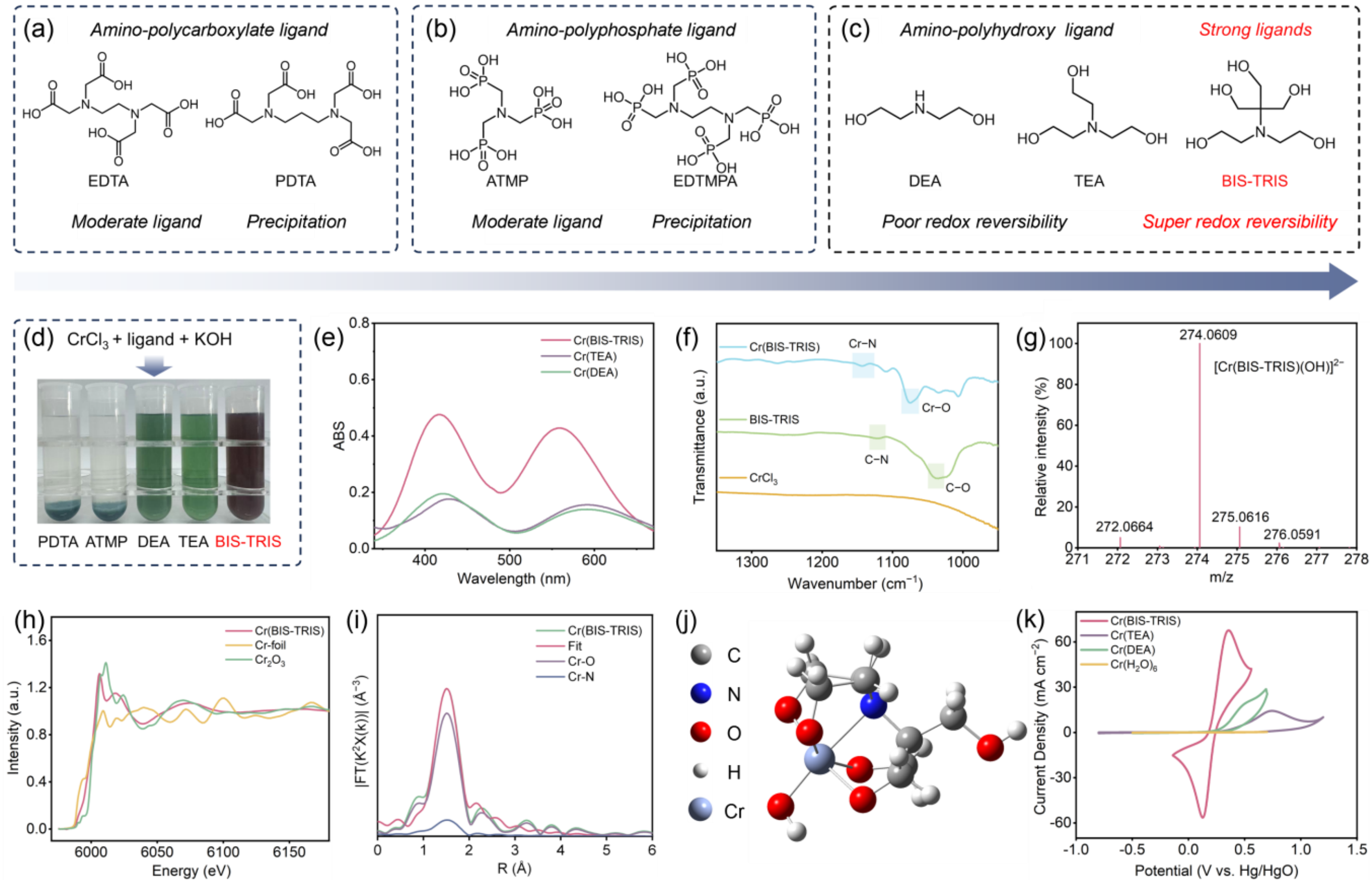

Fig. 2. Rational design and structural characterization of the Cr(BIS-TRIS) chelate. (a–c) Molecular structures of (a) ethylenediaminetetraacetic acid (EDTA) and PDTA, (b) nitrilotrimethylene triphosphonic acid (ATMP) and ethylenediamine tetra(methylenephosphonic acid) (EDTMPA), and (c) DEA, TEA, and BIS-TRIS. (d) Optical photographs of solutions of $CrCl_3$ combined with various ligands under alkaline conditions (pH = 14). (e) UV-Vis spectra of Cr complex solutions formed with various amino-polyhydroxy ligands. (f) FTIR spectra of Cr(BIS-TRIS), BIS-TRIS, and $CrCl_3$ solutions. (g) ESI-HRMS spectrum of Cr(BIS-TRIS) solutions, the peak found at m/z = 274.0609 is assigned to $[Cr(BIS\text{-}TRIS)(OH)]^{2-}$ (calcd: 274.0383). (h) XAS spectra of Cr(BIS-TRIS) and reference samples Cr foil and Cr_2O_3. (i) Fitting EXAFS spectra of Cr(BIS-TRIS) in R-space. (j) Structure of Cr(BIS-TRIS) chelate. (k) CV curves of 0.5 M Cr(DEA), Cr(TEA), Cr(BIS-TRIS), and $CrCl_3$ solutions at 50 mV s^{-1}.

Comment 3-9: . **P6: Why does a narrower HOMO-LUMO gap enhance electron-transfer "propensity"?**

Response: We sincerely thank the reviewer for raising this important point. In response, we have made efforts to further clarify the relationship between the HOMO-LUMO gap and the electron transfer kinetics of the complexes.

HOMO (the highest occupied molecular orbital) and LUMO (the lowest unoccupied molecular orbital) represent the frontier orbitals that define the electronic structure of a molecular system. The energy difference between them, known as the HOMO-LUMO gap, reflects the energy required to promote an electron from an occupied orbital to an unoccupied one and is therefore an important descriptor of the accessibility of electronic excitation and charge transfer. In general, a smaller HOMO-LUMO gap indicates that less energy is required for electron promotion between frontier orbitals, making electronic excitation or charge redistribution more readily achievable.

In redox reactions, the energy levels of HOMO and LUMO are closely related to the tendency of a species to lose or gain electrons. A higher HOMO energy facilitates electron removal and thus favors oxidation, whereas a lower LUMO energy facilitates electron acceptance and thus favors reduction. For metal complexes, the key step in electron transfer typically involves the promotion of an electron between a filled frontier orbital (HOMO) and an empty one (LUMO). When the HOMO-LUMO gap decreases, the energy required for such electronic transitions is reduced, making electron transfer energetically more accessible and resulting in a stronger charge-transfer propensity(*J. Mol. Model.* **2024**, 30, 122; *Russ. J. Electrochem* **2021**, 57, 412–418).

Furthermore, for transition metal complexes, a narrower frontier orbital gap is often associated with more favorable donor-acceptor orbital alignment and stronger orbital interactions, which facilitate electron transfer between different reaction centers and lead to faster electron-transfer kinetics. This trend is also consistent with the framework of Marcus electron-transfer theory. According to Marcus theory, the rate of electron transfer is governed not only by the thermodynamic driving force but also by the electronic coupling between donor and acceptor states as well as the reorganization energy. In particular, stronger electronic coupling generally lowers the activation barrier for charge transfer, thereby enhancing the electron-transfer rate (*Rev. Mod. Phys.* **1993**, 65, 599-610).

We hope that this further explanation addresses the reviewer's concerns and provides additional clarity regarding the relationship between the HOMO-LUMO gap and the electron transfer behavior of the complexes. Related modifications have been added on page 4 in the **Revised Manuscript** as follows:

"A narrower band gap between the highest occupied molecular orbital (HOMO) and the lowest unoccupied molecular orbital (LUMO) indicates a stronger ability to transfer electrons, as it facilitates faster oxidation and reduction processes through stronger electronic coupling and lower activation

barriers, respectively."

Comment 3-10: **P7: "high electron density of deprotonated oxygen" --> again, same logic applies to EDTA, PDTA.**

Response: We thank the reviewer for this valuable comment. The sentence has been revised accordingly and now reads:

"This feature arises from the high local electron density of the alkoxide species within the amino-polyhydroxy ligand framework."

Comment 3-11: **P8: remarkable reversibility --> unnecessary hyperbolic language. Cr(BIS-TRIS) is described as highly reversible but the peak separation in CV is 229 mV, which is hardly highly reversible.**

Response: Thank the reviewer for this valuable comment. In the **Revised Manuscript**, the description has been modified and the term "highly reversible" has been replaced with "quasi-reversible".

Comment 3-12: **P8: How do D0 and k0 highlight "alkaline stability"?**

Response: We thank the reviewer for this valuable comment. The phrase "alkaline stability" has been removed, and the description has been revised on Page 10 of the **Revised Manuscript** accordingly.

*"From scan rate-dependent CVs, its diffusion coefficients (D_O) and standard rate constants (k_O) were determined **(Fig. 4c, Supplementary Figs. 17,18)**. Cr(BIS-TRIS) exhibits D_O of 6.93×10^{-6} cm^2 s^{-1} and k_O of 6.85×10^{-3} cm s^{-1}, representing a 1000-fold enhancement over uncatalyzed Cr(II/III) in $CrCl_3$ (**Supplementary Table 4**)[24]. These results highlight its rapid kinetics, positioning Cr(BIS-TRIS) as an outstanding catholyte for Fe-Cr RFBs. For comparison, Fe(BIS-TRIS) delivers D_O of 3.03×10^{-6} cm^2 s^{-1} and k_O of 2.79×10^{-3} cm s^{-1}, confirming its suitability as an efficient anolyte. "*

Comment 3-13: **. P10: The description of evolving negolyte imbalance is nice.**

Response: We thank the reviewer for the positive feedback.

Comment 3-14: **P10: Ligand-to-metal ratio studies show that free ligand reacts with the charged posolyte species, so having free ligand is a bad idea. Why not synthesize pure Cr(BIS-TRIS) and prepare the electrolyte from that without free ligand? This is a key concern I have with this study (and most metal complex RFB studies for that matter): Synthesizing the electrolyte in-situ yields a highly skewed picture. What happens to the chloride ions from $CrCl_3$? How do they affect solubility and electrochemical properties?**

To describe a new material I would expect synthesis of the pure compound, i.e. isolate KCr(BIS-TRIS), characterize it, and then prepare RFB electrolytes from it. This criticism applies to almost every paper out there regarding e.g., Fe(BIS-TRIS), Fe(TEA), Fe(DIPSO), Ce(DTPA) etc. Can you clarify if the active material is KCr(BIS-TRIS) or K2Cr(BIS-TRIS) for example?

Can you provide experiments without free ligand to prove that there is no irreversible chelate decomposition? How does this affect Coulombic efficiency and capacity retention? According to your results the problem should be resolved by not having any free ligand?

Response: We thank the reviewer for this insightful comment. Our response is provided in three parts.

(1) Response regarding the electrolytes without free ligand

We agree that, from an ideal stoichiometric perspective, a 1:1 metal-to-ligand ratio with minimal free ligand would be desirable. However, in aqueous alkaline media, the formation of the Cr(BIS-TRIS) chelate is governed by a dynamic and reversible coordination equilibrium, as described below:

$$Cr^{3+} + \text{BIS-TRIS} + 5OH^{-} \rightleftharpoons [Cr(\text{BIS-TRIS})(OH)]^{2-} + 4H_2O \quad (2)$$

Because this coordination process is reversible, a certain amount of free ligand is required to shift the equilibrium toward the coordinated Cr(BIS-TRIS) species and to maintain complex stability under strongly alkaline conditions. From the perspective of chemical equilibrium, an electrolyte completely free of unbound ligand is not feasible. Even if an isolated 1:1 Cr(BIS-TRIS) solid could be synthesized, the same issue would re-emerge once it is dissolved in alkaline aqueous media, because the coordination equilibrium would be re-established in solution. As a result, a certain excess of BIS-TRIS is still required to stabilize the coordinated species and suppress hydrolysis.

This conclusion is also supported experimentally. When the electrolyte was prepared using a strict 1:1 molar ratio of Cr^{3+} to BIS-TRIS, visible precipitation was observed (**Fig. R10**a), indicating that a fraction of Cr^{3+} remained insufficiently coordinated and underwent hydrolysis to form insoluble $Cr(OH)_3$. As a result, part of the Cr^{3+} is removed from the solution, while free ligand still remains in the electrolyte. Such behavior is commonly observed in metal-ligand coordination systems, where a moderate excess of ligand is necessary to drive complex formation and suppress competing side reactions (*Nat. Commun.* **2026**, 17, 1119; *Nat. Commun.* **2024**, 15, 2566). Therefore, it is not feasible to maintain a stable electrolyte entirely free of unbound ligand under these conditions.

(2) Response regarding the effect of ligand-to-metal ratio on CE and cycling stability

We systematically investigated the effect of ligand-to-metal ratio on Coulombic efficiency (CE) and cycling stability under fully charged conditions (100% SOC) at a current density of 100 mA cm^{-2}. Electrolytes with ligand-to-metal ratios from 1:1 to 1.3:1 were evaluated in balanced cells.

At a 1:1 ratio, the ligand is insufficient to fully stabilize all Cr species, leading to partial hydrolysis and formation of $Cr(OH)_3$ precipitates (**Fig. R10**a). After filtration, the resulting electrolyte exhibits a slightly reduced capacity due to the loss of active chromium species. As the ligand-to-metal ratio increases from 1:1 to 1.1:1, 1.2:1, and 1.3:1, the average CE decreases from 98.7% to 98.3%, 97.7%, and 96.9%, respectively, and the capacity decay rate correspondingly increases (**Fig. R10**b). This trend is consistent with the degradation mechanism proposed in this work, namely that the charged Cr(IV)(BIS-TRIS) species oxidizes the free ligand in the catholyte, leading to charge imbalance between the two electrolytes and consequent capacity decay (**Fig. R10**c,d). Accordingly, the lost capacity can be fully recovered when sufficient free ligand is present to ensure stable chelation. As shown in **Fig. R10**e, for the 1.1:1 system, the capacity can be fully restored to its initial value after 100 cycles upon introducing oxygen into the anolyte. However, when the amount of free ligand is too low, the stability of the chelate is compromised, resulting in partial chelate decomposition. For example, in the 1:1 system, the capacity can only be restored to ~90% after 100 cycles upon oxygen introduction (**Fig. R10**e), indicating that a small degree of irreversible chelate decomposition occurs under this condition, as also evidenced by the optical image of the cycled 1:1 electrolyte.

These results demonstrate that increasing free ligand content leads to lower CE and faster capacity decay. However, eliminating free ligand is not viable, as insufficient ligand results in incomplete

coordination and precipitation. Therefore, further optimization of ligand design, particularly to improve oxidation resistance (e.g., by replacing C-H bonds with more stable C-F bonds), may help mitigate these effects.

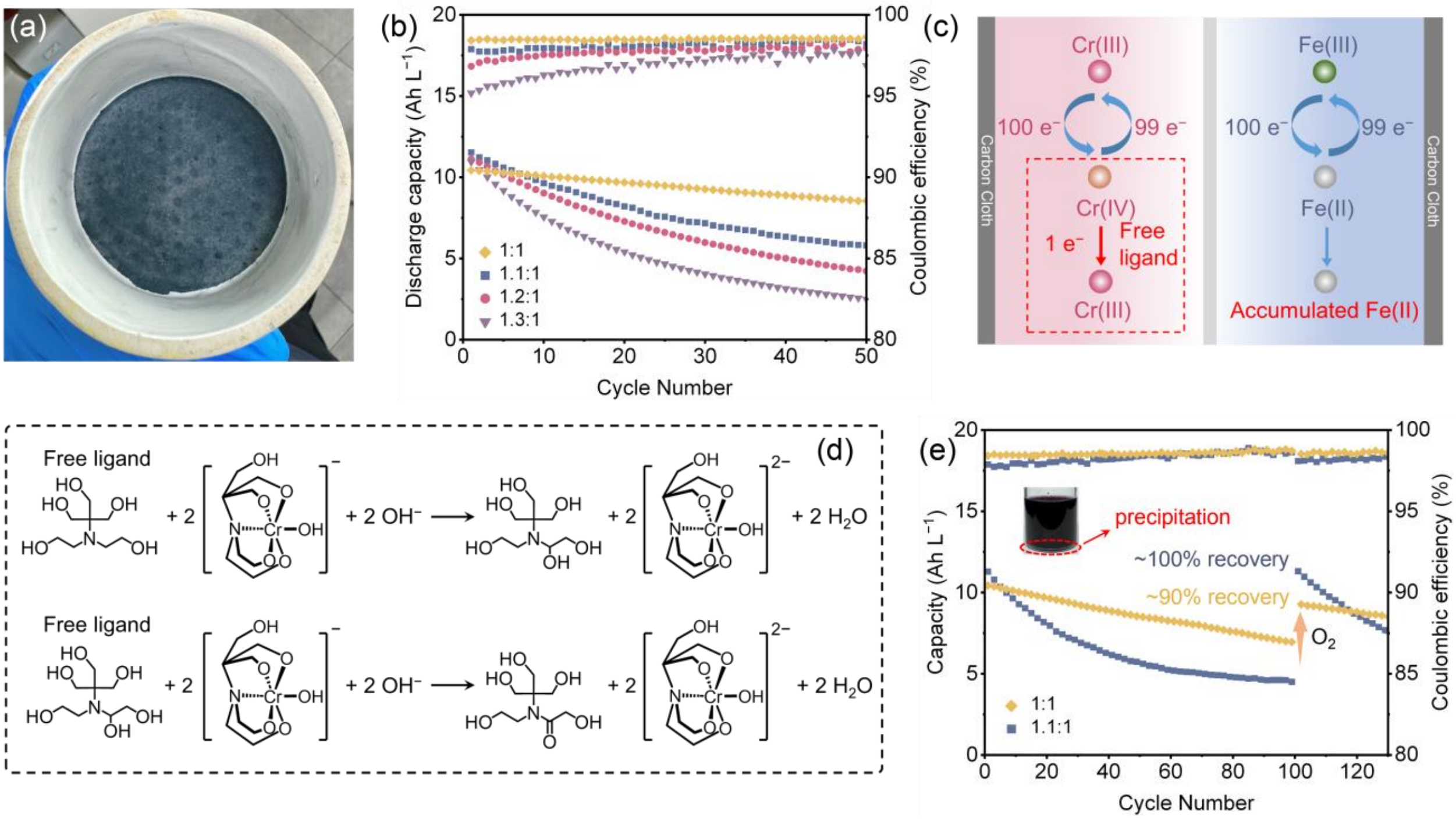

Fig R10. (a) Optical image of the precipitate obtained after filtration of the Cr(BIS-TRIS) electrolyte prepared with a 1:1 ligand-to-metal ratio. (b) Cycling performance of a 0.5 M alkaline Fe-Cr RFB with varying ligand-to-metal ratios in the Cr(BIS-TRIS) catholyte at 100% SOC and 100 mA cm^{-2}. (c) The proposed attenuation mechanism and (d) possible reactions of free BIS-TRIS ligand oxidation. (e) Extended cycling performance and capacity recovery of the 1:1 and 1.1:1 systems upon oxygen exposure of the anolyte, together with an optical image of the electrolyte of the 1:1 system after cycling.

(3) Response regarding role of chloride ions from $CrCl_3$

In the present system, chloride ions introduced from $CrCl_3$ remain as free anions in the alkaline electrolyte and do not participate in the coordination sphere of the Cr(BIS-TRIS) chelate. Under strongly alkaline conditions, the coordination environment is dominated by the multidentate BIS-TRIS ligand and hydroxide ions, which effectively outcompete Cl^- for coordination with Cr centers. As a result, Cl^- ions do not enter the first coordination shell and therefore have negligible influence on the coordination structure of the electroactive species. This is consistent with the XAFS and spectroscopic results discussed above, which show a well-defined Cr(BIS-TRIS) coordination environment without evidence of chloride coordination.

Regarding solubility, the use of chloride salts is advantageous because of their high aqueous solubility, which facilitates electrolyte preparation without altering the speciation of the coordinated complex. Consequently, chloride ions act primarily as non-coordinating counterions in the electrolyte and are expected to have negligible impact on the electrochemical properties of the Cr(BIS-TRIS) system, including redox potential and reversibility.

This conclusion is further supported experimentally. To verify the role of chloride ions, we independently prepared Cr(BIS-TRIS) electrolytes using $CrCl_3$ and $KCr(SO_4)_2$ as the chromium precursors, respectively, and evaluated their battery performance at a ligand-to-metal ratio of 1:1. The corresponding CV curves, Coulombic efficiencies, and cycling stabilities showed almost no discernible difference between the two systems (**Fig. R11**). These results confirm that the identity of the counter-anion does not materially affect the coordination chemistry or electrochemical performance of the Cr(BIS-TRIS) electrolyte. Therefore, the chloride ions derived from $CrCl_3$ can be regarded as spectator anions with negligible influence on the present system.

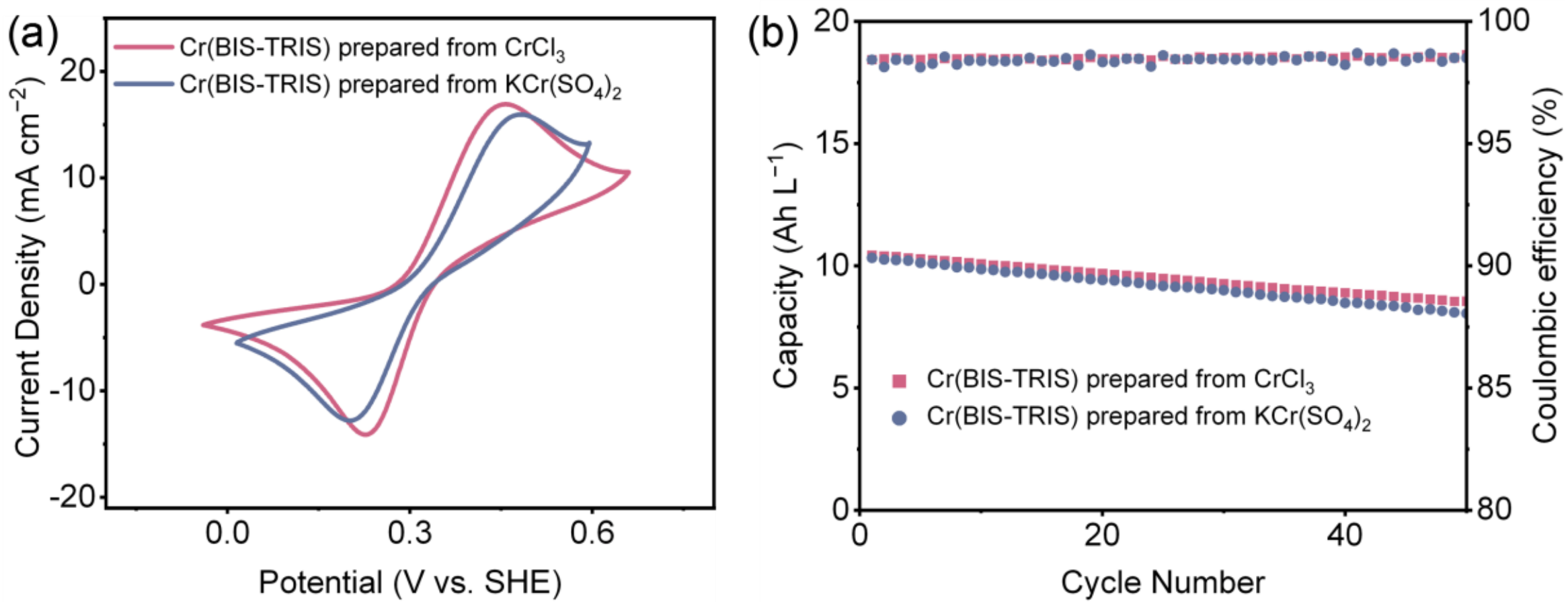

Fig R11. CV curves of Cr(BIS-TRIS) prepared from $CrCl_3$ and $KCr(SO_4)_2$. (b) Cycling performance of full cells using Cr(BIS-TRIS) prepared from $CrCl_3$ and $KCr(SO_4)_2$ at 100% SOC and 100 mA cm^{-2}.

Comment 3-15: What is the upper pH-limit of Cr(BIS-TRIS)?

Response: We thank the reviewer for this insightful question. Within the investigated range, no upper pH limit was observed, and the chelate remains stable up to 10 M KOH (corresponding to a nominal pH of ~15), as shown in **Fig. R12**a. In contrast, at pH < 13, insufficient ligand deprotonation prevents effective chelation, and chromium predominantly exists as insoluble precipitates.

This behavior is governed by the coordination chemistry of BIS-TRIS. Chelate formation requires deprotonation of hydroxyl groups to generate alkoxide ($–O^{-}$) donors, which exhibit stronger σ-donating ability and higher electron density than hydroxide ions, thereby enabling robust multidentate coordination with Cr centers and enhancing stability in alkaline media (**Fig. R12**b). Under highly alkaline conditions, excess OH^{-} does not effectively compete with BIS-TRIS due to the strong chelation effect and stabilized octahedral coordination environment. Instead, sufficiently high pH is essential to maintain ligand activation and ensure stable complex formation.

Therefore, the stability of Cr(BIS-TRIS) is dictated by a lower pH threshold (~13) for ligand deprotonation, while no upper pH limit is observed within the experimentally alkaline regime.

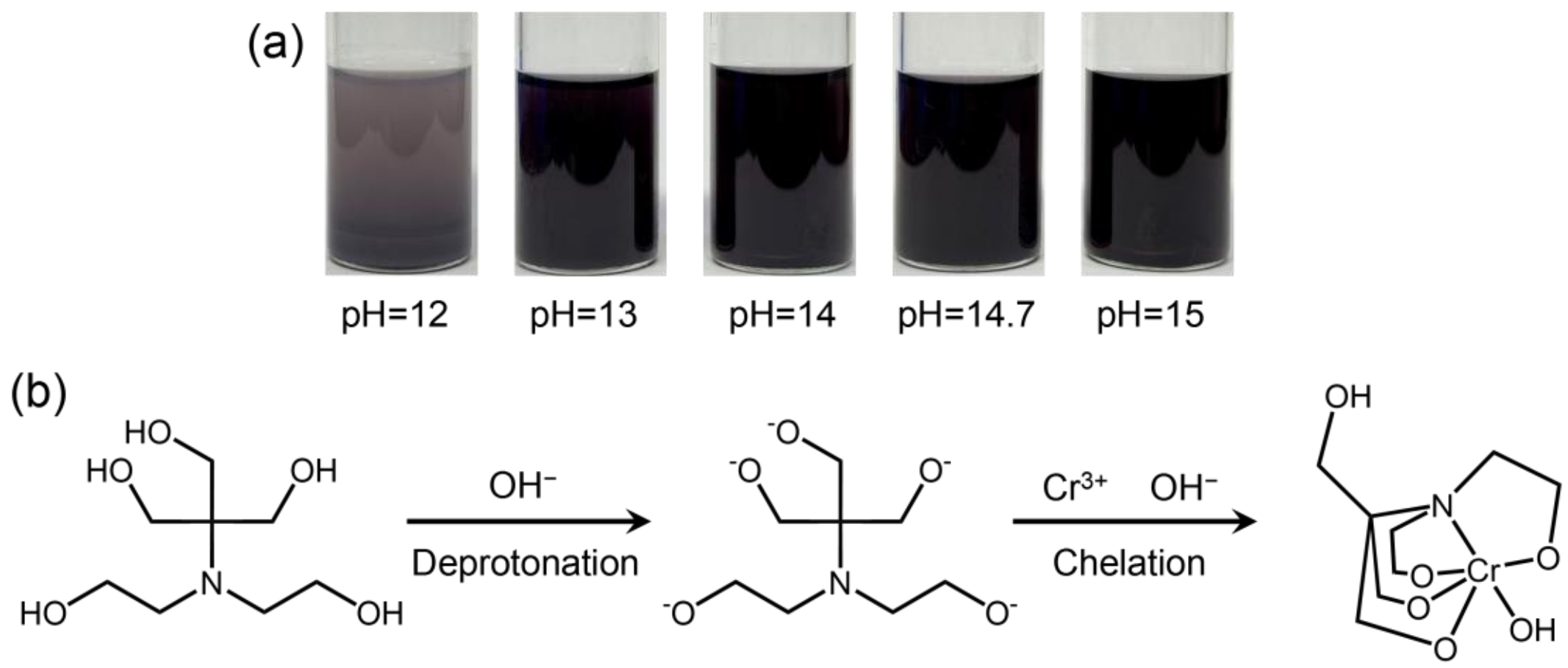

Fig. R12. (a) Optical images of Cr(BIS-TRIS) electrolytes at different pH values. (b) Formation process of the Cr(BIS-TRIS) chelate.

Comment 3-16: **. The authors should acknowledge some of the work by Shin et al. who previously described Fe(BIS-TRIS) in detail: https://onlinelibrary.wiley.com/doi/10.1002/er.7718, https://www.sciencedirect.com/science/article/pii/S1385894722052172, https://www.sciencedirect.com/science/article/pii/S1385894723034137**

Response: We thank the reviewer for this valuable comment. Relevant references have now been added to the revised manuscript and are listed below.

"49. Shin, M. et al. Electrolyte optimization of alkaline aqueous redox flow battery using iron-2,2-bis(hydroxymethyl)-2,2',2'-nitrilotriethanol complex as active material for anolyte. Chem. Eng. J. ***453****, 139738 (2023).*

50. Shin, M. et al. Spectroscopic and electrochemical analyses elucidating capacity degradation mechanism of iron-ligand complex and air in all iron aqueous redox flow batteries. Chem. Eng. J. ***471****, 144682 (2023).*

51. Shin, M. et al. Aqueous redox flow battery using iron 2,2-bis(hydroxymethyl)-2,2',2'-nitrilotriethanol complex and ferrocyanide as newly developed redox couple. Int. J. Energy Res. ***46****, 8175-8185 (2022)."*

List of Changes and Response to the Reviewers' comments

Dear Editors and Reviewers:

We sincerely thank the reviewers for their positive assessment of our work, their support for publication, and their careful and constructive comments. In response, we have revised the manuscript and Supplementary Information and provide below our point-by-point responses to all remaining comments.

We also thank the editors for providing the detailed editorial checklist. We have addressed each item and made the corresponding revisions throughout the manuscript and Supplementary Information. All revisions made in response to the reviewer comments and editorial checklist are highlighted in red.

Response to Reviewers' comments

Reviewer #3:

General Comment:

The authors did an excellent job addressing my questions and concerns and I support publication of the article in Nature Communications after the following minor comments have been resolved.

Response: We sincerely thank the reviewer for the positive assessment of our work and for the valuable suggestions provided to improve our manuscript.

Comment 3-1: **Figure R1/R6 is helpful and should be included in the supporting information.**

Response: We thank the reviewer for this constructive suggestion. We have included Figure R1/R6 in the revised Supplementary Information as **Supplementary Fig. 7** and added the corresponding discussion to the main text. The added text reads: "*In strongly basic media, the hydroxyl groups of these ligands are deprotonated to form alkoxide species (–O$^-$), in which the negative charge is localized on a single oxygen atom (**Supplementary Fig.** 7).*"

Comment 3-2: **Abstract: The authors should specify that the 68.07 Ah/L are per posolyte volume not per total volume of electrolyte.**

Response: We thank the reviewer for this helpful suggestion. We have revised the relevant sections of

the manuscript to clarify that the theoretical capacity of 68.07 Ah L^{-1} is calculated based on the posolyte volume rather than the total electrolyte volume.

Comment 3-3: **L63: PDTA is labelled "strong-field ligand" which is correct, but later in the manuscript it is positioned as (relatively) weak.**

Response: We thank the reviewer for identifying this inconsistency. To avoid this potentially misleading comparison, we have removed the qualitative label "moderate ligand" associated with PDTA from **Fig. 2**a,b. The description of PDTA as a strong-field ligand in the main text has been retained, and the terminology is now consistent throughout the revised manuscript.

Comment 3-4: **L72: "alternatively, alkaline systems..." CrPDTA is also operated in alkaline environments (pH 7-10). Much less alkaline that pH 14 but technically still alkaline.**

Response: We thank the reviewer for this insightful comment. We have revised the text to emphasize the transition from near-neutral or mildly alkaline conditions to strongly alkaline conditions. The revised text now reads: "*Moving from near-neutral or mildly alkaline conditions to strongly alkaline conditions provides a thermodynamically favorable pathway to suppress HER.*"

Comment 3-5: **Figure 2a-c: The authors should remove "moderate ligand" from a) and b) because these ligands are by all means still very strong and industry standard ligands, I think it would be misleading to label them as moderate. In c) the authors should remove "super" because with a peak separation of >200 mV this is, as correctly described in the text, quasi-reversible, not super reversible.**

Response: We thank the reviewer for this valuable comment. Accordingly, we have removed the label "moderate ligand" from **Fig. 2**a,b and replaced "super reversible" with "quasi-reversible" in **Fig. 2**c.

Comment 3-6: **L256: ohmic --> Ohmic.**

Response: We thank the reviewer for pointing this out. We have corrected "ohmic" to "Ohmic" in the revised manuscript.

Comment 3-7: L259: are you sure this is just the membrane resistance, not the total Ohmic resistance?

Response: We thank the reviewer for pointing out this distinction. We agree that the high-frequency intercept represents the total Ohmic resistance rather than the membrane resistance alone and have corrected the terminology accordingly. Nevertheless, owing to the limited ionic conductivity of PFSA membranes under strongly alkaline conditions, the membrane is expected to be the major contributor to the total Ohmic resistance (*Adv. Mater.* 2025, 37, e10023; *Joule* 2022, 6, 884−905). The relevant text has been revised as follows:

"The polarization curve at 60% SOC yielded an area-specific resistance (ASR) of 1.46 Ω cm^2 *(**Supplementary Fig. 21a**), while the high-frequency intercept of the Nyquist plot indicated a total Ohmic resistance of 1.26 Ω cm*^{2} *(**Supplementary Fig. 21b**), corresponding to approximately 86% of the polarization ASR. Although the total Ohmic resistance includes contributions from all cell components, the limited ionic conductivity of the perfluorosulfonic acid (PFSA) membrane under alkaline conditions is expected to constitute its major contribution*[52,53].

Comment 3-8: L277: The authors should point out in the main text that the solubility values are obtained for mixed-cation systems.

Response: We thank the reviewer for this valuable comment. We have clarified that the reported solubilities were obtained in a mixed-cation alkaline electrolyte containing both K^+ and Na^+. The revised text now reads: "*As shown in **Fig. 4g**, the Cr(BIS-TRIS) and Fe(BIS-TRIS) chelates exhibit solubilities of 2.54 M and 2.78 M, respectively, in mixed-cation electrolytes containing 5 M KOH and 5 M NaOH.*"
